# A Single-Run HPLC–MS Multiplex Assay for Therapeutic Drug Monitoring of Relevant First- and Second-Line Antibiotics in the Treatment of Drug-Resistant Tuberculosis

**DOI:** 10.3390/pharmaceutics15112543

**Published:** 2023-10-27

**Authors:** Niklas Köhler, Hande Karaköse, Hans-Peter Grobbel, Doris Hillemann, Sönke Andres, Christina König, Barbara Kalsdorf, Thomas Theo Brehm, Laura Böttcher, Inna Friesen, Harald Hoffmann, Dražen Strelec, Dagmar Schaub, Charles A. Peloquin, Stefan Schmiedel, Laurent A. Decosterd, Eva Choong, Sebastian G. Wicha, Rob E. Aarnoutse, Christoph Lange, Patricia M. Sánchez Carballo

**Affiliations:** 1 Clinical Infectious Diseases, Research Center Borstel, Leibniz Lung Center, 23845 Borstel, Germany; 2 German Center for Infection Research (DZIF), Partner Site Borstel-Hamburg-Lübeck-Riems, 23845 Borstel, Germany; 3 Respiratory Medicine & International Health, University of Lübeck, 23562 Lübeck, Germany; 4 Bioanalytical Chemistry, Research Center Borstel, Leibniz Lung Center, 23845 Borstel, Germany; 5 National and World Health Organization Supranational Reference Laboratory for Mycobacteria, Research Center Borstel, 23845 Borstel, Germany; 6 Department of Intensive Care Medicine, University Medical Center Hamburg-Eppendorf, 20246 Hamburg, Germany; 7 Department of Pharmacy, University Medical Center Hamburg-Eppendorf, 20246 Hamburg, Germany; 8 Division of Infectious Diseases, I. Department of Internal Medicine, University Medical Center Hamburg-Eppendorf, 20246 Hamburg, Germany; 9 German Center for Infection Research (DZIF), Partner Site Hamburg-Lübeck-Borstel-Riems, 20246 Hamburg, Germany; 10 Institute of Microbiology and Laboratory Medicine, World Health Organization Supranational Reference Laboratory of TB, IML red GmbH, 82131 Gauting, Germany; 11 SYNLAB Gauting, SYNLAB MVZ of Human Genetics Munich, 82131 Gauting, Germany; 12 Department for Lung Diseases, Hospital for Lung Diseases and Tuberculosis, 42244 Klenovnik, Croatia; 13 Infectious Disease Pharmacokinetics Laboratory, Emerging Pathogens Institute, University of Florida, Gainesville, FL 32610, USA; 14 Department of Pharmacotherapy and Translational Research, College of Pharmacy, University of Florida, Gainesville, FL 32610, USA; 15 Laboratory of Clinical Pharmacology, Department of Laboratory Medicine and Pathology, Lausanne University Hospital and University of Lausanne, 1011 Lausanne, Switzerland; 16 Institute of Pharmacy, University of Hamburg, 20246 Hamburg, Germany; 17 Department of Pharmacy, Radboud Institute for Medical Innovation, Radboud University Medical Center, 6525 GA Nijmegen, The Netherlands; 18 Baylor College of Medicine and Texas Childrens’ Hospital, Houston, TX 77030, USA

**Keywords:** TDM, levofloxacin, moxifloxacin, bedaquiline, linezolid, clofazimine, terizidone, cycloserine, delamanid, meropenem

## Abstract

The treatment of drug-resistant *Mycobacterium tuberculosis* relies on complex antibiotic therapy. Inadequate antibiotic exposure can lead to treatment failure, acquired drug resistance, and an increased risk of adverse events. Therapeutic drug monitoring (TDM) can be used to optimize the antibiotic exposure. Therefore, we aimed to develop a single-run multiplex assay using high-performance liquid chromatography–mass spectrometry (HPLC–MS) for TDM of patients with multidrug-resistant, pre-extensively drug-resistant and extensively drug-resistant tuberculosis. A target profile for sufficient performance, based on the intended clinical application, was established and the assay was developed accordingly. Antibiotics were analyzed on a zwitterionic hydrophilic interaction liquid chromatography column and a triple quadrupole mass spectrometer using stable isotope-labeled internal standards. The assay was sufficiently sensitive to monitor drug concentrations over five half-lives for rifampicin, rifabutin, levofloxacin, moxifloxacin, bedaquiline, linezolid, clofazimine, terizidone/cycloserine, ethambutol, delamanid, pyrazinamide, meropenem, prothionamide, and para-amino salicylic acid (PAS). Accuracy and precision were sufficient to support clinical decision making (≤±15% in clinical samples and ±20–25% in spiked samples, with 80% of future measured concentrations predicted to fall within ±40% of nominal concentrations). The method was applied in the TDM of two patients with complex drug-resistant tuberculosis. All relevant antibiotics from their regimens could be quantified and high-dose therapy was initiated, followed by microbiological conversion. In conclusion, we developed a multiplex assay that enables TDM of the relevant first- and second-line anti-tuberculosis medicines in a single run and was able to show its applicability in TDM of two drug-resistant tuberculosis patients.

## 1. Introduction

Tuberculosis (TB) is an airborne infection with *Mycobacterium tuberculosis* and is the leading cause of death due to a single bacterial pathogen worldwide. According to the latest World Health Organization (WHO) estimates, 10.6 million individuals developed TB and approximately 1.6 million died from this disease in 2021 [1]. Drug-susceptible TB is treated with a combination regimen of the first-line drugs rifampicin, isoniazid, pyrazinamide, and ethambutol. The fight against TB is challenged by the emergence of antimicrobial drug resistance. Multidrug-resistant TB (MDR-TB) is defined by *M. tuberculosis* resistance to rifampicin and isoniazid. As rifampicin-resistant TB (RR-TB) serves also as an indicator for isoniazid-resistant TB, the WHO has accepted RR-TB as a surrogate for MDR-TB and categorizes both as MDR/RR-TB [2]. Additional resistance to moxifloxacin or levofloxacin is defined as pre-extensively drug-resistant TB (pre-XDR-TB), and further *M. tuberculosis* resistance to bedaquiline and/or linezolid is defined as extensively drug-resistant TB (XDR-TB) [2].

**Figure 1:**
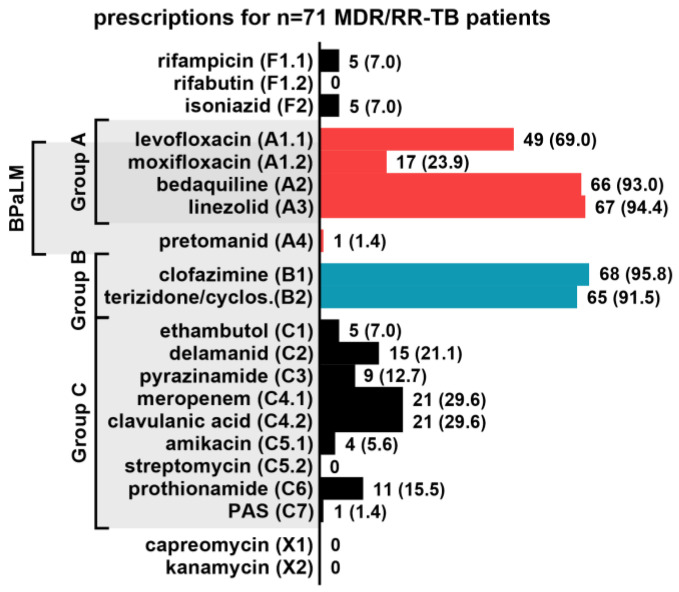
WHO priority ranking of anti-TB drugs and frequency of drug prescriptions as part of drug-resistant tuberculosis treatment regimens. Prescriptions to 71 patients with MDR/RR-TB, pre-XDR-TB and XDR-TB between August 2018 and August 2021 at the Medical Clinic of the Research Center Borstel, Germany.

The level of *M. tuberculosis* drug resistance determines the choice of drugs for a treatment regimen. Since 2022, the WHO recommends a 6-month short-course regimen of bedaquiline, pretomanid, linezolid, and moxifloxacin (BPaLM) for the treatment of MDR/RR-TB, and of bedaquiline, pretomanid and linezolid (BPaL) for pre-XDR-TB. In cases of XDR-TB or pretomanid resistance, or when short-course therapy is not available or not tolerated, a longer 18-month regimen with four to five second-line drugs that are selected hierarchically is recommended (Figure 1). The WHO categorizes second-line drugs in three groups, A, B, and C, according to their effectiveness. Ideally, all group A and one or two group B antibiotics are included in the longer regimen. If drug resistances, adverse events, intolerances, or unavailability prohibit the use of one of these drugs, antibiotics from group C are added to complete the regimen [3].

Presently, the recommended doses of these antibiotics in adults follow a “one dose fits all” principle. Yet, this practice does not consider patient-to-patient variability in absorption, distribution, metabolism, and excretion of the TB drugs. This variability coincides with an often narrow therapeutic window. This means that too low plasma drug concentrations can lead to ineffective treatment and ultimately treatment failure and acquired drug resistance [4], whereas too high plasma drug concentrations can increase the risk of adverse events and treatment interruptions [5,6]. Both too high and too low concentrations can be avoided by therapeutic drug monitoring (TDM). TDM is the measurement of drug concentrations in the plasma or serum of patients in order to individually adjust the dose of these drugs according to reference values [7]. High-performance liquid chromatography–mass spectrometry (HPLC–MS) is a standard technique for drug concentration measurements in TDM. Chromatographic separation and multiple reaction monitoring (MRM) mass spectrometry allow researchers to measure the concentrations of several drugs simultaneously in the same run, i.e., in a multiplex assay. There are many multiplex assays for first-line drugs [8] and TDM for drug-susceptible TB has become available, at least in high-resource settings [9]. Even though not a standard of care yet, it is used to optimize response and explain suboptimal response, to prevent the emergence of resistance, to prevent and explain adverse effects related to concentrations, and to detect and manage drug–drug interactions. The TDM practice of TB drugs is currently confined to patients who are (or appear) at risk for deviating drug concentrations, such as patients with delayed response, relapse TB, gastrointestinal abnormalities, diabetes mellitus, HIV, malnutrition, or renal dysfunction [10,11].

In MDR/RR-, pre-XDR- and XDR-TB, TDM is recommended to ensure the efficacy or avoid toxicity of moxifloxacin and levofloxacin, bedaquiline, linezolid, terizidone/cycloserine (pro-drug/active metabolite), and amikacin [10]. However, comprehensive TDM for second-line TB drugs is challenged by the chemical diversity of the drugs, and their diverse polarity complicates extraction and chromatographic separation. Therefore, TDM for second-line TB drugs is currently only performed by very few pharmacology laboratories, which solve this problem by re-grouping the drugs according to their hydrophobic/hydrophilic properties and analyzing the groups in separate HPLC–MS assays, either subsequently on the same instrument platform, or simultaneously on multiple platforms [12,13]. However, the set-up, maintenance, and utilization of multiple assays and/or instruments is resource-intensive and beyond the capacity of many laboratories.

Therefore, our aim was (i) to define a target profile for sufficient performance of a TDM assay for clinical application in drug-resistant TB, (ii) to develop and validate a comprehensive, single-run multiplex assay according to this target profile, and (iii) to show the applicability of the assay in the clinical care of drug-resistant TB patients.

## 2. Materials and Methods

### 2.1. The Definition of an Assay Target Profile

As a single-run multiplex assay would likely have to make significant compromises in its performance, we defined a target profile for sufficient and ideal performance of an HPLC–MS method for therapeutic drug monitoring in MDR/RR-, pre-XDR and XDR-TB (Table 1). Clinical recommendations and pharmacokinetic/pharmacodynamic target values as well as maximal concentrations (C_max_) were retrieved from the literature. The priority of drugs for TDM in MDR/RR-, pre-XDR and XDR-TB was rated according to clinical recommendations and prescription frequencies. Drugs were considered high priority for TDM in MDR/RR-, pre-XDR and XDRTB if drug monitoring was clinically recommended and the drugs were prescribed to ≥10% of our patients. Drugs were of medium priority if TDM was recommended OR the prescription frequency was ≥10%. Low-priority drugs were those for which TDM was not recommended AND the prescription frequency was <10%). We determined how often the drugs were prescribed as part of drug-resistant tuberculosis treatment regimens (patients with MDR/RR-TB, pre-XDR-TB and XDR-TB) at the Medical Clinic of the Research Center Borstel for three years after the introduction of the new WHO priority grouping of TB medicines (from August 2018 to August 2021), and in the same way determined the 100 most administered co-medications.

Sufficient sensitivity was defined by the ability to monitor drug concentrations over five half-lives from C_max_, at which point 97% of the drug is cleared from the body. For this, the lower end of the reported C_max_ range was divided by 32 to obtain the targeted lower limit of quantification (LLOQ_target_, Table 1). We aimed to cover concentrations up to two-fold C_max_. As high C_max_ would create solubility problems in the generation of stock solutions, calibrators, and quality controls, a 1:10 (*v*/*v*) pre-dilution step before extraction for clinical samples was included and the targeted upper limit of quantification (ULOQ_target_) for a sufficient calibration range was defined as the upper end of the C_max_ range divided by five. The LLOQ_target_ and ULOQ_target_ formed the targeted calibration range (Table 2). The low, medium, and high concentrations for quality control samples (QCs) were defined based on the LLOQ and ULOQ: QC_low_ at 3 * LLOQ_target_, QC_high_ at 0.75 * ULOQ_target_, and QC_med_ in between.

We aimed for validation according to the European Medicines Agency (EMA) and the Food and Drug Administration (FDA) guidelines on bioanalytical method validation and study sample analysis [14,15]. However, the assay was considered sufficiently valid, i.e., suitable for its intended purpose, if the expected deviation of measured concentrations from true concentrations allowed for clinical decision making. We aimed for low deviation of measured concentrations from true concentrations for the monitoring of drugs with threshold-defined toxicity (linezolid, amikacin), and for moderate deviation of measured concentrations from true concentrations for monitoring of efficacy when analytical results were combined with MIC data, as MIC data are determined only semi-quantitatively at exponential concentrations.

Antibiotics were combined in five panels (Table 2): Panel 1 was set up for clinical application and comprised all WHO group A, B, and C drugs except for streptomycin. Panels 2–5 resembled TB regimens before the new WHO grouping [26] and included kanamycin and capreomycin. Panel 2 included rifampicin, isoniazid, ethambutol, pyrazinamide, and streptomycin; panel 3: moxifloxacin, clofazimine, cycloserine, ethambutol, prothionamide, and capreomycin; panel 4: bedaquiline, linezolid, meropenem, clavulanic acid, and amikacin; and panel 5: rifabutin, levofloxacin, pretomanid, delamanid, PAS, and kanamycin.

### 2.2. Chemicals

Suppliers for HPLC-grade acetonitrile, HPLC-grade water, formic acid, antibiotics, stable isotope-labeled and non-labeled internal standards (IS) are listed in Supplementary Table S1. Stable isotope-labeled internal standards were available for all antibiotics except for pretomanid, clavulanic acid, streptomycin, and capreomycin, at the time of the development. Gentamicin [27] was used as non-labeled internal standard for streptomycin and capreomycin, stable isotope-labeled delamanid for pretomanid.

### 2.3. Sample Preparation

Antibiotics and internal standards were dissolved in water, acetonitrile, or 1:2 (volume of solute per total volume of solution, *v*/*v*) acetonitrile in water, to obtain stock solutions of 1 mg/mL. The extraction medium was produced by spiking 700 mL acetonitrile with stock solutions of stable isotope-labeled and non-labeled internal standards (Supplementary Table S1). Drug-free human EDTA plasma was collected from three female donors to be pooled subsequently, as well as from three female and three male donors to serve as individual donor plasma. Donor characteristics and clinical routine laboratory parameters are listed in Supplementary Table S2. Stock solutions, extraction media, and donor plasma were stored at −80 °C.

### 2.4. HPLC–MS/MS

An Agilent 1100 series HPLC (Agilent Technologies, Santa Clara, CA, USA) coupled to a Waters Micromass Quattro Premier XE triple quadrupole mass spectrometer was used for this study. Supplementary Table S3 summarizes the MS settings.

#### 2.4.1. MS/MS

The platform was operated in multi-reaction monitoring (MRM), using MassLynx 4.1 and TargetLynx (both Waters Corporation, Milford, MA, USA) for quantification. MRM channels were combined according to the antibiotic panels 1–5. Mass transitions and MRM channels for antibiotics, stable isotope-labeled and non-labeled internal standards were optimized following the instrument operation guide [28] (Table 3). Dwell times were 0.05 s to 0.1 s, except for amikacin, streptomycin, capreomycin, kanamycin which had dwell times of 0.5 s (Supplementary Figure S1). Inter-channel delay was 0.02 s. All compounds were detected in positive ion mode ([M + H]^+^ and [M + 2H]^2+^) except for clavulanic acid which was detected in negative ion mode ([M − H]^−^). The analyte peak area was normalized to the peak area of stable isotope-labeled internal standards. Where no specific stable isotope-labeled standards were available, other stable isotope-labeled or non-labeled internal standards were used: D-Delamanid was used for pretomanid, and gentamicin was used for streptomycin and capreomycin.

#### 2.4.2. HPLC

Four HPLC columns were tested, including an Atlantis^®^ dC18 and an HSS T3 (1 × 150 mm, 3.5 µm, 100 Å, and 2.1 × 150 mm, 3.5 µm, 100 Å, both Waters Corporation, Milford, MA, USA), as well as an Acclaim^®^ HILIC-1 mixed-mode (Thermo Fisher Scientific Inc., Waltham, MA, USA). The optimized set-up featured a zwitterionic hydrophilic interaction liquid chromatography column in the form of a SeQuant^®^ ZIC^®^-HILIC (2.1 × 150 mm, 5 µm, 200 Å, Merck Millipore, Burlington, MA, USA), with 1:100 formic acid in water (1%, *v*/*v*) as well as pure acetonitrile as solvents A and B. The total runtime was 30 min and the gradient is shown in Supplementary Table S4. The injection volume was 5 µL and the auto-sampler was cooled to 4 °C. We calculated the retention factor *k* and the asymmetry factor *A_S_*, as well as the column efficiency *N* [29] (Supplementary Tables S5 and S6).

### 2.5. Extraction Procedure

EDTA-anticoagulated whole blood was centrifugated at 1862× *g* for 15 min, 22 °C. The plasma was stored at ×80 °C until extraction using acetonitrile-based protein precipitation. For extraction, 100 µL of plasma samples were mixed with 700 µL of extraction medium on a shaker for 5 min at 1300 rpm, 22 °C. Subsequently, 100 µL of a 1% formic acid solution was added, followed by another 5 min of shaking at 1300 rpm, 22 °C. The samples were then centrifuged for 10 min at 15,000× *g*, 22 °C. Aliquots of the resulting supernatant (700 µL) were stored at −80 °C until analysis.

### 2.6. Validation

The assay was validated based on current EMA and FDA guidelines on bioanalytical method validation and study sample analysis and the pre-defined performance criteria. We prepared calibrators at ULOQ_target_ in plasma as well as quality control samples (QCs) at high, medium, and low concentrations (QC_high_, QC_med_, QC_low_) for antibiotic panels 1–5. For this, 1:2 (*v*/*v*) acetonitrile in water was spiked with antibiotic stock solutions to generate 10-fold concentrated stocks for every panel. Panel stocks were diluted 1:10 (*v*/*v*) in human plasma to generate ULOQ_target_ calibrators and QC_high_, QC_med_, QC_low_ samples.

#### 2.6.1. Calibration

Calibration curves were prepared by diluting the calibrators in pooled human plasma. We used calibrators at 13 concentration levels between ULOQ_target_ and 0.01 * ULOQ_target_, as well as blank matrix samples (without internal standards) and zero samples (matrix samples with internal standards). Calibrators were extracted in duplicates and analyzed. Calibration curves were calculated by 1/X^2^-weighed linear regression in GraphPad Prism (Version 10.0.0, GraphPad Software LLC, La Jolla, CA, USA). According to the EMA and FDA guidelines, the back-calculated concentration of each calibrator was required to be within ±20% of the nominal concentration at the LLOQ and within ±15% at all other concentration levels. No more than 50% of the replicates and/or 25% of the concentration levels were to be excluded.

#### 2.6.2. Carry-Over, Selectivity, and Specificity

Carry-over was determined from a blank matrix sample injected directly after the highest calibrator, calculated as % of the analyte’s response at the LLOQ and was required to not exceed 20% [14,15]. We assessed selectivity by injecting blank matrix samples before the first calibrator and specificity by comparing the selected mass spectrometry transitions of the analyte panel with the fragmentation mass spectra of the 100 most common co-medications, retrieved from two large chromatography databases [30,31,32,33].

#### 2.6.3. Recovery and Matrix Effect

Recovery and the matrix effect were calculated by dividing the peak area of samples that were spiked pre-extraction, by the peak area of samples that were spiked post-extraction (for recovery), or by the peak area of spiked solvents (for the matrix effect), respectively, according to the previous EMA and FDA guidelines [34,35]. QC_high_, QC_med_, and QC_low_ were prepared in the plasma of six individual donors each to serve as pre-extraction samples. For post-extraction and solvent samples, we spiked extracted plasma from six donors, as well as 1:2 (*v*/*v*) acetonitrile in water, with appropriately diluted panel stocks of panels 2–5 to obtain concentrations equivalent to QC_high_, QC_med_, and QC_low_ after extraction. Every sample was prepared and extracted in triplicate. We determined the mean recovery and matrix effect, as well as the percentage coefficient of variation (% CV), by dividing the standard deviations of recovery and the matrix effect by the respective means.

#### 2.6.4. Accuracy and Precision

Accuracy and precision were determined by a fast-track approach to achieve clinical applicability as early as possible, using all available data of QC samples from recovery and matrix effect experiments as well as QC samples from clinical application (internal validation). QC sample datasets were size-adjusted and included six samples per concentration level and measurement day. Accuracy, within- and between-day precision were calculated for each concentration level. Accuracy was determined as % nominal concentration, and within- and between-day precision were calculated using ANOVA [36]. We aimed for ±15% deviation according to EMA and FDA guidelines [14,15], i.e., accuracy between 85% and 115%, and within-day and between-day precision between 0% and 15% for each QC concentration level, QC_low_, QC_med_, and QC_high_. When QC sample sets showed systematic inaccuracy and/or imprecision, i.e., accuracy of <80% or >120% or precision of more than >20% in all measurements of an antibiotic, all available sample sets were sent for external analysis of the nominal concentration to the Laboratory of Clinical Pharmacology at Lausanne University Hospital, Lausanne, Switzerland. The results were defined as true nominal concentration, and accuracy, precision, and beta tolerance were calculated accordingly (external validation).

Whether the expected deviation of measured concentrations from true concentration was sufficiently low for clinical decision making was evaluated based on the 80% beta-expectation tolerance interval, i.e., the range of deviation in % from nominal concentrations in which 80% of future values are expected to fall. Like accuracy and precision, the interval was calculated based on all available QC sample sets. Calculations followed the recommendations of the German Society for Toxicology and Forensic Chemistry (GFTCh) [37,38] using an 80% interval margin [13]. The influence of the expected deviation on monitoring of toxicity was examined for linezolid and amikacin using 2 µg/mL as the toxicity threshold.

### 2.7. Clinical Application

The assay was applied to samples from two patients from the Medical Clinic of the Research Center Borstel, Borstel, Germany between March 2019 and February 2020.

#### 2.7.1. Patient Histories

Patient 1 had pre-XDR-TB with resistance to rifampicin, rifabutin, isoniazid, levofloxacin, moxifloxacin, ethambutol, delamanid, pyrazinamide, and prothionamide. Patient 2 was infected with an advanced-level XDR-TB with susceptibility only to delamanid and pretomanid and resistance to rifampicin, rifabutin, isoniazid, levofloxacin, moxifloxacin, bedaquiline, linezolid, clofazimine, ethambutol, pyrazinamide, meropenem, amikacin, streptomycin, prothionamide, and PAS. Both patients received high-dose therapy to overcome the minimal inhibitory concentration (MIC) of their *M. tuberculosis* strain. Patient 1 received moxifloxacin daily doses ≥800 mg and prolonged meropenem infusions. Patient 2 received moxifloxacin daily doses ≥800 mg, prolonged meropenem infusions and additional high-dose therapy with bedaquiline ≥250 mg three times per week, clofazimine 200 mg, and terizidone ≥1000 per day. The patients’ medical histories are described elsewhere, as both patients underwent routine TDM for their high-dose therapy in cooperation with our partner laboratories [13,39].

#### 2.7.2. Sample Collection and Management

Blood was drawn directly before, and at several timepoints after drug intake. After centrifugation of the whole blood, samples were diluted 1:10 (*v*/*v*) in pooled plasma. For routine TDM, samples were shipped on dry ice to the Department of Pharmacy at Radboud University Medical Center, Nijmegen, the Netherlands, for the analysis of moxifloxacin, bedaquiline, linezolid, clofazimine, and delamanid, as well as to the Department of Laboratory Medicine at Ghent University Hospital, Ghent, Belgium, for the analysis of meropenem and clavulanic acid, the Infectious Disease Pharmacokinetics Laboratory at University of Florida, Gainesville, FL, USA, and to the Laboratory of Clinical Pharmacology at Lausanne University Hospital, Lausanne, Switzerland, for the analysis of cycloserine. Patient 1 underwent one dose adjustment and subsequent drug concentration measurements, while patient 2 had a total of nine TDM cycles of drug concentration measurements and subsequent dose adjustments.

We co-analyzed one and three cycles for patients 1 and 2, respectively (TDM1/TDM1-3). For this, we extracted and analyzed every sample in triplicate. Calibration as well as QC samples used panel 1 to cover WHO groups A, B, and C drugs. If concentrations were below the calibration range, the measurement was repeated using undiluted samples. The patients’ sputum times to liquid culture positivity (TTP) were monitored as a surrogate for the therapy response and compared with TTP percentiles from a drug-resistant TB reference cohort [40]. The model-informed precision dosing application TDMx was used to calculate meropenem pharmacokinetic parameters from drug concentrations [41,42,43].

## 3. Results

### 3.1. Definition of an Assay Target Profile

Figure 1 shows the frequency of drugs prescribed as part of drug-resistant tuberculosis treatment regimens of *n* = 71 patients with MDR/RR-TB, pre-XDR-TB or XDR-TB who were treated at the Medical Clinic of the Research Center Borstel between August 2018 and August 2021. Group A and B drugs were most frequently prescribed, followed by meropenem/clavulanic acid (*n* = 21/71, 29.6%), delamanid (*n* = 15/71, 21.1%), prothionamide (*n* = 11/71, 15.5%), and pyrazinamide (*n* = 9/69, 12.7%). One patient received pretomanid even before the WHO recommended its use in 2022 and rifampicin was prescribed to five patients as high-dose therapy due to low-level rifampicin resistance (*n* = 5/71, 7.0%). In combination with the published TDM recommendations, levofloxacin, moxifloxacin, bedaquiline, and linezolid were considered to be drugs of high priority (+++) to be covered by a multiplex assay for TDM. Rifampicin, rifabutin, isoniazid, pretomanid (expected high frequency in the future), clofazimine, ethambutol, delamanid, meropenem/clavulanic acid, amikacin, and prothionamide were considered as drugs of medium priority (++), and PAS, streptomycin, capreomycin, and kanamycin as drugs of low priority (+) (Table 2).

### 3.2. HPLC-MS/MS

Table 3 summarizes mass transitions as well as observed retention times for a total of 50 antibiotics and internal standards.

#### 3.2.1. MS/MS

Fragmentation mass spectra of all compounds are displayed in Supplementary Table S7. Repetitive fragment signals in the fragmentation mass spectrum of moxifloxacin and stable isotope-labeled D-moxifloxacin resembled published fragmentation patterns [44]. Two out of four known capreomycin components were identified [45], IA and IB, detected as double charged ions. The pro-drug terizidone could not be ionized and was only detected as its active metabolite cycloserine. The MS needed several minutes to adequately switch from positive to negative ion mode, therefore clavulanic acid was not included in the further development.

#### 3.2.2. HPLC

**Figure 2:**
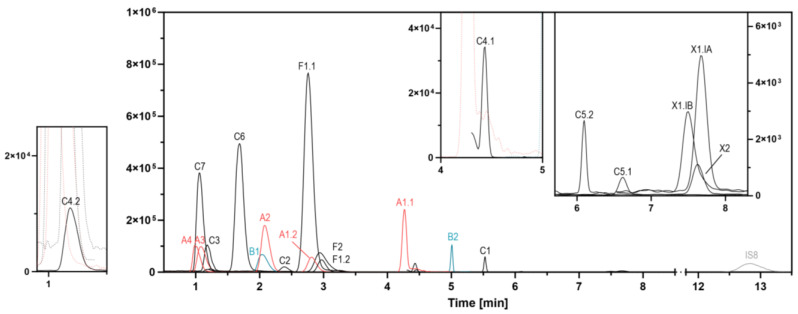
Chromatographic separation and peak intensity of all antibiotics. Antibiotics form integrable peaks and are sufficiently separated, especially within the WHO groups. WHO groups are color-coded—red: WHO group A (A1.1: levofloxacin; A1.2: moxifloxacin; A2: bedaquiline; A3: linezolid) as well as the unclassified drug pretomanid (A4); turquoise: WHO group B (B1: clofazimine; B2: cycloserine); black: first-line drugs (F1.1: rifampicin; F1.2: rifabutin; F2: isoniazid), WHO group C (C1: ethambutol; C2: delamanid, C3: pyrazinamide; C4.1: meropenem; C4.2: clavulanic acid; C5.1: amikacin; C5.2: streptomycin; C6: prothionamide; C7: PAS), and WHO-excluded drugs (X1.IB: capreomycin IB; X1.IA: capreomycin IA; X2: kanamycin); grey: non-labeled internal standards (IS8: gentamicin).

The SeQuant^®^ ZIC^®^-HILIC was the only column to yield integrable peaks for all analytes. Chromatographic separation and peak intensity are shown in Figure 2. Linezolid, pretomanid, delamanid, pyrazinamide, prothionamide, and PAS eluted early from the column (median retention factor <2, Supplementary Table S6), and levofloxacin, pretomanid, clofazimine, and PAS showed an asymmetric peak shape (asymmetry factor >2). The separation capacity, measured as column efficiency, was highest for cycloserine and lowest for linezolid. A total of 13 drugs co-eluted in four clusters: linezolid simultaneously with pretomanid, pyrazinamide, and PAS; bedaquiline together with clofazimine; moxifloxacin together with rifampicin, rifabutin, and isoniazid; and capreomycin IA and IB together with kanamycin. The maximum plausible duty cycle duration was 0.70 s with inactive channels for amikacin, streptomycin, capreomycin, kanamycin, and 1.04 s when either of them was activated (Supplementary Figure S1). The minimum number of data points per peak was nine in cycloserine. We observed retention time shifts and earlier elution with column aging for moxifloxacin, bedaquiline, and delamanid (Table 3) as well as peak tailing, especially for moxifloxacin, that limited column usage to 300 runs. Both the retention time shifts and the peak tailing were identical in the respective stable isotope-labeled internal standards (Supplementary Figure S2) and were compensated by prolonged MRM channel activation.

### 3.3. Validation

Validation parameters calibration, carry-over, recovery, and matrix effect are displayed in Table 4, calibration curves in Supplementary Table S8, selectivity in Supplementary Figure S3, specificity in Supplementary Table S9, and accuracy and precision in Table 5.

#### 3.3.1. Calibration

Calibration ranges for all antibiotics covered the targeted calibration range (Table 4 and see also Table 2 and Supplementary Table S9). Gentamicin was used as an internal standard for streptomycin and capreomycin. Using stable isotope-labeled internal standards, calibration weighted R^2^ surpassed 0.99 in all antibiotics except for amikacin (0.9835).

#### 3.3.2. Carry-Over, Selectivity, and Specificity

In the assessment of selectivity, co-eluting matrix peaks were below 20% of LLOQ (Supplementary Figure S3) and the carry-over was below 20% at LLOQ for all drugs, in accordance with the EMA and FDA guidelines [14,15]. In the specificity assessment, four commonly administered co-medications were found to show mass transitions that were in the range of ±1 Da of analyte transitions (Supplementary Table S9). Mesalazine had a similar transition to PAS (meta- vs. para-amino salicylic acid; relative intensity of the similar fragment: 1.0%), indomethacin was similar to pretomanid (5.8%), prednisolone and bisacodyl were similar to levofloxacin (3.6% and 10.5%, respectively).

#### 3.3.3. Recovery and Matrix Effect

One to four sample sets per concentration level went into the calculation of recovery and the matrix effect (Table 4). Recovery was between 47.7% for capreomycin IA and 105.6% for pyrazinamide, and the matrix effect ranged between 96.6% for bedaquiline and 231.7% for capreomycin IB. % CV of both recovery and the matrix effect were within the EMA/FDA-suggested range of 0–15% [34,46] for all antibiotics (delamanid matrix effect: 16.4% CV, kanamycin recovery: 15.3% CV), with larger deviations only in pretomanid, streptomycin, and capreomycin IB and IA (pretomanid matrix effect: 35.5% CV, streptomycin and capreomycin both parameters: 23.6–32.6%).

#### 3.3.4. Accuracy and Precision

The number of QC sample sets per concentration level that were used for the calculation, as well as the resulting accuracy and precision values and the 80% beta-expectation tolerance intervals, are displayed in Table 5. Three or more sample sets per concentration level were used for the calculation, except that for rifampicin, isoniazid, pyrazinamide, meropenem, and amikacin, only two QC sample sets were available (one in the case of amikacin). Rifampicin, isoniazid, levofloxacin, bedaquiline, linezolid, ethambutol, and pyrazinamide were validated externally, i.e., by comparing measured concentrations to externally determined nominal concentrations of the QC samples.

The evaluation of accuracy, within-day and between-day precision, as well as of the expected future deviation from the nominal concentration, indicated the following:

- Linezolid, delamanid, meropenem, and prothionamide were strictly within the EMA/FDA-recommended range of 85–115% accuracy and 0–15% within-day and between day precision [14,15]. The expected deviation from the nominal concentration in the form of the 80% beta-expectation tolerance interval was up to ±30% in meropenem and up to approximately ±40% in linezolid, delamanid, and prothionamide (linezolid: −19.3% to +40.4%, ethambutol: −9.7% to +41.0%, both at QC_low_).
- Moxifloxacin, clofazimine, cycloserine, and ethambutol showed accuracy of 80–120% and precision of approximately 0–20% (moxifloxacin between-day precision: 20.6%, ethambutol accuracy: 123.4%, both at QC_med_). 80% beta-expectation tolerance intervals were within approximately ±40% (moxifloxacin: −26.9% to +40.3% at QC_med_, ethambutol: −9.7% to +41.0% at QC_low_).
- Rifampicin, rifabutin, levofloxacin, bedaquiline, pyrazinamide, and PAS showed accuracy of approximately 80–120% and precision of approximately 0–20% with higher deviations at QC_low_: rifampicin with an accuracy of 122.2%, rifabutin with a between-day precision of 20.8%, and bedaquiline with a between-day precision of 24.4%. Correspondingly, rifampicin, rifabutin, levofloxacin, bedaquiline, pyrazinamide, and PAS showed higher expected deviations at low concentrations, with an 80% beta-expectation tolerance interval of up to ±60% at QC_low_ and ±30% at QC_med_ and QC_high_ for bedaquiline and pyrazinamide, as well as ±40% at QCmed and QChigh for rifampicin, rifabutin, levofloxacin, and PAS (PAS: −23.3% to +42.9% and −40.3% to +21.9% at QC_med_ and QC_high_, respectively).
- Amikacin was evaluated based on only one set of QC_low_, QC_med_, and QC_high_, hence stratified between-day precision could not be calculated and overall accuracy (93.5%), within-day (6.3%), and between-day precision (6.3%) were determined instead. The overall 80% beta-expectation tolerance interval was −15.2% to +2.2%.
- Isoniazid, pretomanid, streptomycin, capreomycin IB and IA, as well as kanamycin showed inadequate accuracy and/or within- and between-day precision. 80% beta-expectation tolerance intervals of isoniazid, pretomanid, streptomycin, capreomycin IB and IA, and kanamycin partly exceeded 100%. Quantification of isoniazid showed systematic deviations, with accuracies of 141.2% to 160.3%.

**Figure 3:**
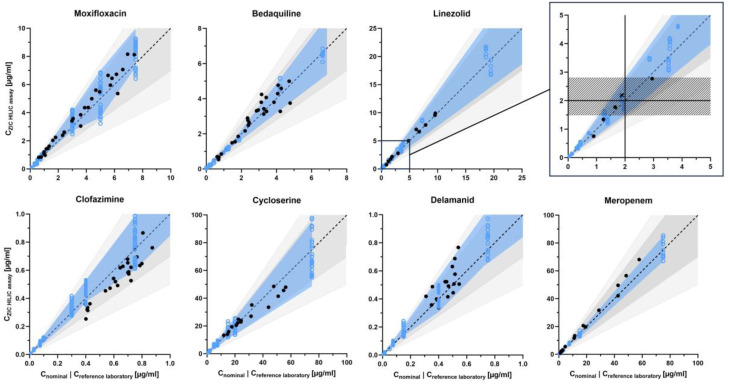
External control of measured drug concentrations of clinical samples and expected deviation from nominal concentration as 80% beta-expectation tolerance interval. Drug concentrations in clinical samples (black dots) mostly scatter within the 80%-beta tolerance intervals (blue area). The 80% beta tolerance intervals were mostly congruent with a tolerance interval of ±30% (dark grey area). No drug exceeded the ± 50% interval (light grey area). QC samples were plotted as actual concentration (blue dots) and extrapolated concentration after 1:10 dilution (blue circles). Detail frame: External control of measured drug concentrations of clinical samples and expected deviation from nominal concentration as 80% beta-expectation tolerance interval of linezolid around a toxicity threshold of 2 µg/mL. Between a measured concentration of 1.5 µg/mL and 2.8 µg/mL, the likelihood of misclassifying a patient as above or below the toxicity threshold of 2 µg/mL (black line) is >10% and measurements should be repeated (hatched area). One out of five displayed clinical samples were misclassified (symbol: X).

The 80% beta-expectation tolerance intervals of moxifloxacin, bedaquiline, linezolid, clofazimine, cycloserine, delamanid, and meropenem are also shown in Figure 3 together with the external control of drug concentrations of our clinical samples. Drug concentrations in clinical samples were mostly scattered within the beta intervals, with deviations in clofazimine concentrations. The comparison also indicated high accuracy and precision, only exceeding 15% deviation in delamanid (precision 16.4%, Table 5).

Linezolid accuracy in concentrations around the toxicity threshold of 2 µg/mL was high (Figure 3, detail frame). In five samples between 0.9 and 3.0 µg/mL accuracy was 101.7% with a precision of 8.4% CV. Only one out of four samples below 2 µg/mL was falsely classified as >2 µg/mL. The 80% beta-expectation tolerance interval at 2 µg/mL ranged from 1.5 µg/mL to 2.8 µg/mL. For amikacin, the non-stratified overall 80% beta-expectation tolerance interval at 2 µg/mL ranged from 1.7 µg/mL to 2.1 µg/mL.

### 3.4. Clinical Application

**Figure 4:**
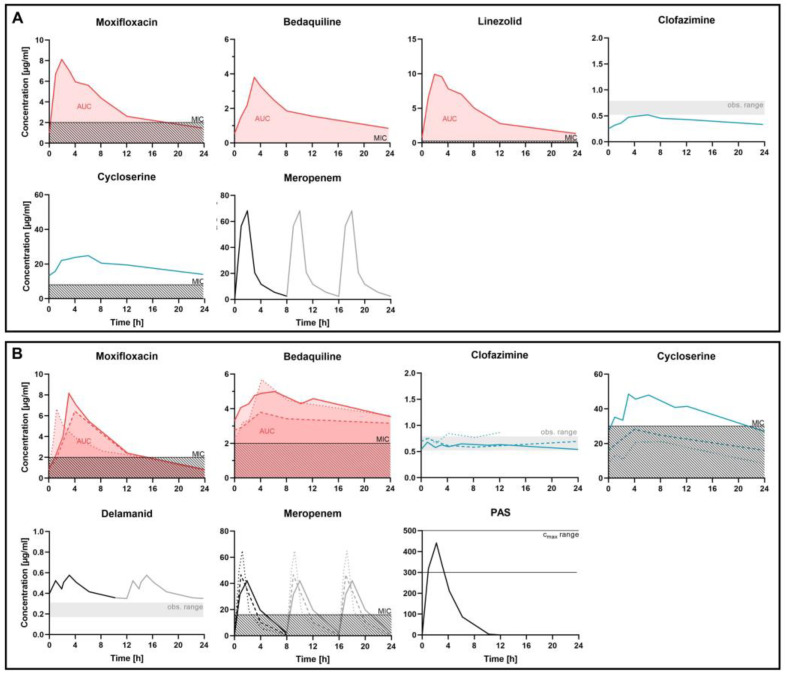
Pharmacokinetics of TDM cycle 1 in patient 1 (**A**) and TDM cycles 1-3 in patient 2 (**B**). All drugs included in their regimen could be quantified except for clavulanic acid. Moxifloxacin, bedaquiline, and linezolid are interpreted by area under the concentration–time curve (AUC, red area) divided by the minimal inhibitory concentration (MIC, hatched area), clofazimine, and delamanid by comparison to the normally observed concentration range (obs. range), meropenem by the time in which the concentration is above the MIC. Target parameters for intravenously administered PAS are unknown, therefore we compared the observed maximal concentration (Cmax) with the normally observed Cmax. Meropenem pharmacokinetics were measured once (black line) and extrapolated (grey line) to account for thrice daily administration. Dotted line: patient 2, TDM cycle 1; dashed line: patient 2, TDM cycle 2; continuous: patient 1, TDM cycle 1; patient 2, TDM cycle 3.

**Figure 5:**
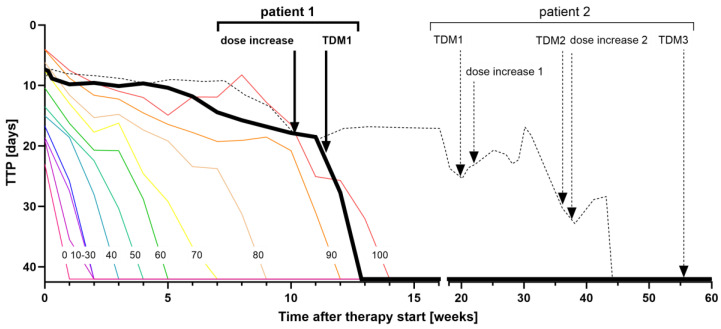
Therapy responses of patient 1 and patient 2 in relation to our drug-resistant TB reference cohort. Therapy response is displayed as the increase in time to liquid culture positivity (TTP) of sputum samples. Samples are considered negative after 42 days of culturing. TTP percentiles show the percentage of patients from our drug-resistant TB reference cohort [29], who are left of the percentile line, i.e., 50% of patients had their first negative sputum culture after four weeks or less, while the patient with the slowest response had theirs after 14 weeks (100th percentile). Patient 1 (black continuous line) improved quickly after dose increase, patient 2 (black dashed line) needed two TDM cycles with dose escalations to achieve negative cultures. The complete microbiology of patient 2 is shown in [39].

Figure 4 shows the pharmacokinetics of patient 1 (A) and patient 2 (B). The resulting TDM parameters for patient 1 T1 and patient 2 T3 are shown in Table 6 together with the TDM target values, the patients’ resistance and minimal inhibitory concentration testing, as well as the dosing regimens (see also [13,39]). Figure 5 compares the patients’ TTPs to the TTP percentiles of the reference cohort. Supplementary Figure S4 shows pharmacokinetic model-based calculation of meropenem. Both patients were slow to respond to therapy and had initial TTPs above the 100th percentile, i.e., a slower therapy response than the worst responding patient in the reference cohort.

After dose adjustment, patient 1 received 300% of the regular moxifloxacin dose (1200 mg instead of 400 mg daily), and a prolonged infusion of meropenem over two hours instead of one hour (Table 6). Every antibiotic in both regimens could be quantified. TDM indicated effective exposure to bedaquiline, linezolid, and cycloserine as well as to meropenem up to a minimal inhibitory concentration of 8 µg/mL. Clofazimine concentrations were below the regular range (0.25–0.52 µg/mL, regular range 0.52–0.79 g/mL) and the moxifloxacin exposure below the effectivity target (*f*AUC/MIC = 21.26, target: >53). After dose adjustment, *M. tuberculosis* growth in liquid culture declined rapidly and the patient crossed the 0th percentile (Figure 5). After three weeks, the patient achieved culture conversion (between 0th and 10th percentile), i.e., no growth could be detected anymore.

Patient 2 achieved culture conversion only after two TDM cycles and 44 weeks of treatment. At this point, he received 400% of the regular moxifloxacin dose (1600 mg per day instead of 400 mg), 150% of bedaquiline (300 mg instead of 200 mg thrice weekly), 200% of clofazimine (200 mg instead of 100 mg daily), and 133% of the regular cycloserine dose (administered as the pro-drug terizidone, 1000 mg instead of 750 mg terizidone daily) (Table 6). TDM3 indicated under-dosing of moxifloxacin, bedaquiline, and meropenem, regular concentrations of clofazimine and PAS as well as sufficient exposure to cycloserine, and high concentrations of delamanid. Under this regimen, *M. tuberculosis* was undetectable in weekly cultures from sputum samples over 8 months. However, later in treatment, the patient had two relapses and ultimately died due to Gram-negative bacterial sepsis [39].

## 4. Discussion

We developed a TDM multiplex assay for HPLC–MS/MS that allows quantification of first- and second-line anti-TB medicines from patient plasma in a single run. The assay performance was assessed and validated, and the assay was successfully applied in clinical practice. In the assay development process, we set a clear clinical focus and defined target profiles for analytical and clinical performance in an international multi-professional team of clinicians, pharmacologists, pharmacists, microbiologists, and analytical chemists.

The sensitivity and range of our assay fulfilled our pre-defined requirements of sufficient performance. All analytes could be calibrated from the targeted LLOQ to the targeted ULOQ. The a priori dilution of clinical samples is not common practice, but allowed us to combine the WHO group A, B, and C drugs in one analyte panel and enabled a calibration range equivalent to a 1:100 (*v*/*v*)-dilution (e.g., 1 µg/mL to 0.01 µg/mL or 10 µg/mL to 0.1 µg/mL) to cover the complete expected concentration range in clinical samples. Other assays used undiluted samples and featured a calibration range between a 1:20 (*v*/*v*)-, or a 1:50 (*v*/*v*)-dilution [12,27] and a 1:500 (*v*/*v*)-dilution [13]. However, these assays usually grouped the drugs into several analyte panels and/or featured fewer drugs per panel. Higher ULOQs and narrower calibration ranges [12,27] might also come at the expense of reduced sensitivity.

We could show EMA/FDA-compliant calibration, recovery, matrix effect, carry-over, and selectivity for all evaluated antibiotics, as well as accuracy and precision for linezolid, delamanid, meropenem, and prothionamide. The accuracy and precision of levofloxacin, moxifloxacin, bedaquiline, clofazimine, cycloserine, ethambutol, pyrazinamide, and PAS, did not comply strictly with the EMA/FDA guidelines but showed sufficiently low expected deviation of measured concentrations from true concentrations to support clinical decision making, with 80% beta-expectation tolerance intervals of approximately ±40%. Expected deviations of up to 60% only occurred at QC_low_ of rifampicin, rifabutin, levofloxacin, bedaquiline, pyrazinamide, and PAS, but low concentration ranges contribute little to the total drug exposure and hence have only a low impact on the *f*AUC/MIC-driven monitoring of efficacy in these drugs (the *f*C_min_ target of PAS only applies to peroral administration). External control of measured drug concentrations indicated high accuracy and precision in clinical samples of moxifloxacin, bedaquiline, linezolid, clofazimine, cycloserine, delamanid, and meropenem.

In clinical application, the expected deviation from the actual concentration should, in any case, be reported in the form of the 80%-beta tolerance interval. A narrow tolerance interval and high accuracy and precision were especially important for linezolid. Above a trough concentration C_min_ of 2–2.5 µg/mL, the risk of linezolid-driven toxicity doubles with each 1-µg/mL-increase in C_min_ and patients with C_min_ of 2–4 µg/mL have a significantly higher risk of toxicity than patients with C_min_ < 2 µg/mL [5,47]. The measured concentration range of our assay, in which C_min_ would be misclassified with a likelihood of <10%, was below a measured C_min_ of 1.5 µg/mL and above a measured C_min_ of 2.8 µg/mL. Between these concentration levels, classification would be unclear, and the measurement should be repeated. Based on published C_min_ distributions [5], our assay could classify three out of four patients safely and one out of four would need repeat measurements. In clinical application, linezolid accuracy and precision around the toxicity threshold were very high, and only one out of five samples were misclassified. For amikacin, the measured concentration range in which C_min_ could not be misclassified safely was relatively narrow (between 1.7 µg/mL and 2.1 µg/mL). Trough concentrations > 2 µg/mL increased the risk of nephrotoxicity approximately five-fold in a recent meta-analysis of non-TB patients [48]. Amikacin pharmacokinetics are mainly dependent on renal function and trough concentrations vary significantly between different patient populations [49,50]. Hence, it could not be predicted how many patients would have to undergo repeated measurements. The frequency of amikacin prescription at our medical center was very low and our validation dataset did not include sufficient data to calculate stratified amikacin parameters, so the calculated overall amikacin parameters need to be interpreted with caution.

Overall, we considered this assay sufficiently sensitive, accurate and precise to support clinical decision making in the toxicity monitoring of linezolid and the monitoring of efficacy in rifampicin, rifabutin, all WHO group A and B drugs, as well as group C drugs ethambutol, delamanid, pyrazinamide, meropenem, prothionamide and PAS, hence all drugs that were identified as high priority and six out of ten drugs of medium priority. This broad analyte panel was covered in a single-run multiplex assay. There is a similar single-instrument assay that comprises moxifloxacin and levofloxacin as well as linezolid and cycloserine, but no bedaquiline, clofazimine, and delamanid [27]. Similarly comprehensive or even more comprehensive assays rely on more than one analytical run [12,13].

Our method description has several limitations. We chose a fast-track approach for determining accuracy and precision, in order to achieve clinical applicability as early as possible. For this, we used datasets of samples from recovery and matrix effect experiments that were originally not generated for the evaluation of accuracy and precision and might have led to drugs not meeting the EMA/FDA recommendations for these parameters. Yet in case of systematic deviations, nominal concentrations of QC samples could be established externally and confirmed sufficient accuracy and precision of the assay. The assay performance was also not evaluated at LLOQ, but QC_low_ was chosen as low enough to show sufficient performance around the toxicity thresholds of linezolid and amikacin, and the performance at LLOQ again had little impact on the *f*AUC/MIC-based monitoring of efficacy. Overall, the results from the fast-track approach indicated sufficient performance to support clinical decision making, so we did not repeat the validation procedure according to EMA/FDA guidelines.

Isoniazid, pretomanid, clavulanic acid, streptomycin, kanamycin, and capreomycin could not be successfully validated. However, isoniazid, streptomycin, capreomycin and kanamycin played little to no role in the clinical management of our patients with drug-resistant TB. Pretomanid is a cornerstone of the new BPaLM regimen [3], which most likely failed validation because there was no stable isotope-labeled internal standard available when our experiments started. Yet, it has become available by now [51] and future assay developments should aim to include pretomanid as well.

Furthermore, we chose a ZIC^®^-HILIC column that was designed for separating hydrophilic substances to include cycloserine in our single-run setup. As a result, hydrophobic analytes eluted early and were incompletely separated so that antibiotics had to be quantified simultaneously. Yet, with a minimum of eight data points per peak, sufficient data resolution was maintained [52]. Observed retention-time shifts, asymmetric peak shapes, and peak tailing did not compromise the assay’s ability to sufficiently support clinical decision making. The throughput of the assay was medium to low. It was mainly limited by the long runtime of 30 min, whereas other assays only take 9–13, 5–7, and 3 min [12,13,27]. Shorter runtimes could possibly be achieved by shortening the washing step or by omitting capreomycin, kanamycin, and gentamicin from the panel, but throughput was sufficient for our setting so we did not further optimize it.

Finally, the assessment of specificity indicated a potential signal interference from mesalazine, indomethacin, bisacodyl, and prednisolone, the active metabolite of prednisone. The relative intensity of the potentially interfering fragments was low, as the drugs have a short half-life and are administered daily [53,54,55,56], so the drugs can be paused 24 h before drug monitoring so as to rule out interference.

The assay was successfully applied for TDM in one patient with pre-XDR-TB and one patient with XDR-TB based on individual physicians’ decisions. Both patients were slow to respond to therapy and harbored *M. tuberculosis* strains with low-level drug resistances. Low-level phenotypic drug resistances were overcome by applying higher doses of medicines, and in both patients *M. tuberculosis* load became undetectable following one and two TDM cycles with dose adjustments, respectively. Although there is no definitive proof, the treatment histories strongly suggest an association between the application of TDM and the microbiological response [13,39], indicating that the assay could make a difference in the clinical management of affected patients.

## 5. Conclusions

We successfully developed a single-run multiplex assay on a single-instrument HPLC–MS platform which enables TDM of the relevant first- and second-line antibiotics rifampicin, rifabutin, levofloxacin, moxifloxacin, bedaquiline, clofazimine, terizidone/cycloserine, delamanid, meropenem, pyrazinamide, prothionamide, and PAS. The assay performance was sufficient for the intended clinical application in therapeutic drug monitoring for patients with drug-resistant TB. Clinical applicability of the assay was demonstrated in two patients with advanced-level drug-resistant tuberculosis.

## Figures and Tables

**Table 1:** Sufficient and ideal performance criteria for a multiplex assay for TDM in MDR/RR-, pre-XDR- and XDR-TB.

Criterion	Sufficient and Ideal Performance
Analyte panel	High priority (+++): TDM recommended [10] AND high prescription frequency (≥10%, Figure 1) Medium priority (++): TDM recommended [10] OR high prescription frequency Low priority (+): TDM not recommended [10] AND low prescription frequency
Platform	Ideal: single run/single instrument assay Sufficient: single run/multi-instrument assay or multi-run/single instrument assay
Throughput	Ideal: high Sufficient: low—medium
Sensitivity	LLOQ: cover pharmacokinetics from lower C_max_ over five half-lives: ${LLOQ}_{target}\leq {low\;C_{max}*\left(\frac{1}{2}\right)}^{5}={low\;C}_{max}*\frac{1}{32}$ ULOQ: Ideal: cover concentrations up to two-fold of higher C_max_: ${ULOQ}_{target}\geq {high\;C}_{max}*2$ Sufficient: High-conc. samples are diluted 10-fold in plasma prior to extraction. ${ULOQ}_{target}\geq {high\;C}_{max}*2*\frac{1}{10}={high\;C}_{max}*\frac{1}{5}$
Validation	Ideal: strict validation according to FDA [14], EMA/CHMP/ICH [15], and proficiency testing [16] Sufficient: capacity to support clinical decision making; Low expected deviation from true concentration for the monitoring of toxicity in drugs with a defined threshold; Moderate expected deviation from true concentration for the monitoring of efficacy

TDM: therapeutic drug monitoring; C_max_: maximal or peak concentration; LLOQ: lower limit of quantification; ULOQ: upper limit of quantification; FDA: Food and Drug Administration; EMA/CHMP/ICH: European Medicines Agency/Committee for Medicinal Products for Human Use/International Council for Harmonisation of Technical Requirements for Pharmaceuticals for Human Use.

**Table 2:** Published pharmacokinetic and pharmacodynamic targets for TDM of anti-TB drugs, derived sufficient and targeted calibration range and QC concentrations.

WHO Group	Analyte	Panel	TDM Recommended [10]	TDM Priority	Toxicity Target [10] [µg/mL]	Efficacy Target [10]	C_max_ [10] [µg/mL]	Target Calibration LLOQ_target_-ULOQ_target_ [µg/mL]	QC_low_ [µg/mL]	QC_med_ [µg/mL]	QC_high_ [µg/mL]
First-line	rifampicin	2	toxicity/efficacy	++	-	AUC/MIC > 271	8–24	0.2–10	0.6	1.5	7.5
	rifabutin	5	toxicity [17]	++	-	-	0.45–0.9 [18]	0.01–0.5	0.03	0.075	0.4
	isoniazid	2	toxicity/efficacy	++	-	AUC/MIC > 567	3–6	0.1–5	0.3	0.75	3.75
Group A	levofloxacin	1, 5	efficacy	+++	-	AUC/MIC > 146	8–13	0.25–2.5	0.75	1.25	2
	moxifloxacin	1, 3	efficacy	+++	-	*f*AUC/MIC > 53	3–5	0.1–1	0.3	0.5	0.75
	bedaquiline	1, 4	toxicity/efficacy [19]	+++	-	AUC/MIC > 74.6 * [19]	0.9–4.3	0.01–1	0.03	0.15	0.75
	linezolid	1, 4	toxicity/efficacy [20]	+++	C_min_ > 2–2.5	*f*AUC/MIC > 125 [20]	12–26	0.1–5	0.3	0.75	4
Unclassified	pretomanid	5	*-*	++	-	*-*	1.4–4.3 [21]	0.01–1	0.03	0.15	0.75
Group B	clofazimine	1, 3	*-*	++	-	-	0.5–2	0.01–0.5	0.03	0.075	0.4
	cycloserine	1, 3	toxicity/efficacy	+++	-	%T > MIC > 30%	20–35	0.5–10	1.5	2	7.5
Group C	ethambutol	1, 2, 3	toxicity	++	-	AUC/MIC > 119	2–6	0.062–2.5	0.18	0.4	1.9
	delamanid	1, 5	*-*	++	-	*-*	0.3–0.9 [22]	0.005–0.5	0.015	0.075	0.4
	pyrazinamide	1, 2	toxicity/efficacy	+++	-	AUC/MIC > 8.42	20–60	0.5–10	1.5	2	7.5
	meropenem	1, 4	*-*	++	-	%T > MIC > 60% [22]	50–100 [23]	0.1–10	0.3	1.5	7.5
	clavulanic acid	1, 4	*-*	++	-	*-*	2.5–4 [24]	1–10	1.5	2	7.5
	amikacin	1, 4	toxicity/efficacy	++	C_min_ > 2	C_max_/MIC > 75	35–45	1–10	3	5	7.5
	streptomycin	2, 3	*-*	+	-	*f*C_max_/MIC > 20 [25]	35–45 [25]	1–10	3	5	7.5
	prothionamide	1, 3	*-*	++	-	AUC/MIC > 56.2	2–5	0.05–1	0.15	0.2	0.75
	PAS	1, 5	*-*	+	-	*f*C_min_ > 1	20–60	0.5–10	1.5	2	7.5
Excluded	capreomycin	3	*-*	+	-	*f*C_max_/MIC > 20 [25]	35–45 [25]	1–10	3	5	7.5
	kanamycin	5	*-*	+	-	*f*C_max_/MIC > 20 [25]	35–45 [25]	1–10	3	5	7.5

C_max_: maximal or peak concentration; LLOQ: lower limit of quantification; ULOQ: upper limit of quantification; QC_low_, QC_med_, QC_high_: quality control standard for low, medium, and high concentrations; *f*AUC: (unbound/free) Area under the concentration-time curve; MIC: minimal inhibitory concentration; *f*C_min_: (unbound/free) minimal or trough concentration; %T > MIC: percent time in which the concentration exceeds the MIC; +++: high priority for TDM; ++: medium priority for TDM; +: low priority for TDM; * AUC/MIC > 175.5 had a higher probability of culture conversion after 2-month treatment, AUC/MIC > 118.2 had a higher probability of culture conversion after 6-month treatment, AUC/MIC > 74.6 had a higher probability of successful treatment outcome.

**Table 3:** Mass transitions, MS settings, retention times, and MRM channel settings for 50 antibiotics and internal standards.

WHO Group	Analyte	Precursor [*m*/*z*]	→	Fragment [*m*/*z*]	Ion Mode	Cone Voltage [eV]	Collision Energy [eV]	Retention Time [min] Median (90% RT Range)	Channel Start [min]	Channel Stop [min]	Dwell [s]
First-line	rifampicin	823.46	→	791.01	[M + H]^+^	30	20	2.60 (2.43–2.89)	1.75	4.5	0.05
	D-rifampicin	830.25	→	798.69	[M + H]^+^	30	20	2.61 (2.44–2.89)	1.75	4.5	0.05
	rifabutin	847.02	→	815.52	[M + H]^+^	40	35	2.91 (2.79–3.61)	1.75	3.75	0.05
	D-rifabutin	852.59	→	821.16	[M + H]^+^	40	35	2.92 (2.79–3.62)	1.75	3.75	0.05
	isoniazid	137.68	→	78.50	[M + H]^+^	25	25	2.81 (2.70–3.40)	2	4.5	0.10
	D-isoniazid	141.72	→	82.60	[M + H]^+^	25	25	2.89 (2.78–3.47)	2	4.5	0.10
Group A	levofloxacin	361.82	→	261.17	[M + H]^+^	40	30	4.25 (4.08–4.51)	2	5	0.05
	D-levofloxacin	366.04	→	261.04	[M + H]^+^	40	30	4.25 (3.63–4.42)	2	5	0.05
	moxifloxacin	401.67	→	364.21	[M + H]^+^	40	35	3.18 (2.77–4.20)	2	4.8	0.05
	D-moxifloxacin	407.30	→	369.08	[M + H]^+^	40	35	3.20 (2.77–4.20)	2	4.8	0.05
	bedaquiline	555.14	→	58.16	[M + H]^+^	30	35	2.52 (2.08–3.15)	1.5	4	0.05
	D-bedaquiline	561.13	→	64.13	[M + H]^+^	30	35	2.52 (2.08–3.16)	1.5	4	0.05
	linezolid	337.17	→	195.20	[M + H]^+^	40	25	1.06 (0.96–1.10)	0.5	1.5	0.05
	D-linezolid	345.10	→	203.20	[M + H]^+^	40	25	1.07 (1.03–1.10)	0.5	1.5	0.05
Un-clssfd	pretomanid	359.00	→	174.82	[M + H]^+^	35	25	1.00 (0.95–1.05)	0	2	0.05
Group B	clofazimine	471.61	→	395.25	[M + H]^+^	60	45	2.41 (2.02–2.88)	1.5	4	0.05
	D-clofazimine	480.00	→	396.15	[M + H]^+^	60	45	2.29 (2.02–2.87)	1.5	4	0.05
	cycloserine	102.88	→	75.03	[M + H]^+^	25	10	4.99 (4.92–5.10)	4.6	5.3	0.05
	D-cycloserine	106.22	→	78.85	[M + H]^+^	25	10	4.99 (4.92–5.10)	4.6	5.3	0.05
Group C	ethambutol	204.78	→	44.29	[M + H]^+^	20	25	5.48 (5.45–5.62)	5.2	6	0.05
	D-ethambutol	208.78	→	48.29	[M + H]^+^	20	25	5.50 (5.45–5.67)	5.2	6	0.05
	delamanid	534.37	→	352.07	[M + H]^+^	35	25	1.87 (1.70–2.51)	1.2	3.3	0.05
	D-delamanid	538.30	→	356.07	[M + H]^+^	35	25	1.95 (1.73–2.62)	1.2	3.3	0.05
	pyrazinamide	123.68	→	78.88	[M + H]^+^	20	20	1.19 (1.16–1.21)	0	2	0.05
	D-pyrazinamide	127.21	→	82.85	[M + H]^+^	20	20	1.19 (1.09–1.21)	0	2	0.05
	meropenem	383.95	→	68.04	[M + H]^+^	25	30	4.58 (4.43–4.68)	4	5	0.05
	D-meropenem	389.96	→	68.06	[M + H]^+^	25	30	4.59 (4.44–4.68)	4	5	0.05
	clavulanic acid *	198.00	→	136.00	[M − H]^−^	20	10	1.17 *	0	4	0.10
	amikacin	586.06	→	163.07	[M + H]^+^	30	30	6.94 (6.62–7.29)	6	8	0.50
	D-amikacin	590.11	→	162.52	[M + H]^+^	30	30	6.93 (6.60–7.28)	6	8	0.50
	streptomycin	582.03	→	263.10	[M + H]^+^	65	35	6.12 (6.07–6.35)	5.7	6.8	0.50
	prothionamide	180.57	→	120.94	[M + H]^+^	35	25	1.76 (1.65–2.25)	1.2	3	0.05
	D-prothionamide	187.58	→	127.38	[M + H]^+^	35	25	1.79 (1.70–2.31)	1.2	3	0.05
	PAS	153.64	→	91.17	[M + H]^+^	25	25	1.09 (0.98–1.21)	0.7	1.4	0.05
	D-PAS	159.94	→	96.09	[M + H]^+^	25	25	1.07 (1.04–1.12)	0.7	1.4	0.05
Excluded	capreomycin IB ^§^	326.21	→	70.18	[M + 2H]^2+^	25	20	7.60 (7.45–8.70)	6.5	10	0.50
	capreomycin IA ^§^	334.27	→	70.24	[M + 2H]^2+^	25	20	7.79 (7.61–8.97)	6.5	10	0.50
	kanamycin	484.46	→	162.48	[M + H]^+^	35	25	7.55 (7.46–8.27)	5	9	0.50
	D-kanamycin	490.63	→	162.52	[M + H]^+^	35	25	7.31 (7.24–7.95)	5	9	0.50
	gentamicin	477.43	→	157.15	[M + H]^+^	30	20	12.07 (10.21–13.81)	9	22	0.50

*m*/*z*: mass to charge ratio; precursor/fragment: mass to charge ratio of an ionized molecule before (precursor) and after (fragment) fragmentation in the collision cell of a triple quadrupole mass spectrometer; eV: electron volt; ES+/ES−: positive/negative ion mode, ionization by adding positively charged protons/negatively charged electrons; Unclssfd: unclassified; * clavulanic acid could not be quantified together with the other analytes as our mass spectrometer took too long to switch from positive to negative ion mode, and therefore had only scarce data on retention time. ^§^ capreomycin IB and IA are two out of four cyclic peptides with antimicrobial activity that are contained in the drug preparation of capreomycin.

**Table 4:** Validation parameters calibration, carry-over, recovery, and matrix effect.

WHO Group	Analyte	Calibration Range [µg/mL]	R^2^	Carry-Over [% LLOQ]	Sample Sets n_low_; n_med_; n_high_	Recovery [%] (%CV)	Matrix Effect [%] (%CV)
First-line	rifampicin	0.1–10	0.9935	1.3	1; 1; 1	102.6 (7.5)	135.4 (13.1)
	rifabutin	0.005–0.5	0.9952	2.4	2; 2; 2	96.9 (6.1)	106.3 (6.4)
	isoniazid	0.05–5	0.9951	0.0	1; 1; 1	101.2 (6.7)	101.3 (3.9)
Group A	levofloxacin	0.025–2.5	0.9947	6.3	2; 2; 2	98.0 (4.2)	98.2 (7.8)
	moxifloxacin	0.01–1	0.9916	0.0	3; 3; 4	96.2 (7.6)	107.3 (5.1)
	bedaquiline	0.01–1	0.9969	0.6	1; 1; 1	86.2 (5.5)	96.6 (4.6)
	linezolid	0.05–5	0.9968	4.1	1; 1; 1	93.3 (6.0)	96.9 (4.9)
Unclassified	pretomanid *	0.015–1	0.9902	0.0	2; 2; 2	97.2 (13.4)	106.7 (35.5)
Group B	clofazimine	0.005–0.5	0.9974	0.0	3; 3; 4	96.1 (7.7)	102.1 (3.9)
	cycloserine	0.1–10	0.9914	0.0	3; 3; 4	94.6 (10.3)	116.2 (4.8)
Group C	ethambutol	0.025–2.5	0.9919	8.4	1; 1; 1	91.8 (9.2)	103.6 (6.6)
	delamanid	0.005–0.5	0.9959	0.0	3; 3; 3	83.0 (9.8)	101.1 (16.4)
	pyrazinamide	0.1–10	0.9953	19.0	1; 1; 1	105.6 (6.7)	97.2 (3.3)
	meropenem	0.1–10	0.9916	6.7	1; 1; 1	76.1 (10.3)	105.9 (6.1)
	clavulanic acid	–	-	-	-	-	-
	amikacin	0.1–10	0.9835	0.0	1; 1; 1	47.8 (13.3)	139.8 (11.3)
	Streptomycin ^$^	0.2–10	0.9913	0.0	1; 1; 1	56.3 (28.1)	183.6 (35.8)
	prothionamide	0.01–1	0.9974	1.0	3; 3; 4	97.0 (7.8)	98.9 (12.7)
	PAS	0.1–10	0.9957	1.2	2; 2; 2	95.8 (4.0)	100.7 (7.3)
excluded	capreomycin IB ^$^	0.2–10	0.9903	0.0	3; 3; 4	49.9 (24.1)	231.7 (32.6)
	capreomycin IA ^$^	0.1–10	0.9879	0.0	3; 3; 4	47.7 (23.6)	206.5 (30.2)
	kanamycin	0.25–10	0.9910	16.7	2; 2; 2	63.9 (15.3)	153.5 (12.2)

Carry-over was calculated as % of the peak area at the LLOQ. Each sample set comprised six samples measured in three replicates. The number of sample sets that went into calculation of recovery and matrix effect is listed under sample sets. R^2^: weighted correlation coefficient; % CV: percent coefficient of variation; * normalized to D-delamanid; ^$^ normalized to gentamicin.

**Table 5:** Validation parameters accuracy and precision as well as 80%-beta tolerance interval.

WHO Group	Analyte	Sample Sets n_low_; n_med_; n_high_	Val.	Accuracy [%] (wthn−Day Prec. [%]; btw−Day prec. [%])			80 % Beta−Expectation Tolerance Interval [%]			Accuracy [%] (Precision [%CV])
				QC_low_	QC_med_	QC_high_	QC_low_	QC_med_	QC_high_	Patient Samples
First−line	rifampicin	2; 2; 2	ext	122.2 (1.9; 9.7)	113.0 (3.4; 5.6)	114.5 (2.4; 1.5)	−14.9; +59.4	−10.6; +36.7	+9.9; +19.2	-
	rifabutin	3; 3; 2	int	103.8 (2.1; 20.8)	103.0 (3.5; 14.4)	102.1 (3.3; 3.4)	−41.7; +49.3	−29.0; +35.1	−8.1; +12.3	-
	isoniazid	2; 2; 2	ext	160.3 (2.2; 14.8)	141.2 (1.0; 0.7)	146.9 (4.6; 7.6)	+4.2; +116.4	+39.1; +43.3	+14.8; +78.9	-
Group A	levofloxacin	3; 3; 3	ext	95.7 (5.8; 17.8)	97.1 (6.8; 14.4)	105.0 (4.5; 13.4)	−44.7; +36.1	−36.9; +31.1	−25.5; +35.5	-
	moxifloxacin	5; 6; 6	int	113.8 (9.0; 12.9)	106.7 (4.9; 20.6)	114.9 (4.6; 11.0)	−10.0; +37.6	−26.9; +40.3	−3.5; +33.2	113.6 (7.5)
	bedaquiline	3; 3; 3	ext	97.2 (6.2; 24.4)	94.3 (3.5; 3.9)	102.0 (4.9; 10.1)	−57.2; +51.7	−14.5; +3.1	−21.9; +25.9	106.5 (11.2)
	linezolid	3; 3; 3	ext	110.5 (5.3; 12.9)	108.3 (3.5; 12.9)	106.3 (3.6; 14.2)	−19.3; +40.4	−20.5; +37.1	−25.5; +38.1	102.6 (8.4)
Unclassified	pretomanid *	3; 3; 3	int	91.5 (20.0; 37.6)	96.1 (19.3; 29.2)	96.7 (13.3; 38.3)	−99.1; +82.1	−67.9; +60.0	−90.6; +84.1	-
Group B	clofazimine	5; 6; 6	int	108.6 (8.2; 13.1)	106.2 (3.5; 15.8)	113.2 (3.4; 11.7)	−15.3; +32.5	−19.6; +32.0	−6.1; +32.5	90.1 (9.6)
	cycloserine	4; 5; 5	int	103.3 (14.7; 11.9)	97.7 (6.7; 17.3)	95.9 (8.4; 17.6)	−24.2; +30.8	−32.0; +27.4	−35.2; +27.1	105.7 (11.7)
Group C	ethambutol	2; 5; 5	int	115.6 (7.7; 8.8)	123.4 (5.0; 8.1)	114.1 (4.8; 10.3)	−9.7; +41.0	+8.7; +38.2	−4.0; +32.3	-
	delamanid	3; 4; 3	int	114.0 (5.0; 9.4)	113.0 (4.4; 13.5)	107.4 (6.1; 13.2)	−8.6; +36.6	−12.8; +38.7	−23.6; +38.4	113.6 (16.4)
	pyrazinamide	2; 2; 2	ext	116.1 (2.0; 9.5)	108.1 (1.1; 4.1)	113.6 (2.8; 2.2)	−20.5; +52.7	−7.9; +24.0	+7.0; +20.1	−
	meropenem	2; 2; 2	int	101.5 (6.7; 1.7)	95.4 (7.1; 4.7)	101.1 (8.5; 0.0)	−8.6; +11.6	−18.9; +9.8	−11.1; +13.4	92.3 (10.8)
	clavulanic acid	-	-	-	-	-	-	-	-	
	amikacin	1; 1; 1	int		93.5 (6.3; 6.3) ^§^			−15.2; +2.2 ^§^		-
	streptomycin ^$^	3; 4; 4	int	131.8 (19.0; 13.0)	106.1 (16.3; 60.2)	82.5 (23.6; 37.3)	−2.5; +66.1	−107.5; +119.7	−88.5; +53.5	-
	prothionamide	3; 4; 3	int	113.6 (10.2; 6.1)	111.1 (5.5; 11.5)	111.2 (10.4; 6.2)	−3.7; +31.0	−10.3; +32.5	−6.5; +28.9	-
	PAS	3; 3; 3	int	109.0 (2.5; 16.4)	109.8 (1.9; 15.1)	90.8 (2.5; 14.1)	−27.1; +45.2	−23.3; +42.9	−40.3; +21.9	-
Excluded	capreomycin IB ^$^	3; 4; 4	int	71.0 (13.5; 43.9)	46.2 (12.7; 29.4)	33.5 (19.5; 32.5)	−128.2; +70.2	−111.8; +4.2	−130.0; −3.0	-
	capreomycin IA ^$^	3; 4; 4	int	61.5 (12.6; 47.0)	38.6 (12.4; 29.5)	27.7 (20.0; 34.7)	−143.8; +66.8	−119.4; −3.4	−139.5; −5.0	-
	kanamycin	3; 3; 3	int	103.7 (5.6; 6.8)	114.7 (3.7; 10.4)	95.8 (10.6; 30.0)	−11.2; +18.6	−9.1; +38.5	−72.8; +64.4	-

Val.: validation; ext/int: external/internal validation; wthn-day prec.: within-day precision; btw-day prec.: between-day precision; % CV: percent coefficient of variation; * normalized to D-delamanid; ^$^ normalized to gentamicin; ^§^ amikacin was evaluated based on only one set of QC_low_, QC_med_, and QC_high_, stratified between-day precision and stratified 80%-beta intervals could not be calculated.

**Table 6:** TDM target values, resistance and minimal inhibitory concentration testing as well as dosing regimens and resulting TDM parameters for patient 1 T1 and patient 2 T3.

		TDM Target		Patient 1 T1				Patient 2 T3			
	Drug	Parameter	Value	DST ^§^	MIC ^§^	Dose [mg] ^§^	TDM1	DST	MIC	Dose [mg]	TDM3
Group A	levofloxacin	AUC/MIC	>146	R	4	-	-	R	7.5	-	-
	moxifloxacin	*f*AUC/MIC	>53	r	2	1200	21.26	r	2	1600	18.49
	bedaquiline	AUC/MIC	>74.6	S	0.015	200	2703.33	r	2	300	54.03
	linezolid	*f*AUC/MIC	>125	S	0.25	600	273.53	R	>1	-	-
Unclassified	pretomanid	*-*	-	-	-	-	-	-	-	-	-
Group B	clofazimine	C_min_; C_max_ * [µg/mL]	0.52; 0.79	S	0.12	100	0.25; 0.52	r	2	200	0.54; 0.68
	cycloserine ^$^	% T > MIC [%]	>30	-	4	750	100	-	30	1000	80.67
Group C	ethambutol	AUC/MIC	>119	R	10	-	-	R	7.5	-	-
	delamanid	C_min_; C_max_ * [µg/mL]	0.17; 0.31	R	>0.48	-	-	S	<0.06	2 × 100	0.32; 0.58
	pyrazinamide	AUC/MIC	>8.42	R	>100	-	-	R	>100	-	-
	meropenem	% T > MIC [%]	>60 %	-	-	3 × 2000/2 h	62.3 (MIC:8) ^§^	R	16	3 × 2000/3 h	46.30
	clavulanic acid	*-*	-	-	-	3 × 125	-	-	-	3 × 125	-
	amikacin	C_max_/MIC	>75	S	<0.25	-	-	R	>1	-	-
	streptomycin	*f*C_max_/MIC	>20	S	-	-	-	R	-	-	-
	prothionamide	AUC/MIC	>56.2	R	2	-	-	R	>5	-	-
	PAS	C_max_ * [µg/mL]	300–500	S	<4	-	-	R	>16	11820	440.38

TDM: therapeutic drug monitoring; T1/T3: first and third TDM cycles, respectively; DST: drug-susceptibility testing; MIC: minimal inhibitory concentration; *f*AUC: (unbound/free) area under the concentration–time curve; C_min_: (unbound/free) minimal or trough concentration; Cmax: maximal or peak concentration; % T > MIC: percentage time in which the concentration exceeds the MIC; R: resistant; r: low-level resistant; S: susceptible; * for clofazimine, delamanid, and intravenously administered PAS, no TDM target parameters were available and the concentration range (C_min_; C_max_) or C_max_ were compared to normal values; ^$^ administered as terizidone; ^§^ no MIC testing available, % T > MIC is displayed for the highest MIC for which % T > MIC was within target.

## Data Availability

The data presented in this study are available on request from the corresponding author.

## Supplementary Materials

The following supporting information can be downloaded at: https://www.mdpi.com/article/10.3390/pharmaceutics15112543/s1. Table S1: Analyte information; Table S2: Donor characteristics and clinical routine laboratory parameters; Table S3: MS settings; Figure S1: MRM channels, channel activation and duty cycle duration; Table S4: HPLC gradient; Table S5: Calculation of chromatographic parameters; Table S6: Chromatographic parameters of analyte peaks; Table S7: MS2 scans of every compound; Figure S2: Peak tailing of moxifloxacin and D-moxifloxacin; Table S8: Calibration curves; Figure S3: Selectivity: Analyte-, IS-, and matrix signal at LLOQtarget; Table S9: Specificity assessment by comparing analyte mass transitions with fragmentation mass spectra of common co-medications; Figure S4: Model-based calculation of meropenem % T > MIC.
