# Supplementary material for: A Single-Run HPLC–MS Multiplex Assay for Therapeutic Drug Monitoring of Relevant First- and Second-Line Antibiotics in the Treatment of Drug-Resistant Tuberculosis
Source: Pharmaceutics. 2023 Oct 27;15(11):2543. doi: 10.3390/pharmaceutics15112543 (PMC10674734; doi:10.3390/pharmaceutics15112543)
Supplement: Supplementary file 1 [file pharmaceutics-15-02543-s001.zip › pharmaceutics-2661554-supplementary.pdf]

Table S1. Analyte information.

WHO group	Analyte	Product name	Producer	order number	dissolved in	C_{IS} in extraction solvent [µg/ml]	C_{IS} in extracted sample [µg/ml]
Pan-susceptible	rifampicin		Sigma Aldrich	R3501-250mg	ACN	-	-
	D-rifampicin	$^{2}H_{8}$-rifampicin	Alsachim	C2892	ACN	0.2143	0.15
	rifabutin		Sigma Aldrich	R3530-5mg	ACN	-	-
	D-rifabutin	$^{2}H_{6}$-rifabutin	Alsachim	C722	ACN	0.010714	0.0075
	isoniazid		Sigma Aldrich	I3377-5g	ACN/H_2O	-	-
	D-isoniazid	$^{2}H_{4}$-isoniazid	Alsachim	C4675	ACN/H_2O	0.10714	0.075
						-	
Group A	levofloxacin		Sigma Aldrich	28266	ACN/H_2O	-	-
	D-levofloxacin	^{13}C-$^{2}H_{3}$-levofloxacin	Alsachim	C1175	ACN/H_2O	0.17857	0.125
	moxifloxacin	moxifloxacin hydrochloride	Molekula GmbH	85126157-1g	ACN/H_2O	-	-
	D-moxifloxacin	$^{2}H_{5}$-moxifloxacin trifluoroacetate	Alsachim	C4633	ACN/H_2O	0.0714	0.05
	bedaquiline		Adooq	A12327-5	ACN	-	-
	D-bedaquiline	$^{2}H_{6}$-bedaquiline	Alsachim	C7342	ACN	0.02143	0.015
	linezolid		Sigma Aldrich	PZ0014-5mg	ACN	-	-
	D-linezolid	$^{2}H_{8}$-linezolid	Alsachim	C1040	ACN	0.10714	0.075
Un-clssfd	pretomanid		Sigma Aldrich	SML1290-10MG	ACN	-	-
						-	
Group B	clofazimine		Sigma Aldrich	C8895-1g	ACN	-	-
	D-clofazimine	$^{2}H_{6}$-clofazimine	Alsachim	C7167	ACN	0.010714	0.0075
	cycloserine		Sigma Aldrich	C3909-25mg	H_2O	-	-
	D-cycloserine	$^{2}H_{3}$-^{15}N-cycloserine tartaric acid	Alsachim	C7114	H_2O	0.286	0.2
Group C	ethambutol	ethambutol dihydrochloride	Sigma Aldrich	E4630-25mg	ACN/H_2O	-	-
	D-ethambutol	$^{2}H_{4}$-ethambutol dihydrochloride	Alsachim	C7385	ACN/H_2O	0.0571	0.04

	delamanid		BioVision	2280-5	ACN	-	-
	D-delamanid	$^{2}H_{5}$-delamanid	Alsachim	C7346	ACN	0.010714	0.0075
	pyrazinamide		Sigma Aldrich	P7136-10g	ACN	-	-
	D-pyrazinamide	$^{13}C_{2}$-$^{15}N_{2}$-pyrazinamide	Alsachim	C6595	ACN	0.286	0.2
	meropenem	meropenem trihydrate	Sigma Aldrich	32460-25mg	H_2O	-	-
	D-meropenem	$^{2}H_{6}$-meropenem	Alsachim	C1298	H_2O	0.2143	0.15
	clavulanic acid	clavulanate lithium	Sigma Aldrich	1134426-200mg	H_2O	-	-
	amikacin		Sigma Aldrich	A0368000	H_2O	-	-
	D-amikacin	$^{2}H_{5}$-amikacin hydrochloride	Alsachim	C6821	H_2O	0.714	0.5
	streptomycin	streptomycin sulphate	Sigma Aldrich	S6501-5g	H_2O	-	-
	prothionamide		Sigma Aldrich	SMB00387-1g	ACN/H_2O	-	-
	D-prothionamide	$^{2}H_{5}$-prothionamide	Alsachim	C7344	ACN/H_2O	0.0286	0.02
	PAS		Sigma Aldrich	A79604-5G	H_2O	-	-
	D-PAS	$^{13}C_{6}$-p-aminosalicylic acid	Alsachim	C7345	H_2O	0.286	0.2
						-	
excluded	capreomycin IB*	capreomycin sulphate	Sigma Aldrich	C4142-1g	H_2O	-	-
	capreomycin IA*						
	kanamycin	kanamycin sulphate	Sigma Aldrich	K1377-1g	H_2O	-	-
	D-kanamycin	$^{13}C_{6}$-kanamycin A	Alsachim	C7347	H_2O	0.714	0.5
						-	
Non-D int. Std.	gentamicin	gentamicin sulphate	Sigma Aldrich	G1940-250mg	H_2O	4.3	3

#: drug number; **C_{IS}**: concentration of internal standard; D-: stable heavy hydrogen- or heavy carbon or -nitrogen labelled internal standards; $^{X}H_{Y}$, $^{X}C_{Y}$, and $^{X}N_{Y}$: X is the atomic mass of the stable isotope, Y the number of isotopes in the molecule; ACN: HPLC-grade acetonitrile; H_2O: HPLC-grade water; * capreomycin IB and IA are two out of four cyclic peptides with antimicrobial activity that are contained in the drug preparation of capreomycin.

Table S2. Donor characteristics and clinical routine laboratory parameters.

parameter		unit	Pool	P1	P2	P3	P4	P5	P6
age				29	33	47	29	26	32
weight				71	59	93	71	83	94
height				1.84	1.63	1.68	1.83	1.86	1.76
BMI		kg/m^2	donor 1: 25.8 donor 2: 25.2 donor 3: 22.9	20.98	22.21	32.95	20.52	23.99	30.35
potassium*		mmol/l	3.40	3.8	4	4.2	4.5	4.3	4.6
sodium		mmol/l	160	141	138	138	140	141	139
chloride		mmol/l	95	104	99	100	101	103	101
calcium*		mmol/l	<1.25	2.27	2.57	2.27	2.3	2.34	2.39
magnesium*		mmol/l	0.41	0.75	0.82	0.7	0.77	0.78	0.94
urea		mg/dl	29						
uric acid		mg/dl	3.0						
creatinine		mg/dl	0.64						
total bilirubin		mg/dl	0.4						
direct bilirubin		mg/dl	0.1						
indirect bilirubin		mg/dl	0.3						
cholesterol		mg/dl	166	160	222	186	161	140	175
triglycerides		mg/dl	38	58	173	104	262	39	149
HDL		mg/dl	69	73	70	61	44	51	49
LDL		mg/dl	89	75	117	104	65	81	96
total protein		g/l	65	74	78	75	85	80	81
protein fractions	albumin	%	57.7						
	α_1	%	3.3						
	α_2	%	7.5						
	β_1	%	6.0						
	β_2	%	10.6						
	γ	%	15.0						

Pool: pooled plasma of three female donors; P1-P6: individual donors 1-6. BMI: body mass index; * blood was collected using K_2EDTA as anticoagulant, potassium might be artificially increase, calcium and magnesium complex-bound and artificially decreased.

Table S3. Mass spectrometer settings.

Parameter	Value
**Cone gas flow**	100 l/h
**Desolvation gas flow**	800 l/h
**Extractor voltage**	3.0 V
**Capillary voltage**	3.0 kV
**Source temperature**	90 °C
**Desolvation gas temperature**	450 °C

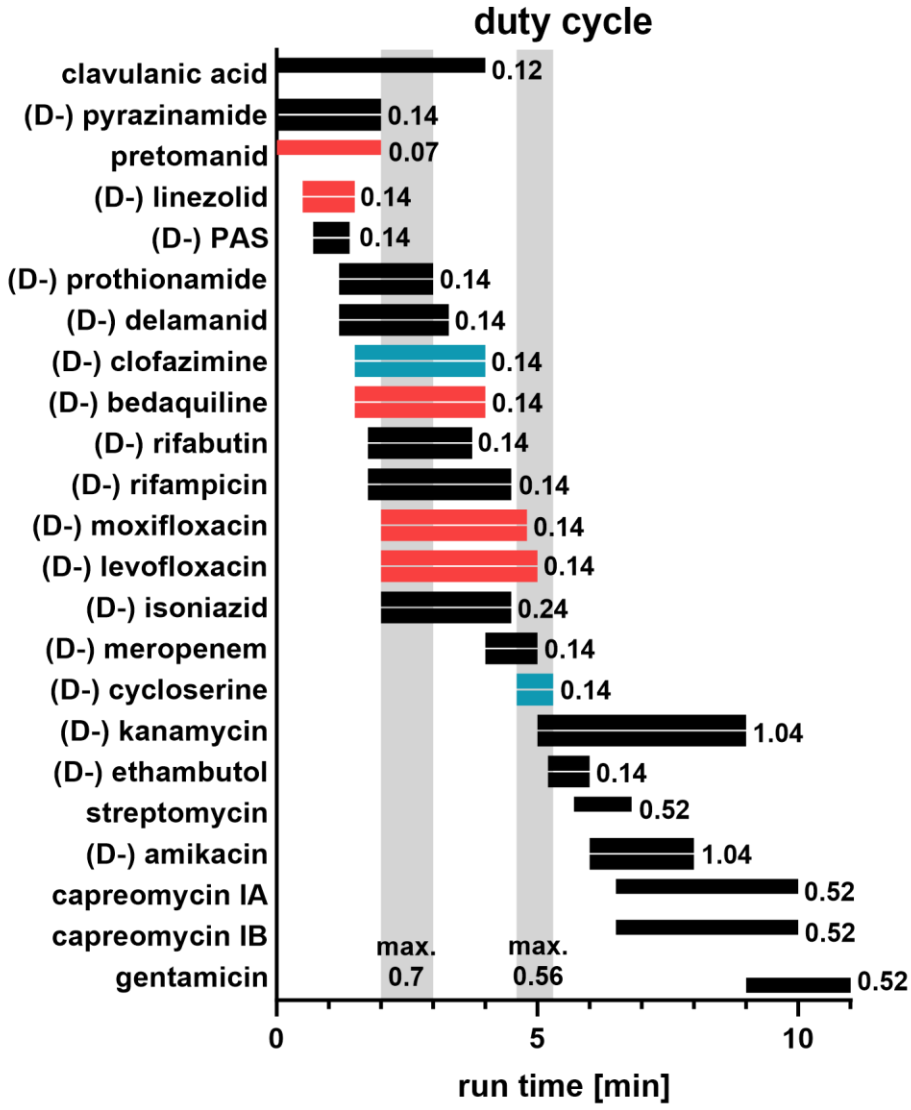

Figure S1. MRM channels, channel activation and duty cycle duration. Activation of the MRM channels over time. Start and end of each bar represent the start and end of the respective MRM channel. Identical stacked bars represent channels for an analyte and the respective stable isotope-labelled (D-) internal standard, e.g. pyrazinamide and D-pyrazinamide. Numbers indicate the total dwell time (dwell time + inter-channel delay) per (double-) channel. The duty cycle is the sum the total dwell time of all activated channels at a given run time. Grey areas indicate the run times with maximum duty cycle duration. During the first maximum, channels for (D-) prothionamide, (D-) delamanid, (D-) clofazimine, (D-) bedaquiline, and either (D-) moxifloxacin or (D-) levofloxacin would be activated resulting in a maximum duty cycle duration of 0.7 seconds. During the second maximum, channels for either (D-) moxifloxacin or (D-) levofloxacin, (D-) meropenem, (D-) cycloserine, and (D-) ethambutol were activated, resulting in a maximum duty cycle duration of 0.56 seconds. Red bars: group A drugs, turquoise bars: group B drugs, black bars: first-line, group C and WHO-excluded drugs.

Table S4. HPLC gradient.

Time [min]	A: 1% FA [%]	B: ACN [%]	Flow rate [mL/min]
**0.0**	10	90	0.5
**1.0**	10	90	0.5
**4.0**	98	2	0.5
**4.1**	98	2	0.8
**15.0**	98	2	0.8
**24.0**	10	90	0.8
**25.0**	10	90	0.5
**29.0**	10	90	0.5

HPLC: high-performance liquid chromatography; 1% FA: 1% formic acid in HPLC-grade water; ACN: HPLC-grade acetonitrile.

Table S5. Calculation of chromatographic parameters.

Parameter	Formula
Peak width at baseline *W*	$W = t_b - t_a$<br>*W*: peak with at baseline<br>t_a: peak start<br>t_b: peak end
Retention factor *k*	$k = \frac{t_r - t_0}{t_0}$<br>*k*: retention factor<br>$t_0$: void time, retention time of an unretained analyte<br>t_r: retention time
Asymmetry factor A_S	$A_S = \frac{b}{a}$<br>with<br>$a = t_r - t_a$<br>$b = t_b - t_r$<br>A_S: Asymmetry factor<br>*b*: trailing edge<br>*a*: leading edge
Column efficiency *N*	$N = 16\left(\frac{t_r}{W}\right)^2$<br>*N*: Column efficiency<br>$t_r$: retention time<br>*W*: peak width at baseline

Formulas according to (1).

Table S6. Chromatographic parameters of analyte peaks.

WHO group	Analyte	Retention time t_r [min] median (90% range)	Peak width W [min] median (90% range)	Retention factor k median (90% range)	Asymmetry factor A_S median (90% range)	Column efficiency N median (90% range)
**First-line**	rifampicin	2.60 (2.43-2.89)	0.52 (0.46-0.68)	2.55 (2.36-2.96)	1.39 (0.92-1.82)	384 (252-475)
	rifabutin	2.91 (2.79-3.61)	0.62 (0.35-0.71)	3.02 (2.84-3.96)	1.58 (1.16-2.06)	341 (264-1655)
	isoniazid	2.81 (2.70-3.40)	0.64 (0.57-1.17)	2.86 (2.71-3.64)	1.89 (1.53-2.36)	308 (87-419)
**Group A**	levofloxacin	4.25 (4.08-4.51)	0.60 (0.41-1.13)	4.84 (4.66-4.99)	2.53 (1.75-4.42)	794 (218-1794)
	moxifloxacin	3.18 (2.77-4.20)	0.44 (0.28-0.97)	3.36 (2.81-4.77)	1.04 (0.47-1.85)	727 (273-3651)
	bedaquiline	2.52 (2.08-3.15)	0.56 (0.41-0.66)	2.47 (1.86-3.33)	1.46 (1.03-1.78)	308 (241-937)
	linezolid	1.06 (0.96-1.10)	0.55 (0.48-0.62)	0.47 (0.42-0.51)	1.57 (1.17-2.20)	60 (45-81)
**Unclassified**	pretomanid	1.00 (0.95-1.05)	0.43 (0.36-0.50)	0.37 (0.31-0.43)	2.71 (2.03-3.62)	89 (60-129)
**Group B**	clofazimine	2.41 (2.02-2.88)	0.71 (0.54-1.02)	2.14 (1.78-2.95)	2.01 (1.34-3.07)	174 (87-341)
	cycloserine	4.99 (4.92-5.10)	0.12 (0.10-0.18)	5.85 (5.76-6.00)	1.05 (0.58-1.80)	28021 (12492-45033)
**Group C**	ethambutol	5.48 (5.45-5.62)	0.20 (0.14-0.33)	6.55 (6.49-6.75)	1.24 (0.81-2.47)	12759 (4624-23775)
	delamanid	1.87 (1.70-2.51)	0.51 (0.45-0.56)	1.57 (1.32-2.45)	1.65 (1.20-2.12)	214 (176-434)
	pyrazinamide	1.19 (1.16-1.21)	0.35 (0.31-0.42)	0.64 (0.61-0.66)	1.81 (1.25-2.37)	183 (127-238)
	meropenem	4.58 (4.43-4.68)	0.17 (0.13-0.23)	5.30 (5.10-5.44)	1.01 (0.69-1.49)	11345 (6658-17538)
	clavulanic acid*	1.17*	-	-	-	-
	amikacin	6.94 (6.62-7.29)	0.50 (0.39-0.69)	8.54 (8.10-9.02)	1.30 (0.76-1.89)	3097 (1620-5174)
	streptomycin	6.12 (6.07-6.35)	0.33 (0.22-0.46)	7.41 (7.35-7.73)	1.48 (0.85-2.03)	5412 (2829-12341)
	prothionamide	1.76 (1.65-2.25)	0.63 (0.49-0.97)	1.39 (1.28-2.07)	1.91 (1.26-2.86)	135 (68-214)
	PAS	1.09 (0.98-1.21)	0.38 (0.18-0.44)	0.50 (0.42-0.66)	2.19 (0.81-2.72)	133 (93-644)

**Excluded**	capreomycin IB	7.60 (7.45-8.70)	0.72 (0.53-1.12)	9.45 (9.24-10.96)	1.41 (1.10-2.19)	1924 (899-3253)
	capreomycin IA	7.79 (7.61-8.97)	0.60 (0.46-0.78)	9.69 (9.46-11.33)	0.82 (0.65-1.63)	3288 (1627-4578)
	kanamycin	7.55 (7.46-8.27)	0.67 (0.52-1.08)	9.38 (9.26-10.36)	1.75 (0.97-3.21)	2007 (870-3399)

Peak width at baseline. * Clavulanic acid could not be quantified simultaneously and therefore had only scarce data on the chromatography parameters.

Table S7. MS2 scans of every compound.

**Rifampicin**<br>Parent: 823.46<br>Primary: 791.01<br>Polarity: ES+<br>Cone [eV]: 30<br>CE [eV]: 20<br>Inj. [µl/min]: 2	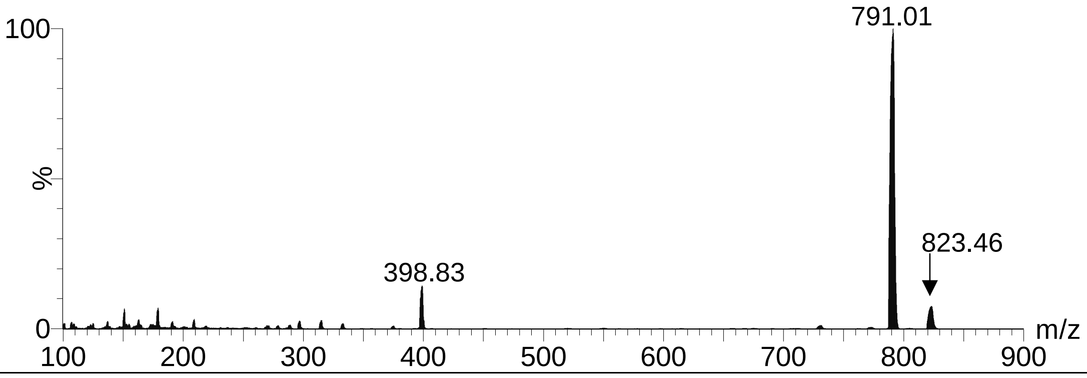
**D-Rifampicin**<br>Parent: 830.25<br>Primary: 798.69<br>Polarity: ES+<br>Cone [eV]: 30<br>CE [eV]: 20<br>Inj. [µl/min]: 2	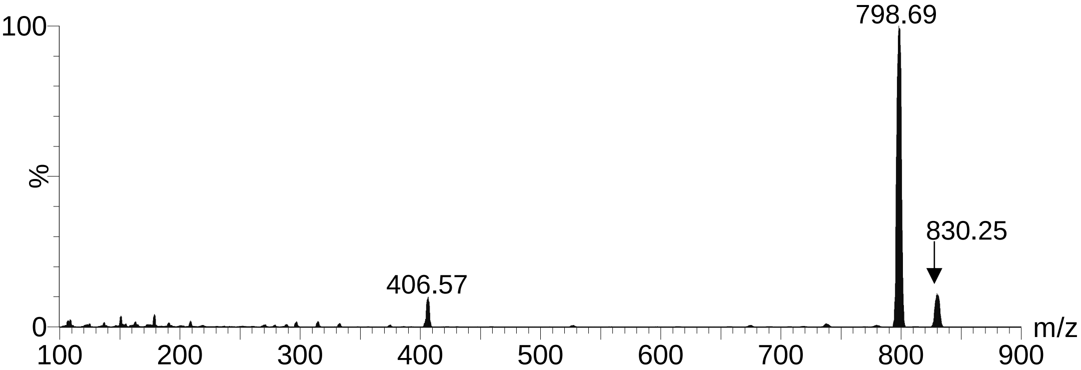
**Isoniazid**<br>Parent: 137.68<br>Primary: 78.50<br>Polarity: ES+<br>Cone [eV]: 25<br>CE [eV]: 25<br>Inj. [µl/min]: 2	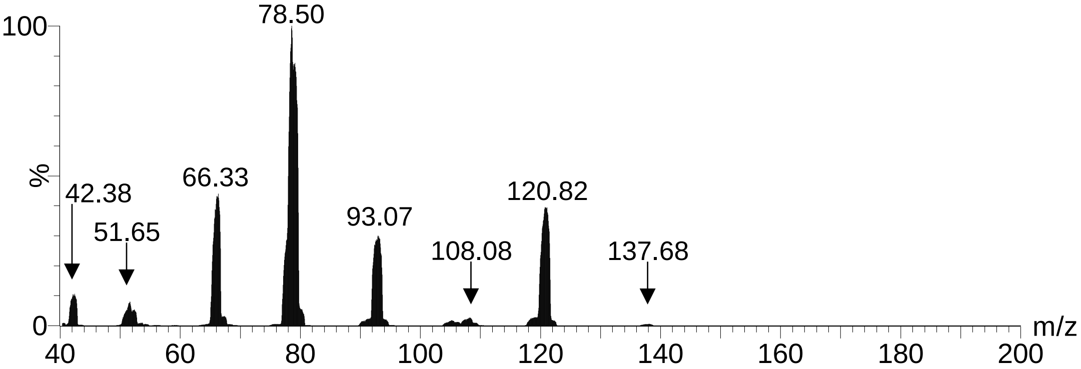
**D-Isoniazid**<br>Parent: 141.72<br>Primary: 82.60<br>Polarity: ES+<br>Cone [eV]: 25<br>CE [eV]: 25<br>Inj. [µl/min]: 2	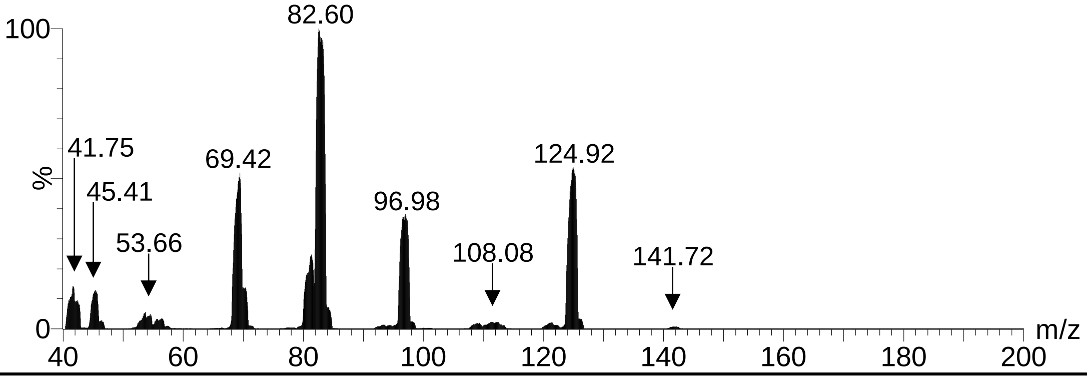

Rifabutin
Parent: 847.02
Primary: 815.52
Polarity: ES+
Cone [eV]: 40
CE [eV]: 35
Inj. [µl/min]: 2

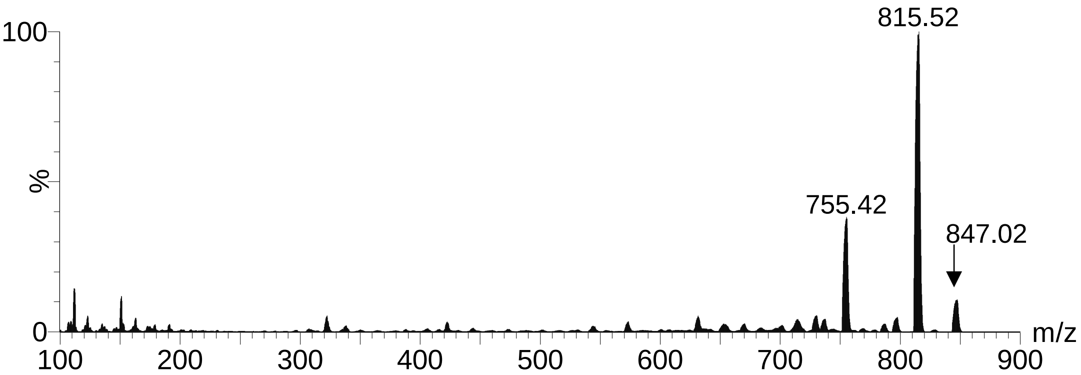

D-Rifabutin
Parent: 852.59
Primary: 821.16
Polarity: ES+
Cone [eV]: 40
CE [eV]: 35
Inj. [µl/min]: 2

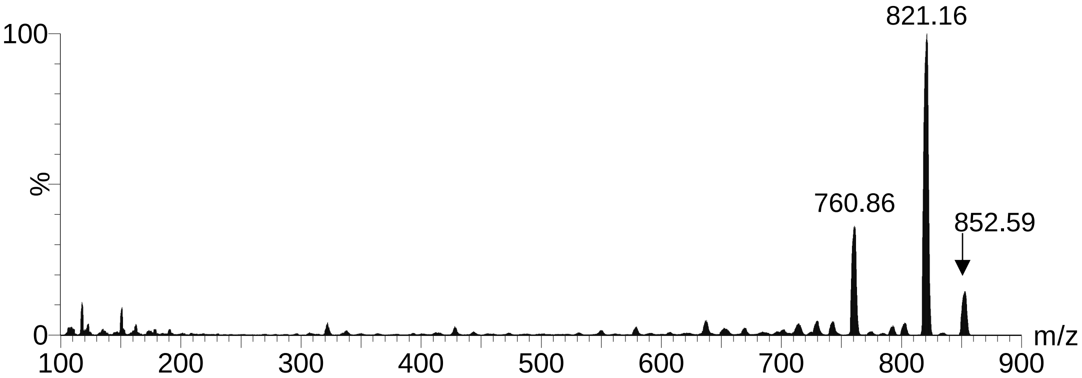

Levofloxacin
Parent: 361.82
Primary: 261.17
Polarity: ES+
Cone [eV]: 40
CE [eV]: 30
Inj. [µl/min]: 2

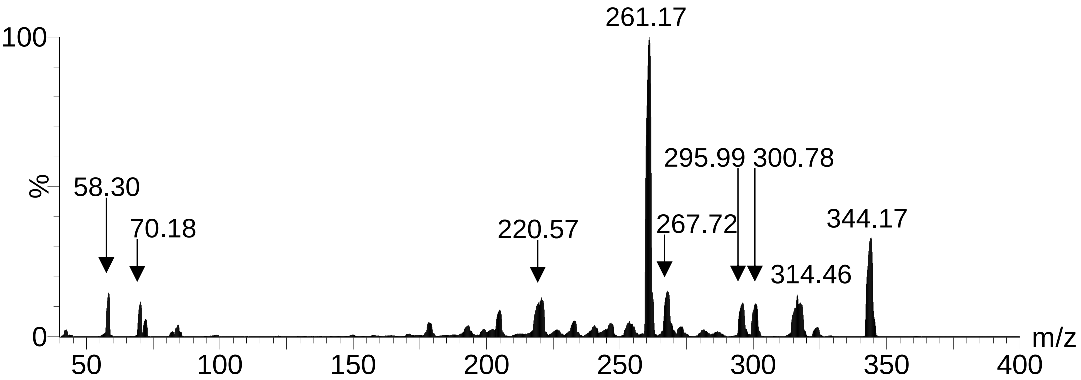

D-Levofloxacin
Parent: 366.04
Primary: 261.04
Polarity: ES+
Cone [eV]: 40
CE [eV]: 30
Inj. [µl/min]: 2

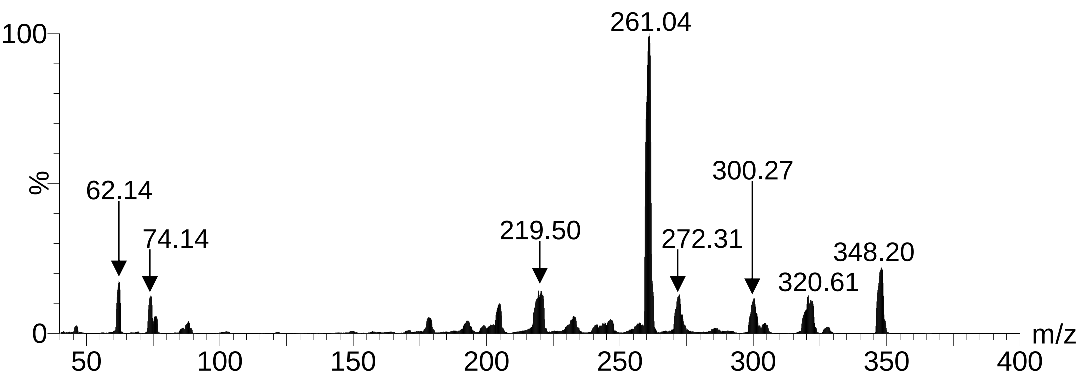

Moxifloxacin
Parent: 401.67
Primary: 364.21
Polarity: ES+
Cone [eV]: 40
CE [eV]: 35
Inj. [µl/min]: 2

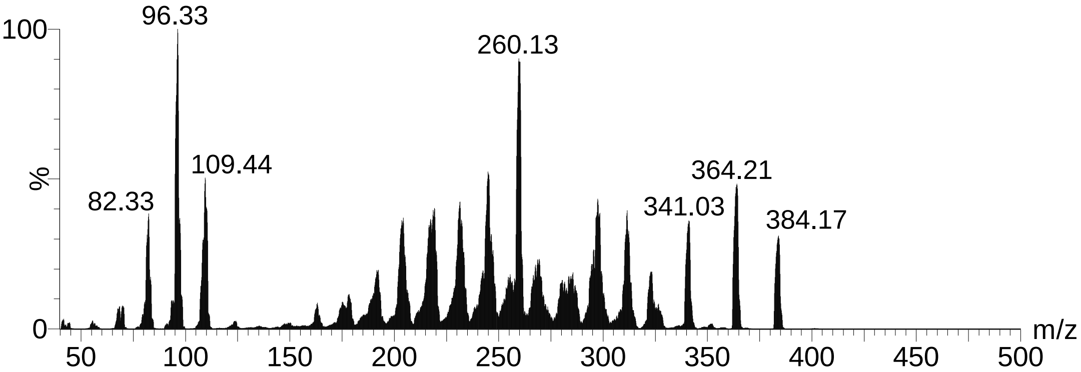

D-Moxifloxacin
Parent: 407.30
Primary: 369.11
Polarity: ES+
Cone [eV]: 40
CE [eV]: 35
Inj. [µl/min]: 2

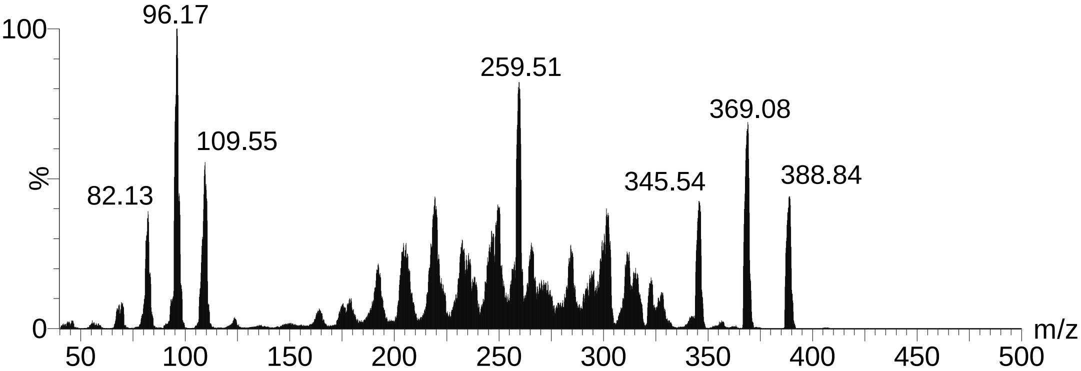

Bedaquiline
Parent: 555.14
Primary: 58.16
Polarity: ES+
Cone [eV]: 30
CE [eV]: 35
Inj. [µl/min]: 2

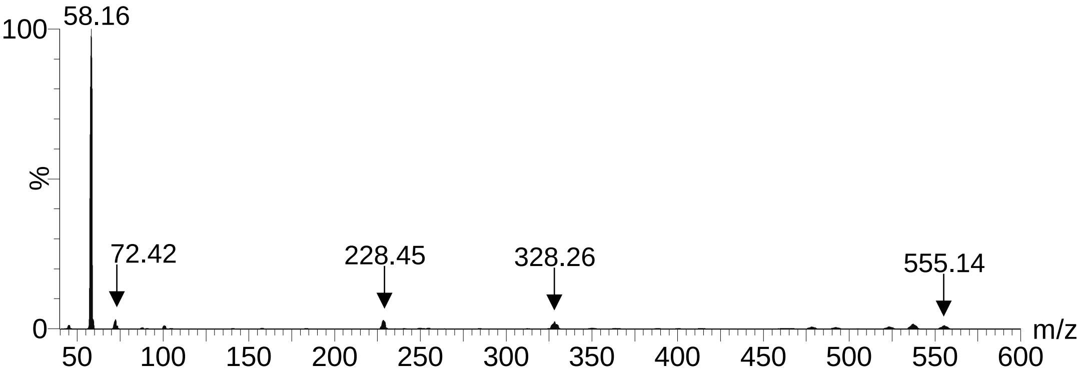

D-Bedaquiline
Parent: 561.13
Primary: 64.13
Polarity: ES+
Cone [eV]: 30
CE [eV]: 35
Inj. [µl/min]: 2

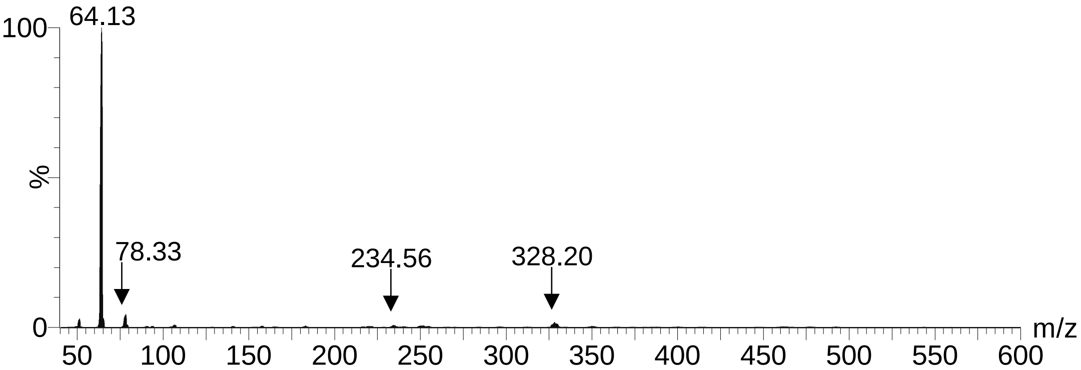

Linezolid
Parent: 337.17
Primary: 195.20
Polarity: ES+
Cone [eV]: 40
CE [eV]: 25
Inj. [µl/min]: 2

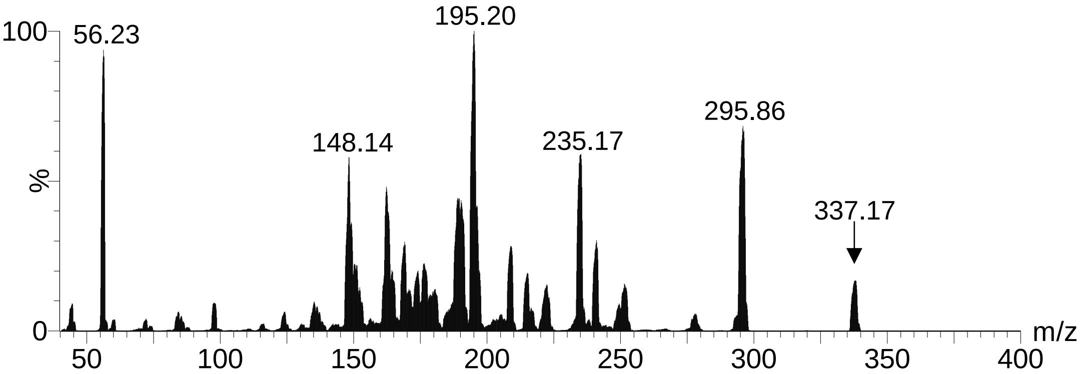

D-Linezolid
Parent: 345.10
Primary: 203.20
Polarity: ES+
Cone [eV]: 40
CE [eV]: 25
Inj. [µl/min]: 2

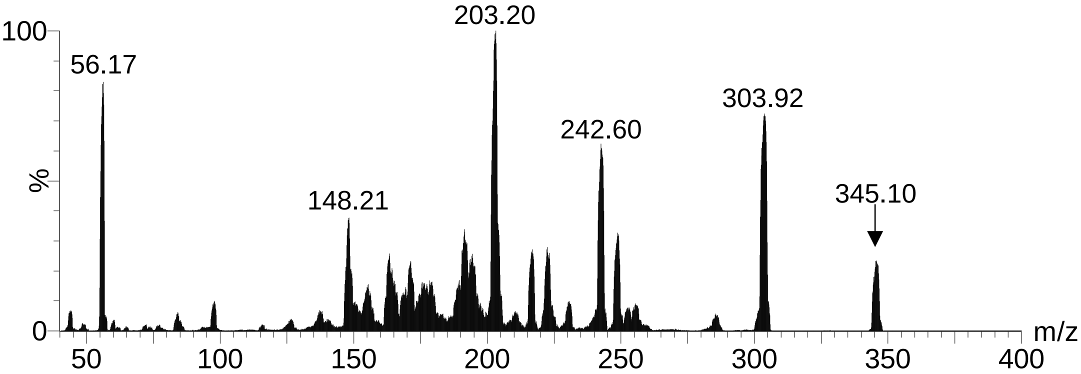

Pretomanid
Parent: 359.00
Primary: 174.82
Polarity: ES+
Cone [eV]: 35
CE [eV]: 25
Inj. [µl/min]: 2

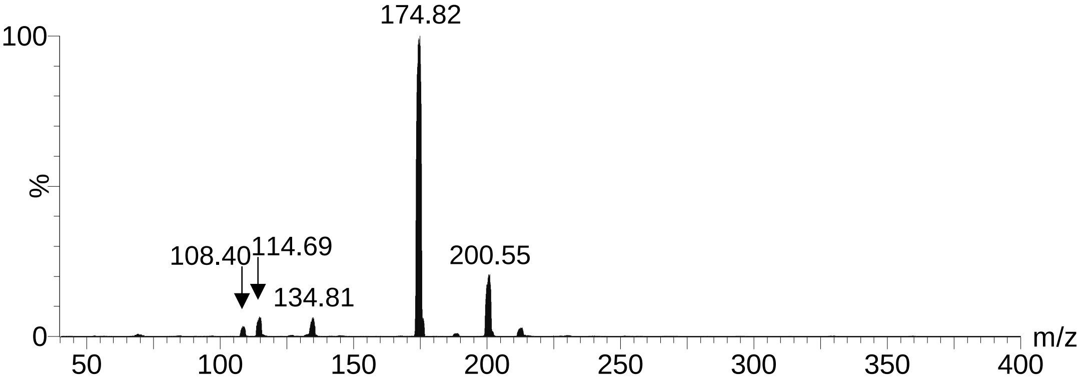

**Clofazimine**<br>Parent: 471.61<br>Primary: 395.25<br>Polarity: ES+<br>Cone [eV]: 60<br>CE [eV]: 45<br>Inj. [μl/min]: 2	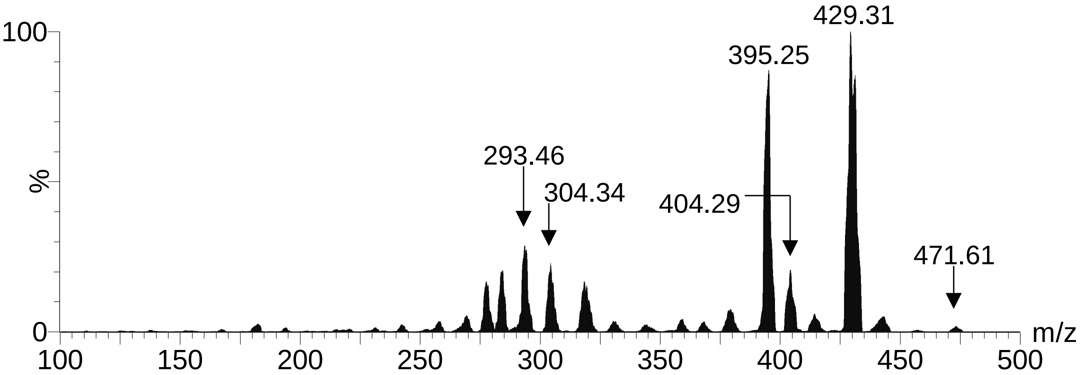
**D-Clofazimine**<br>Parent: 480.00<br>Primary: 396.15<br>Polarity: ES+<br>Cone [eV]: 60<br>CE [eV]: 45<br>Inj. [μl/min]: 2	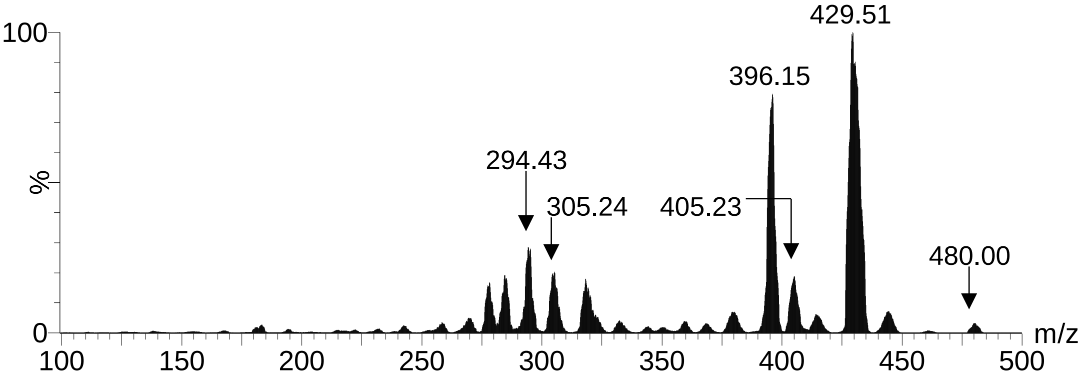
**Cycloserine**<br>Parent: 102.88<br>Primary: 75.03<br>Polarity: ES+<br>Cone [eV]: 25<br>CE [eV]: 10<br>Inj. [μl/min]: 2	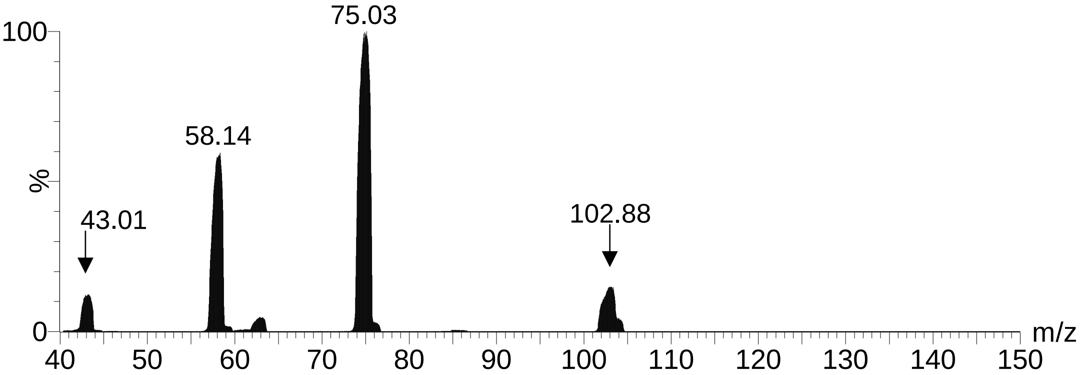
**D-Cycloserine**<br>Parent: 106.22<br>Primary: 78.85<br>Polarity: ES+<br>Cone [eV]: 25<br>CE [eV]: 10<br>Inj. [μl/min]: 2	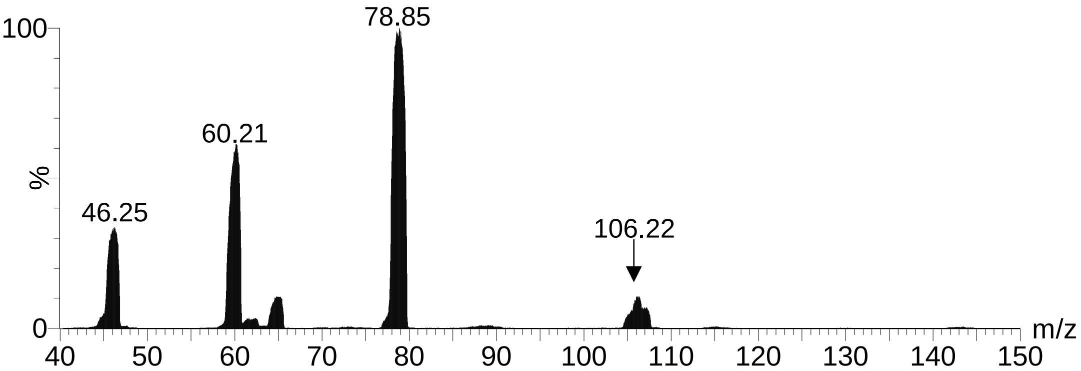

Ethambutol
Parent: 204.78
Primary: 44.29
Polarity: ES+
Cone [eV]: 20
CE [eV]: 25
Inj. [µl/min]: 2

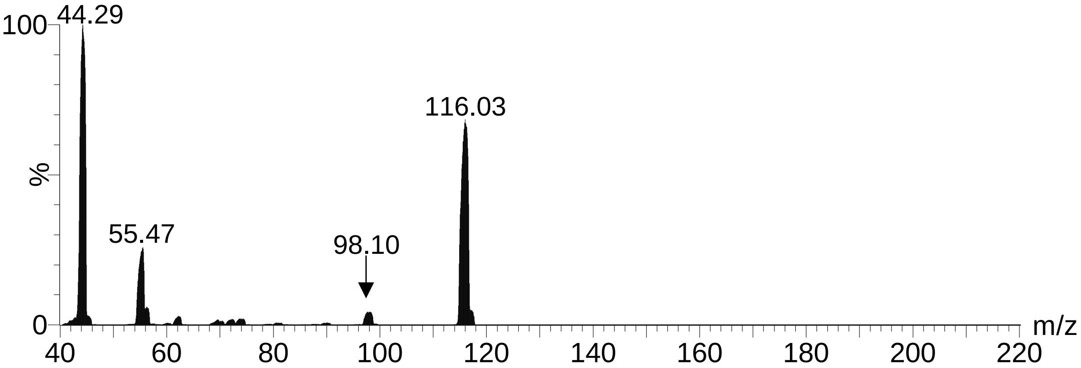

D-Ethambutol
Parent: 208.78
Primary: 48.29
Polarity: ES+
Cone [eV]: 20
CE [eV]: 25
Inj. [µl/min]: 2

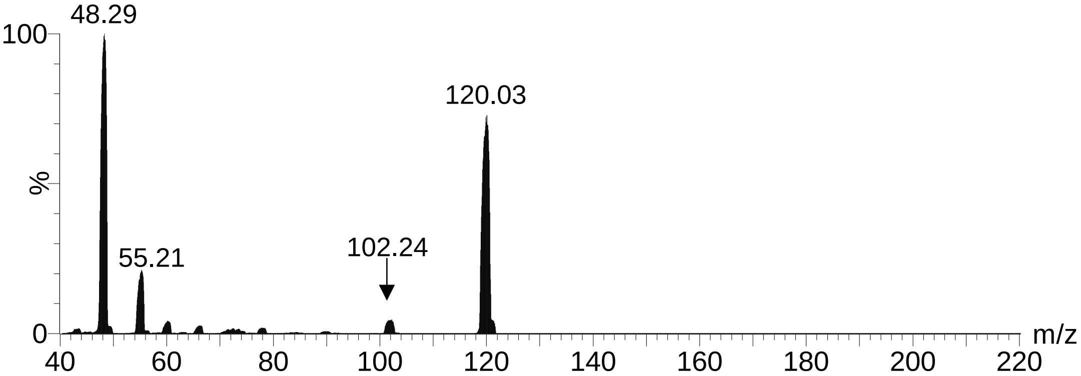

Delamanid
Parent: 534.37
Primary: 352.07
Polarity: ES+
Cone [eV]: 35
CE [eV]: 25
Inj. [µl/min]: 2

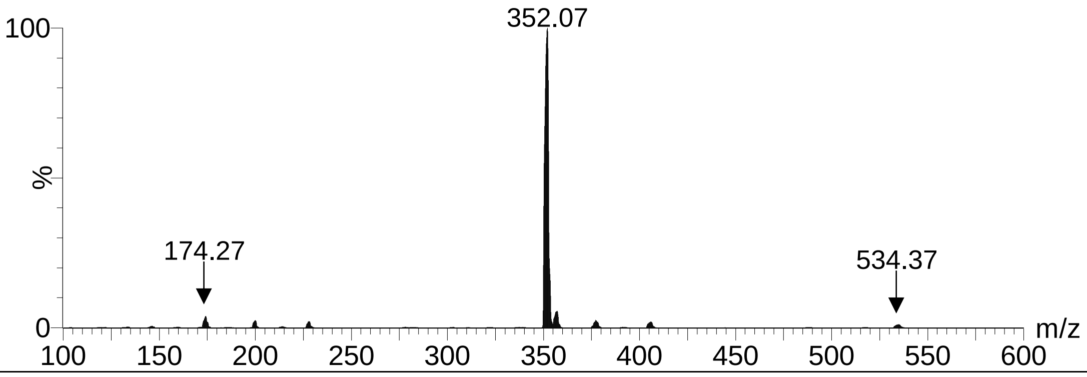

D-Delamanid
Parent: 538.30
Primary: 356.07
Polarity: ES+
Cone [eV]: 35
CE [eV]: 25
Inj. [µl/min]: 2

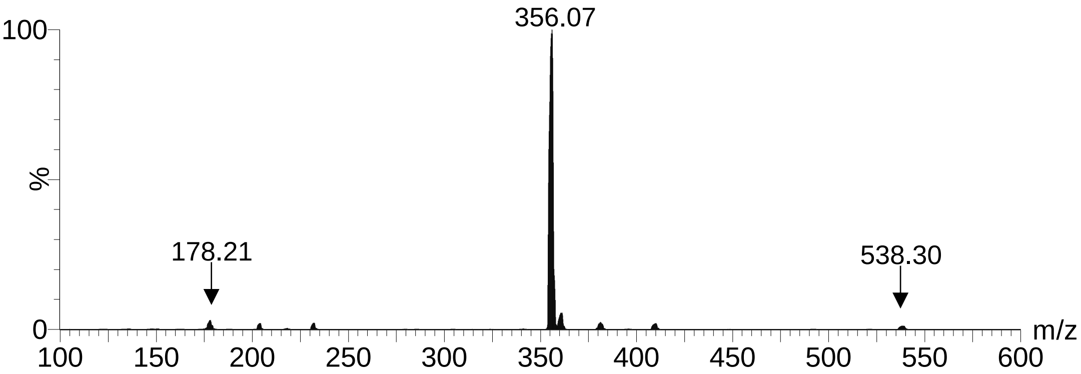

Pyrazinamide
Parent: 123.68
Primary: 78.88
Polarity: ES+
Cone [eV]: 20
CE [eV]: 20
Inj. [µl/min]: 10

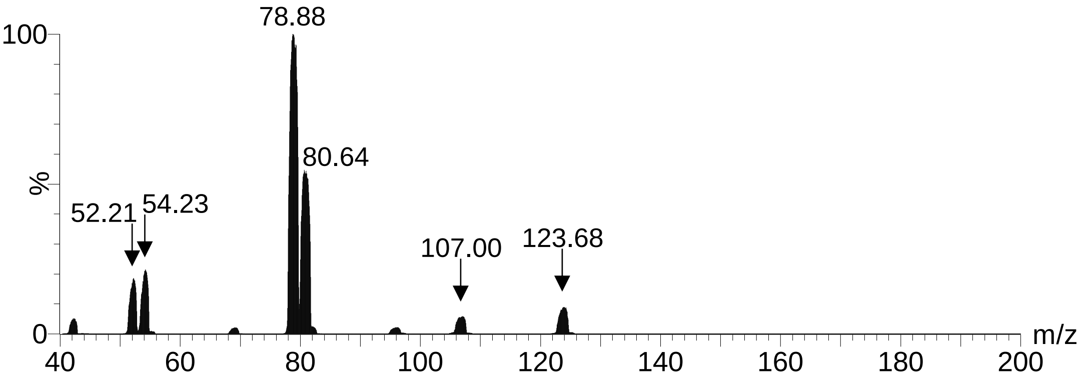

D-Pyrazinamide
Parent: 127.21
Primary: 82.85
Polarity: ES+
Cone [eV]: 20
CE [eV]: 20
Inj. [µl/min]: 10

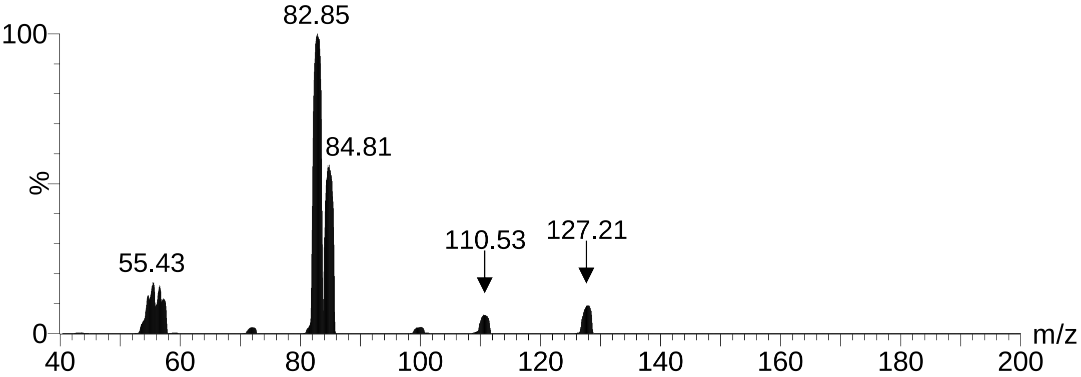

Meropenem
Parent: 383.95
Primary: 68.04
Polarity: ES+
Cone [eV]: 25
CE [eV]: 25
Inj. [µl/min]: 2

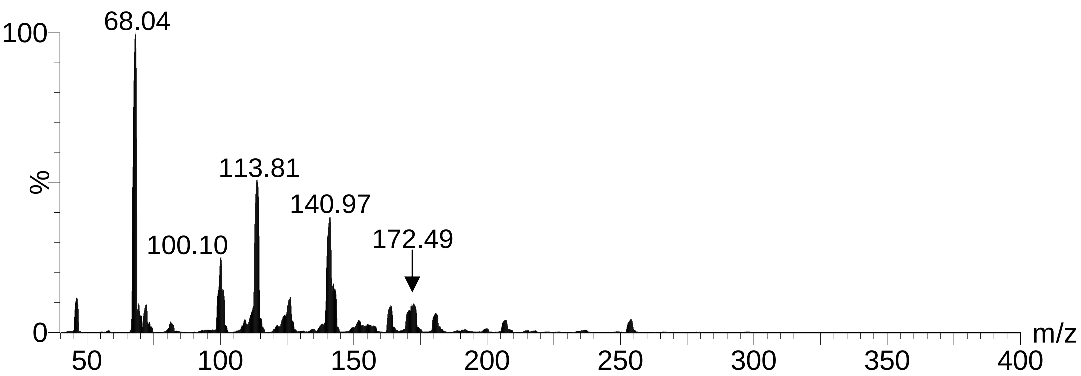

D-Meropenem
Parent: 389.96
Primary: 68.06
Polarity: ES+
Cone [eV]: 25
CE [eV]: 25
Inj. [µl/min]: 2

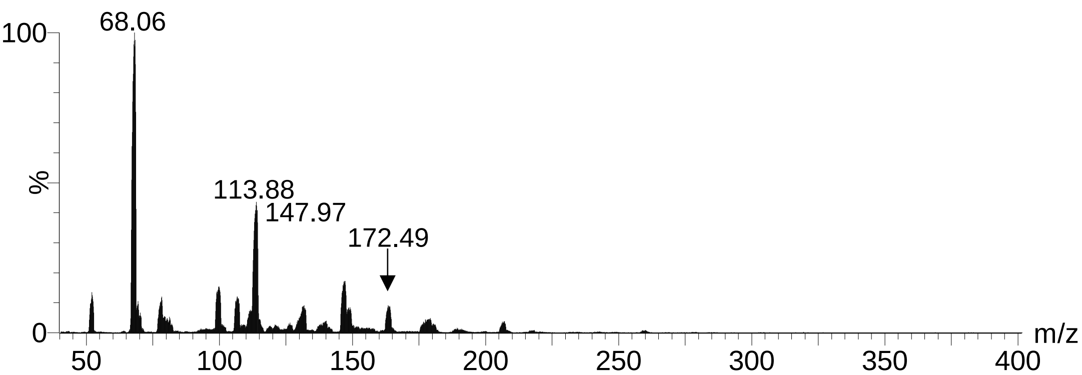

Amikacin
Parent: 585.06
Primary: 163.07
Polarity: ES+
Cone [eV]: 30
CE [eV]: 30
Inj. [µl/min]: 2

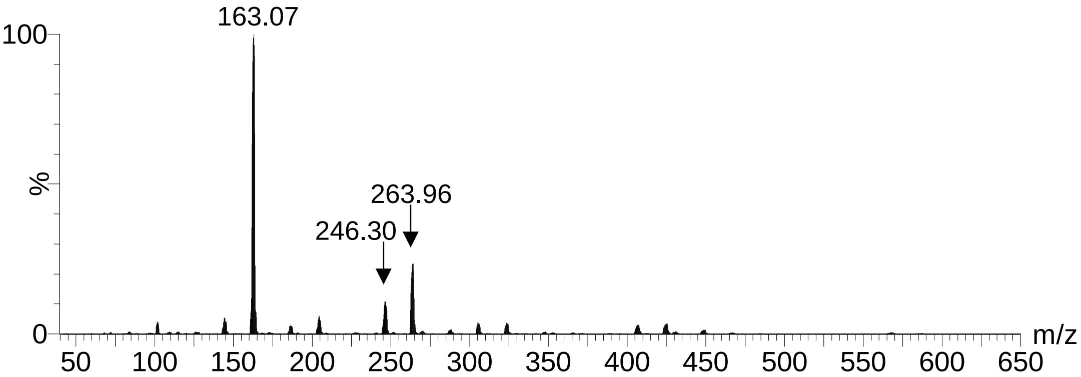

D-Amikacin
Parent: 590.11
Primary: 162.52
Polarity: ES+
Cone [eV]: 30
CE [eV]: 30
Inj. [µl/min]: 10

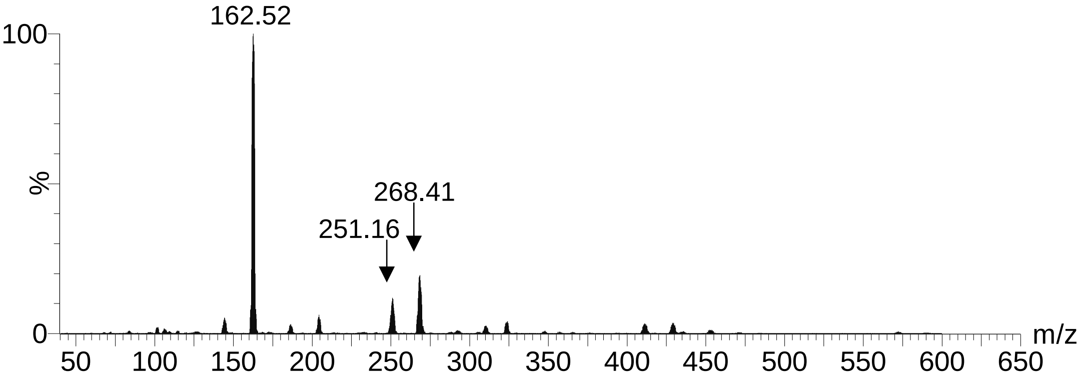

Streptomycin
Parent: 582.03
Primary: 263.10
Polarity: ES+
Cone [eV]: 65
CE [eV]: 35
Inj. [µl/min]: 2

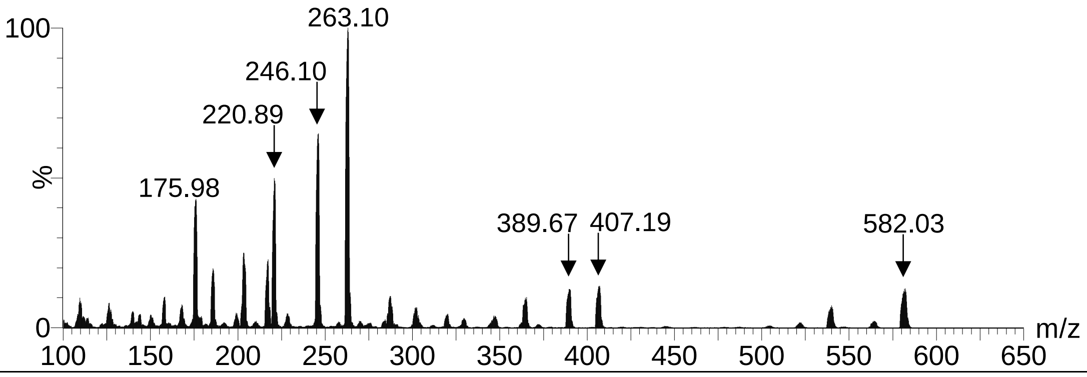

Prothionamide
Parent: 180.57
Primary: 120.94
Polarity: ES+
Cone [eV]: 35
CE [eV]: 25
Inj. [µl/min]: 2

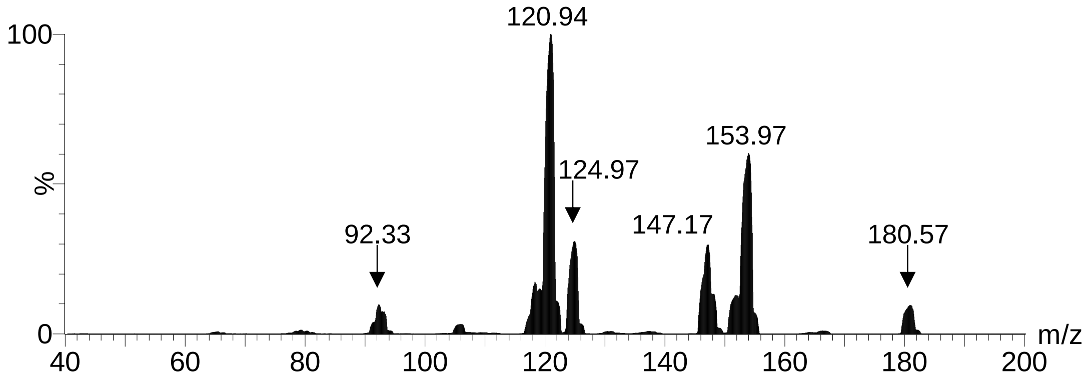

D-Prothionamide
Parent: 187.58
Primary: 127.21
Polarity: ES+
Cone [eV]: 35
CE [eV]: 25
Inj. [µl/min]: 2

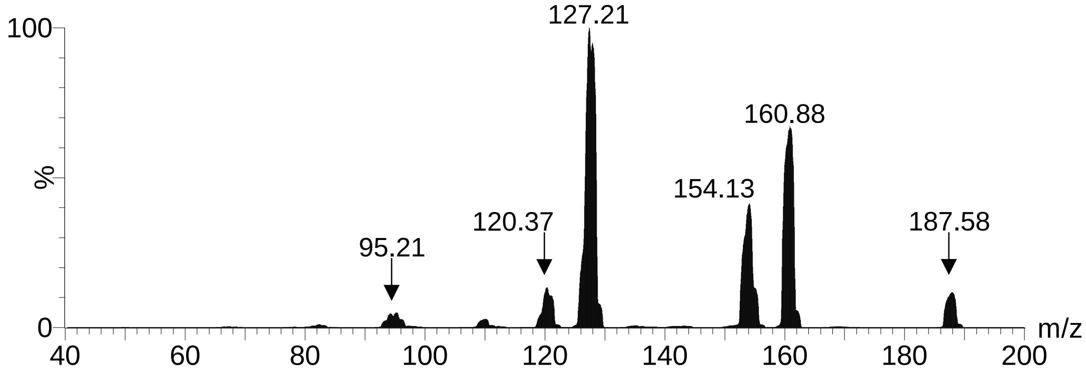

PAS
Parent: 153.64
Primary: 91.17
Polarity: ES+
Cone [eV]: 25
CE [eV]: 25
Inj. [µl/min]: 2

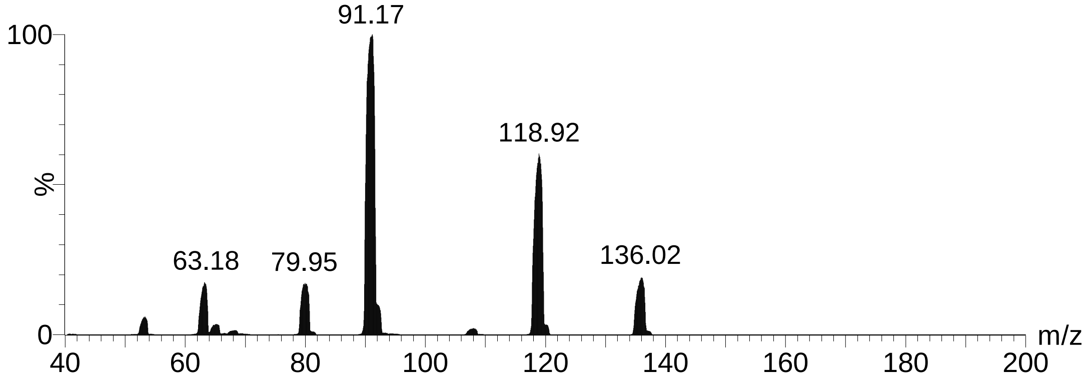

D-PAS
Parent: 159.94
Primary: 96.09
Polarity: ES+
Cone [eV]: 25
CE [eV]: 25
Inj. [µl/min]: 2

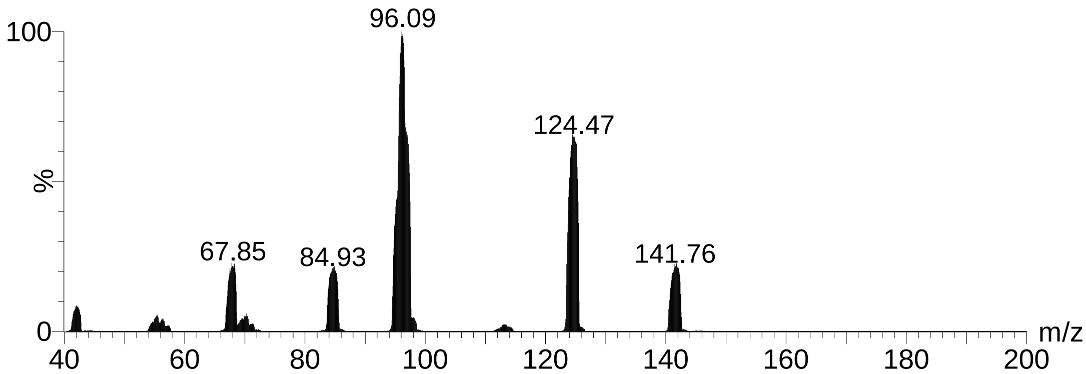

**Capreomycin IA**<br>Parent: 334.27<br>Primary: 70.24<br>Polarity: ES+<br>Cone [eV]: 25<br>CE [eV]: 20<br>Inj. [µl/min]: 5	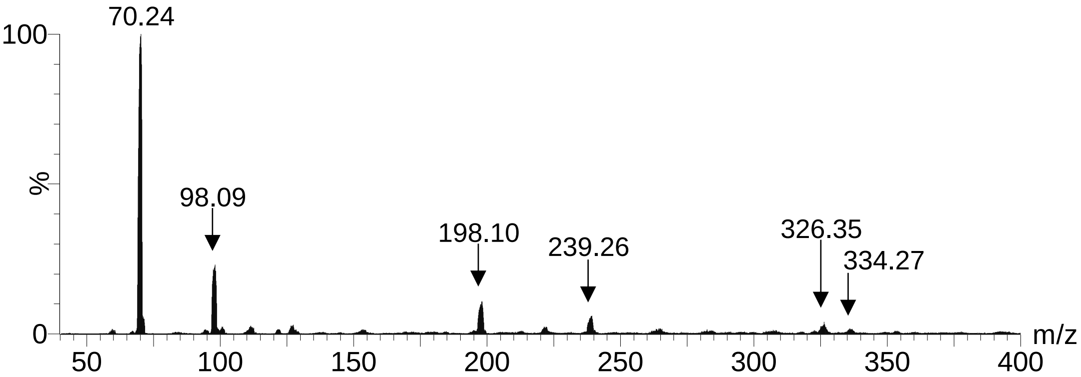
**Capreomycin IB**<br>Parent: 326.21<br>Primary: 70.18<br>Polarity: ES+<br>Cone [eV]: 25<br>CE [eV]: 20<br>Inj. [µl/min]: 5	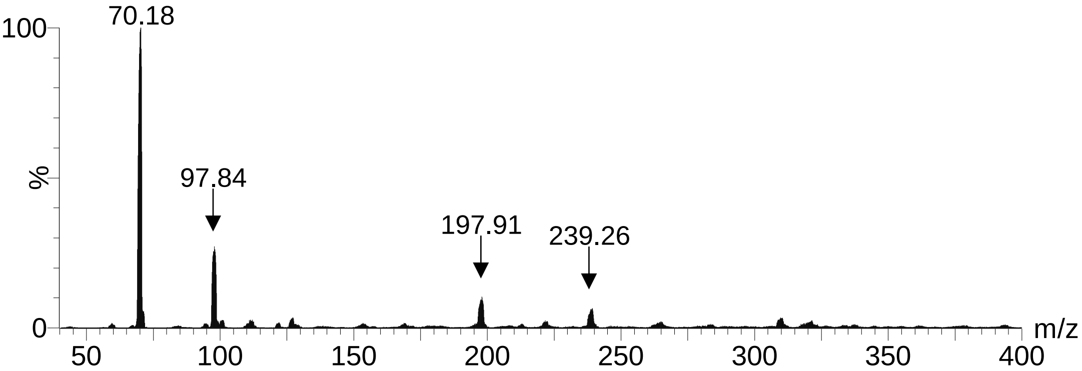
**Kanamycin**<br>Parent: 484.46<br>Primary: 162.48<br>Polarity: ES+<br>Cone [eV]: 35<br>CE [eV]: 25<br>Inj. [µl/min]: 2	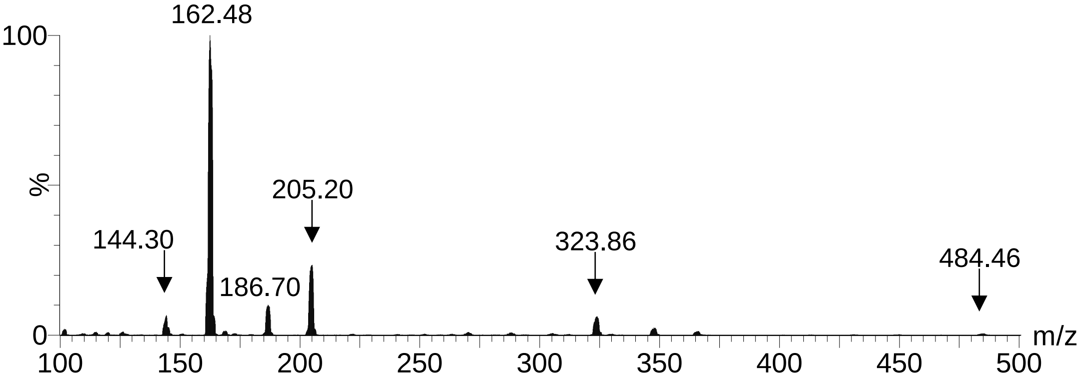
**D-Kanamycin**<br>Parent: 491.52<br>Primary: 162.52<br>Polarity: ES+<br>Cone [eV]: 25<br>CE [eV]: 25<br>Inj. [µl/min]: 2	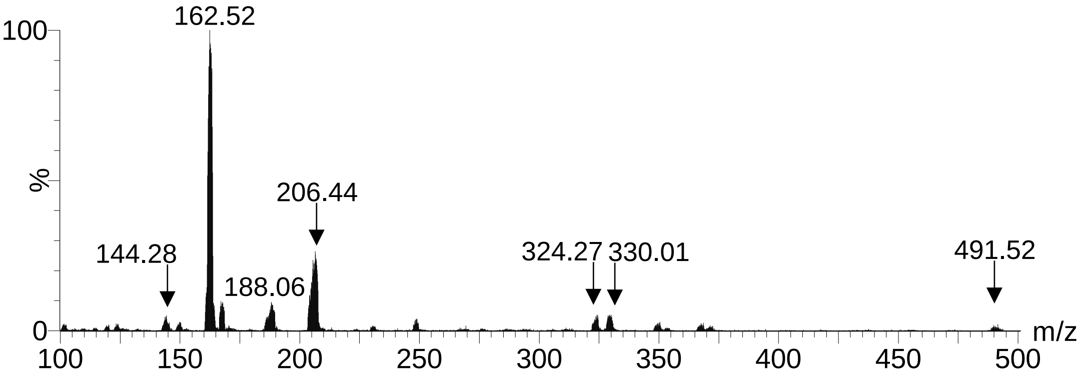

Gentamicin
Parent: 477.43
Primary: 157.15
Polarity: ES+
Cone [eV]: 30
CE [eV]: 20
Inj. [µl/min]: 10

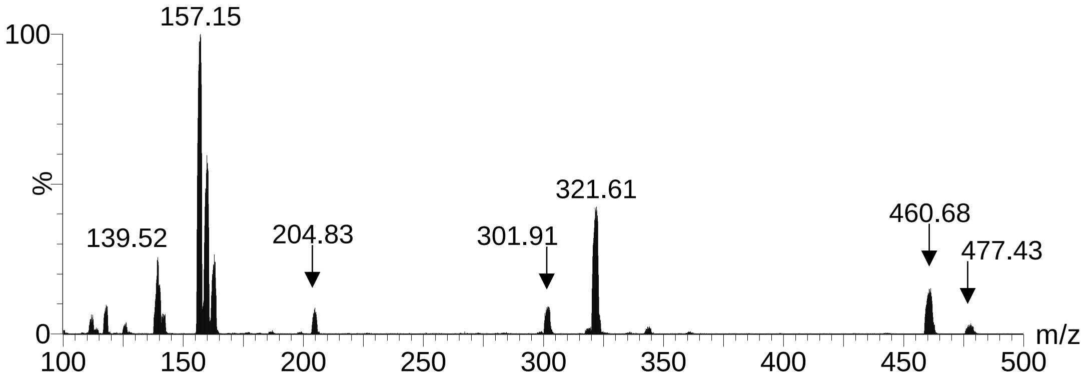

Parent/primary: mass to charge ratio of an ionized molecule before (parent) and after (daughter, highest response: primary daughter) fragmentation in the collision cell of a triple quadrupole mass spectrometer; ES+/ES-: positive/negative ion mode, ionisation by adding positively charged protons/negatively charged electrons; cone: cone voltage; CE: collision energy; eV: electron volt; inj.: injection volume; m/z: mass to charge ratio.

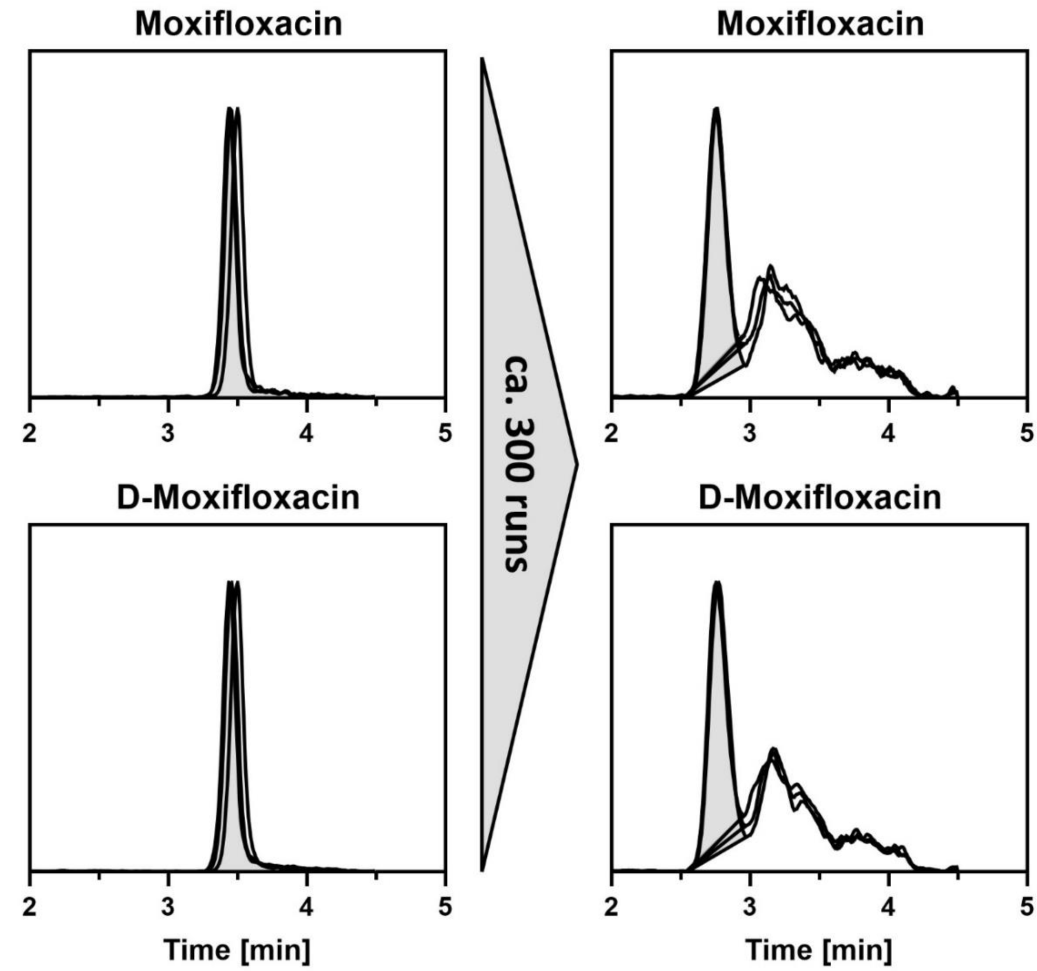

Figure S2. Peak tailing of moxifloxacin and D-moxifloxacin. Our column showed a fast ageing process, especially manifesting in moxifloxacin: 300 injections of matrix samples lead to a moxifloxacin peak shift of 45 seconds and considerable peak tailing. Deuterated moxifloxacin (D-moxifloxacin) as internal standard for moxifloxacin behaved identically and could compensate for the effect.

Table S8. Calibration curves.

	LLOQ - 1/10*ULOQ	LLOQ - ULOQ
Rifampicin	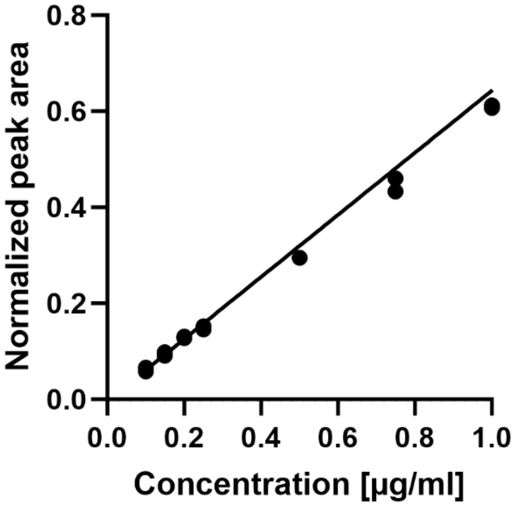	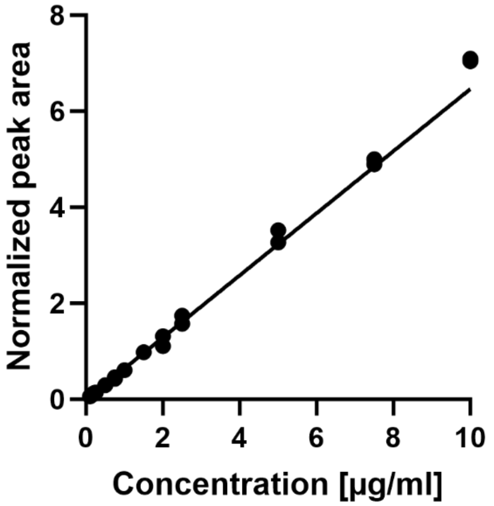
	Y-intercept = -0.003611; Slope = 0.6466	Y-intercept = -0.003611; Slope = 0.6466
Rifabutin	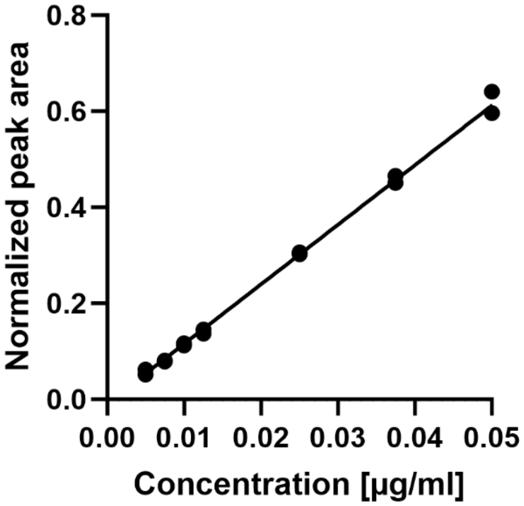	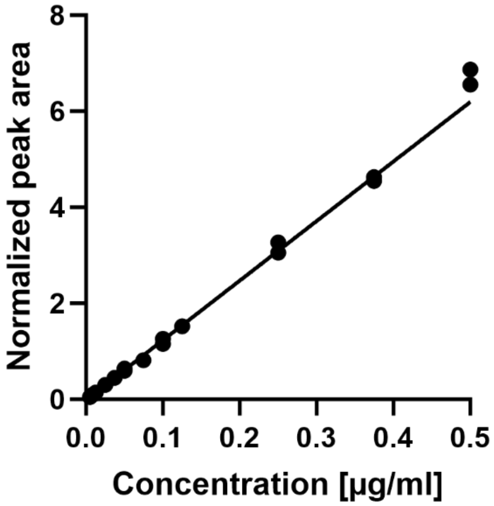
	Y-intercept = -0.008293; Slope = 12.41	Y-intercept = -0.008293; Slope = 12.41

Isoniazid	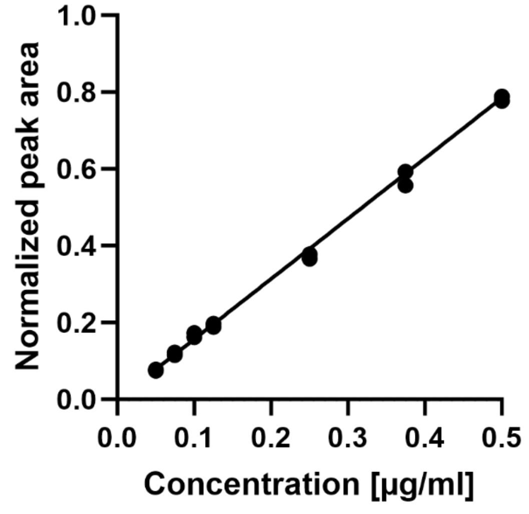<br>Y-intercept = -0.0001466; Slope = 1.569	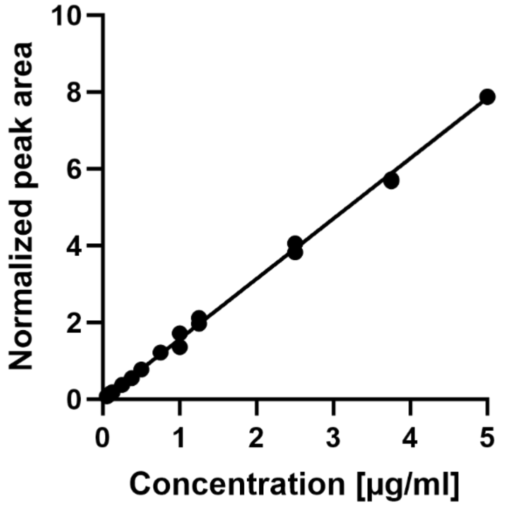<br>Y-intercept = -0.0001466; Slope = 1.569
Levofloxacin	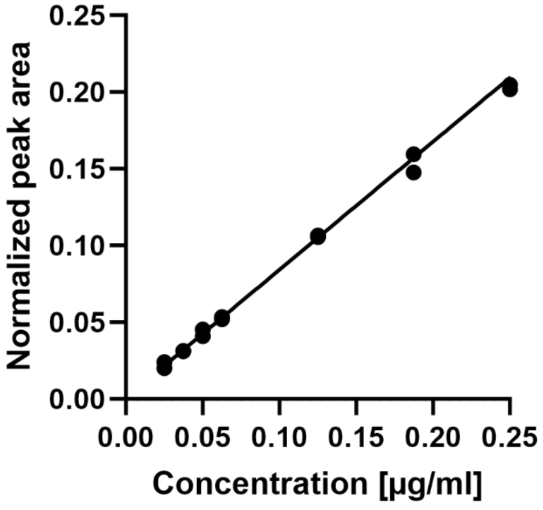<br>Y-intercept = 0.0008272; Slope = 0.8340	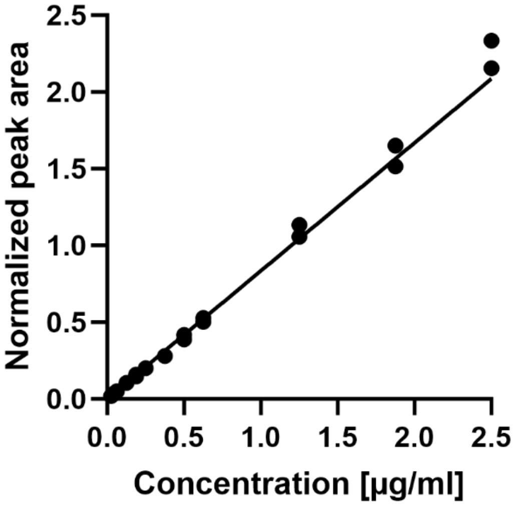<br>Y-intercept = 0.0008272; Slope = 0.8340
Moxifloxacin	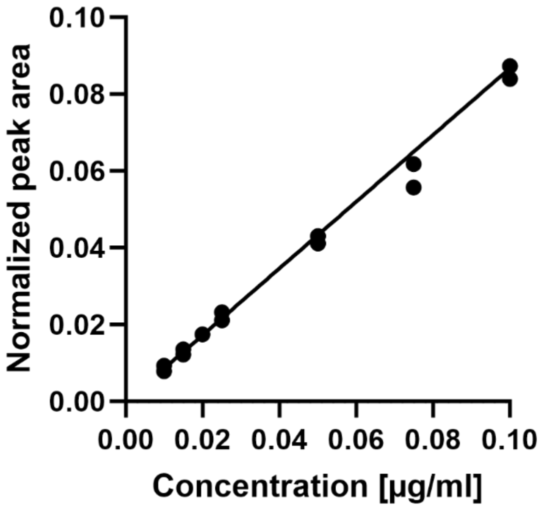<br>Y-intercept = -0.00009444; Slope = 0.8682	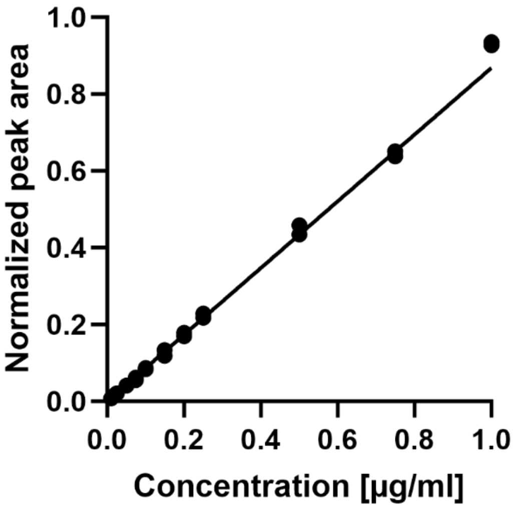<br>Y-intercept = -0.00009444; Slope = 0.8682

Bedaquiline

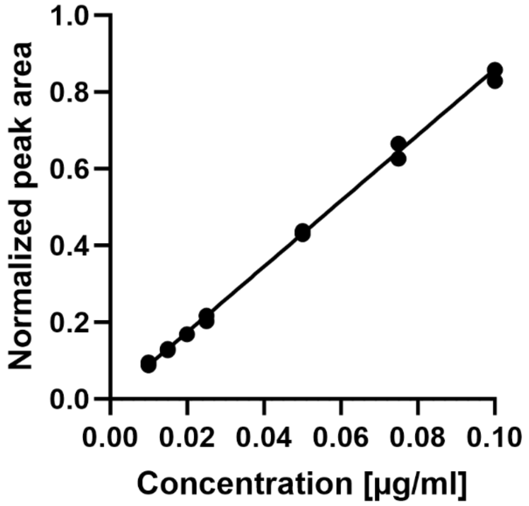

Y-intercept = 0.002452; Slope = 8.568

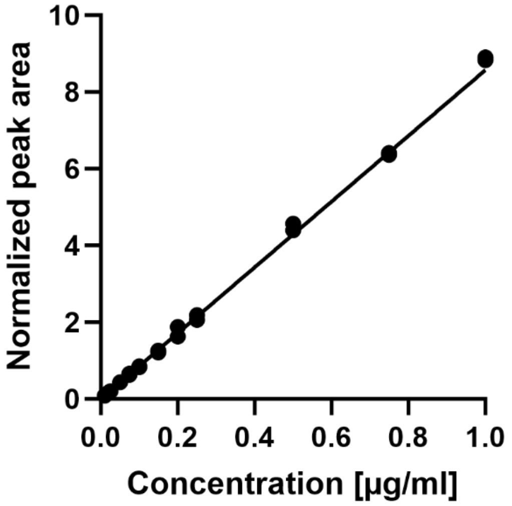

Y-intercept = 0.002452; Slope = 8.568

Linezolid

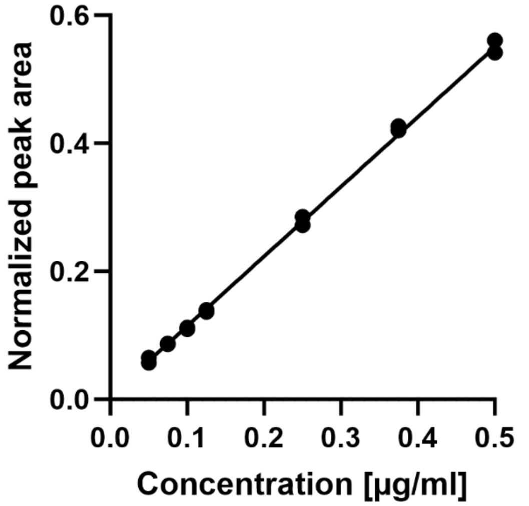

Y-intercept = 0,005359; Slope = 1,090

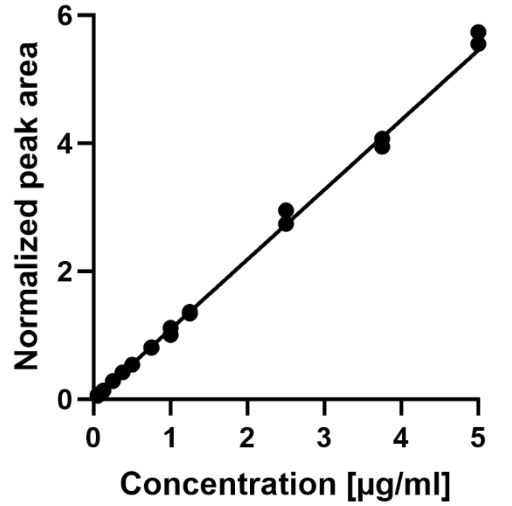

Y-intercept = 0.001363; Slope = 1.099

Pretomanid*

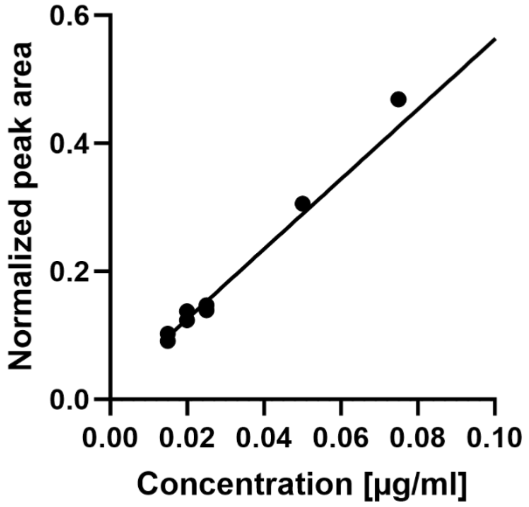

Y-intercept = 0.01636; Slope = 5.462

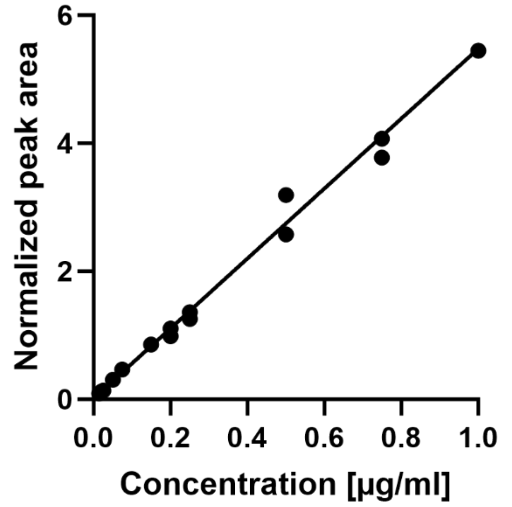

Y-intercept = 0.01636; Slope = 5.462

Clofazimine

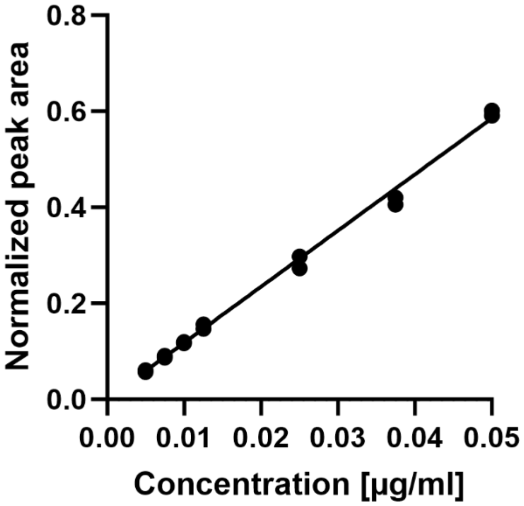

Y-intercept = 0.001363; Slope = 1.099

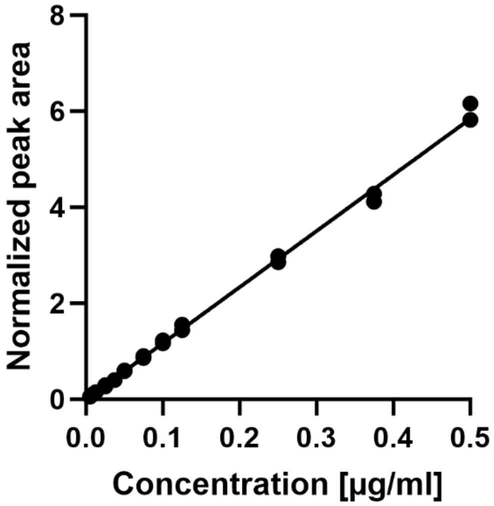

Y-intercept = 0.001363; Slope = 1.099

Cycloserine

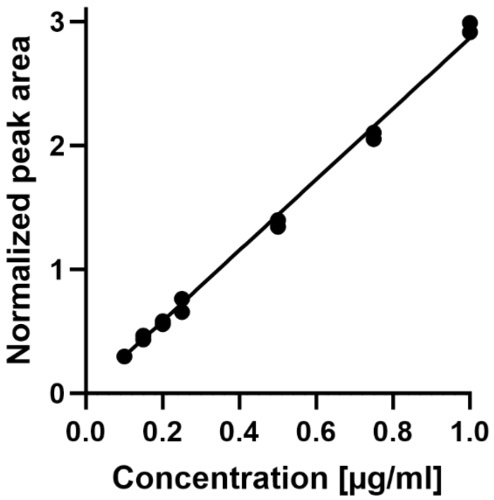

Y-intercept = 0.001147; Slope = 11.68

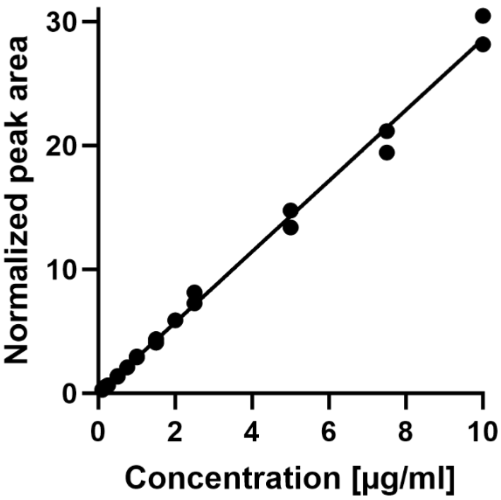

Y-intercept = 0.001147; Slope = 11.68

Ethambutol

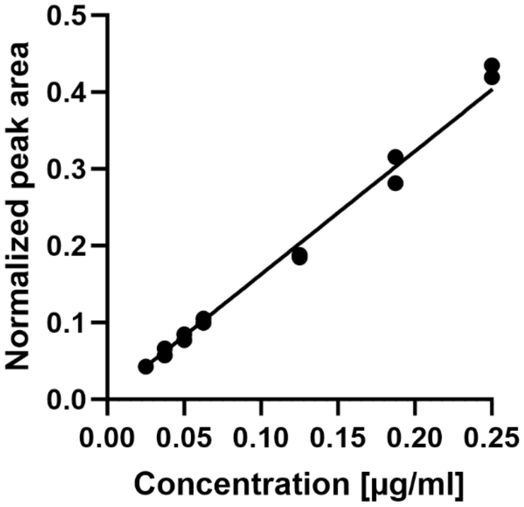

Y-intercept = -0.002435; Slope = 1.622

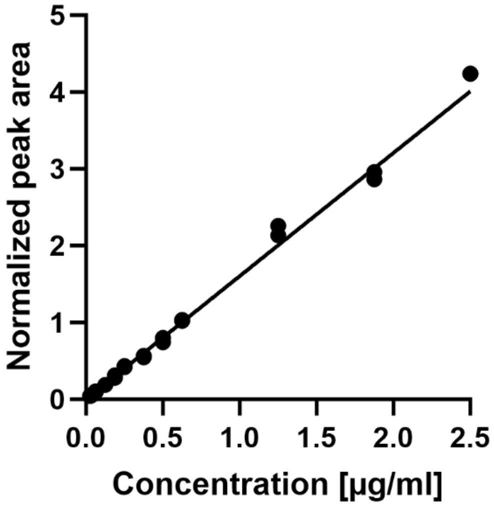

Y-intercept = -0.002435; Slope = 1.622

Delamanid	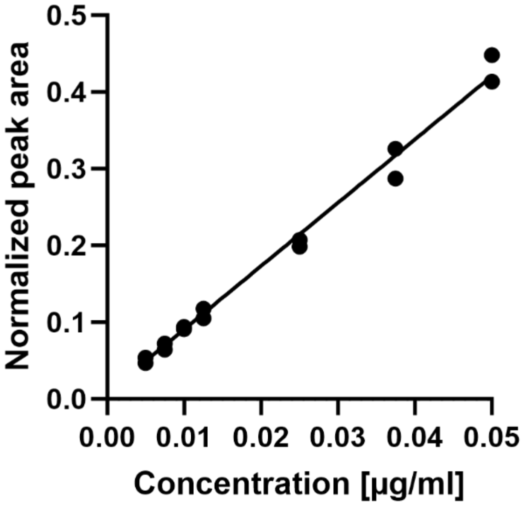 Y-intercept = 0.008393; Slope = 8.245	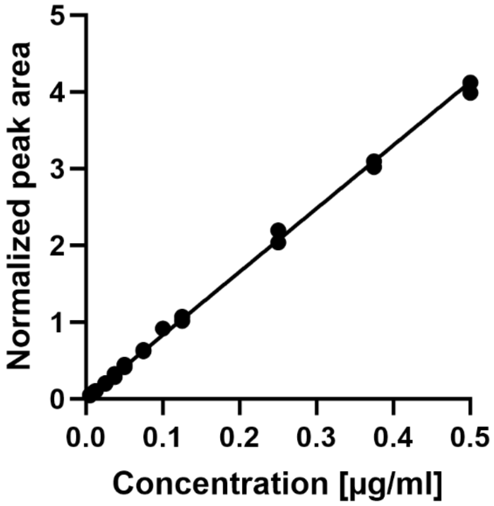 Y-intercept = 0.008393; Slope = 8.245
Pyrazinamide	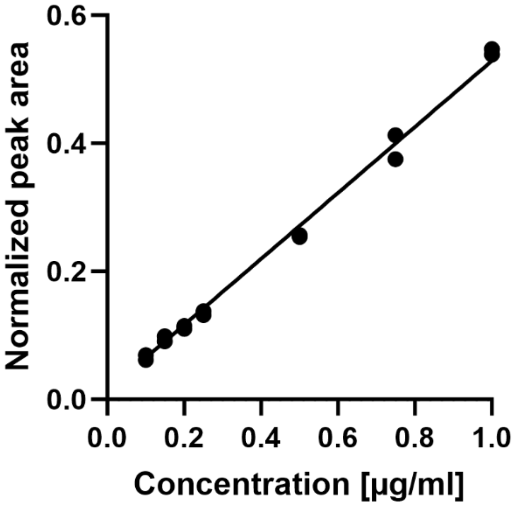 Y-intercept = 0.01331; Slope = 0.5154	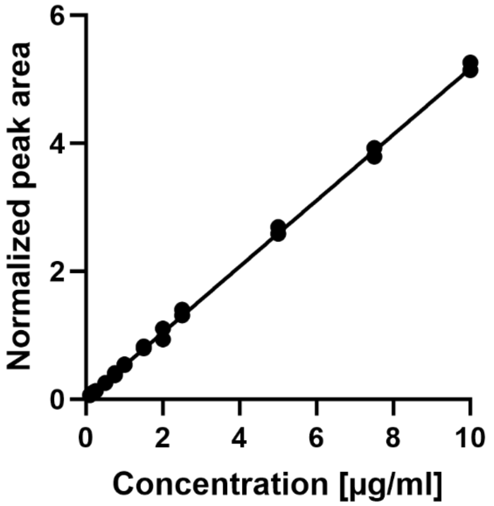 Y-intercept = 0.01331; Slope = 0.5154
Meropenem	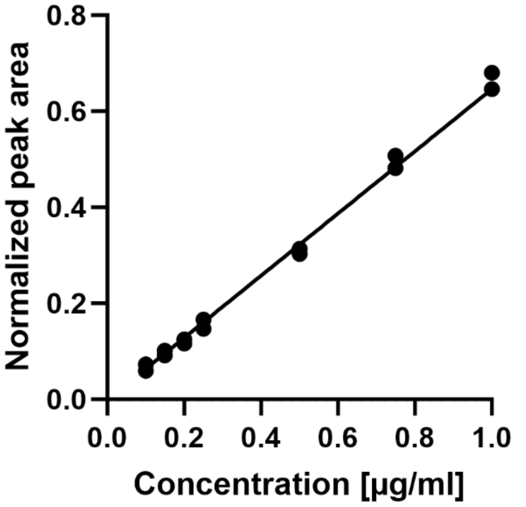 Y-intercept = 0.003047; Slope = 0.01174	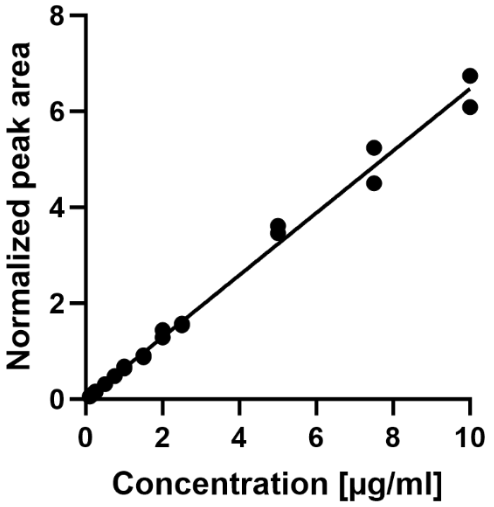 Y-intercept = 0.003047; Slope = 0.01174

Amikacin

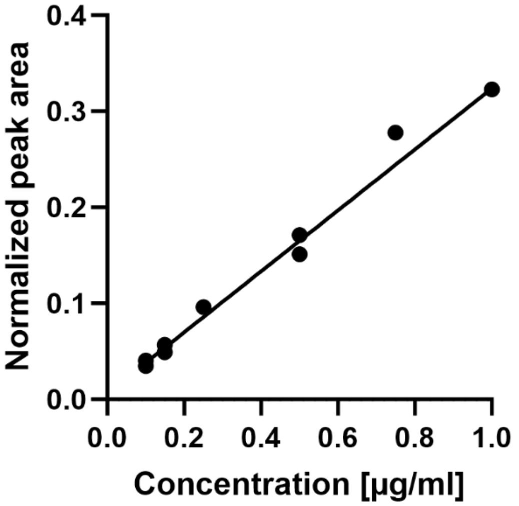

Y-intercept = 0.006386; Slope = 0.3174

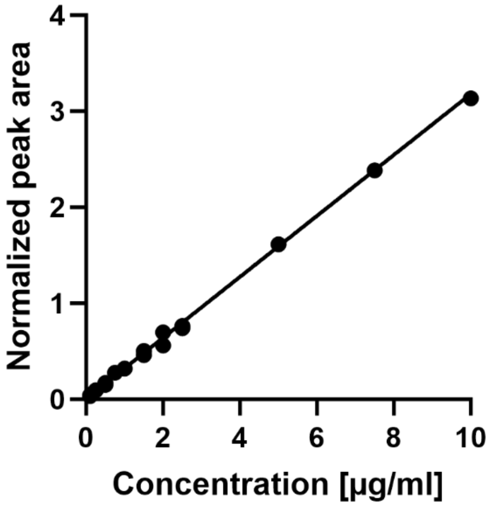

Y-intercept = 0.006386; Slope = 0.3174

Streptomycin[$]

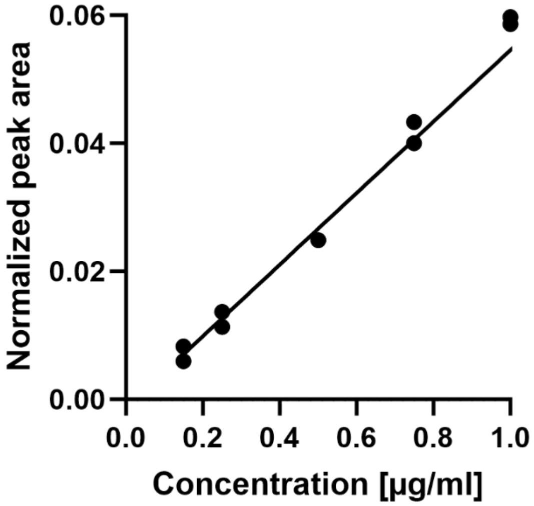

Y-intercept = -0.001298; Slope = 0.05585

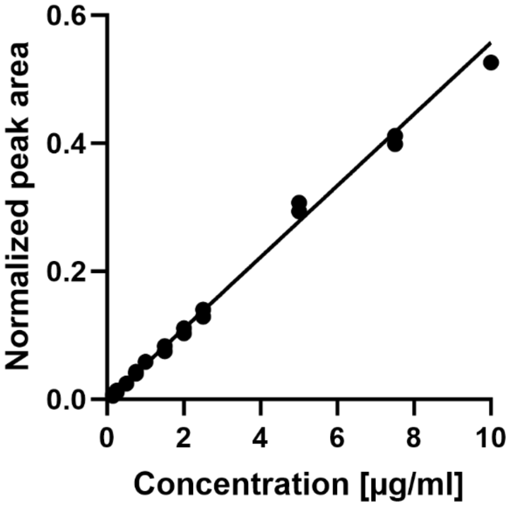

Y-intercept = -0.001298; Slope = 0.05585

Prothionamide

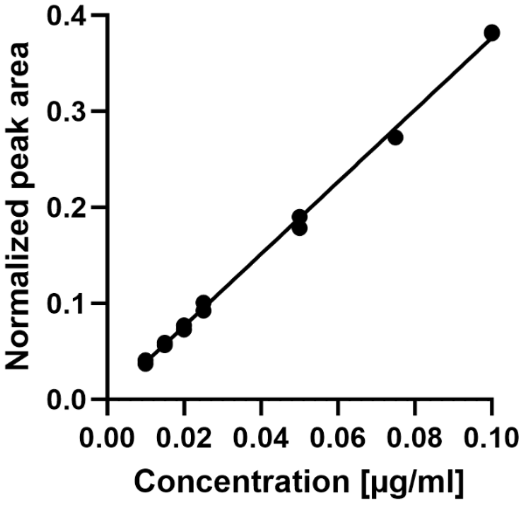

Y-intercept = 0.001311; Slope = 3.752

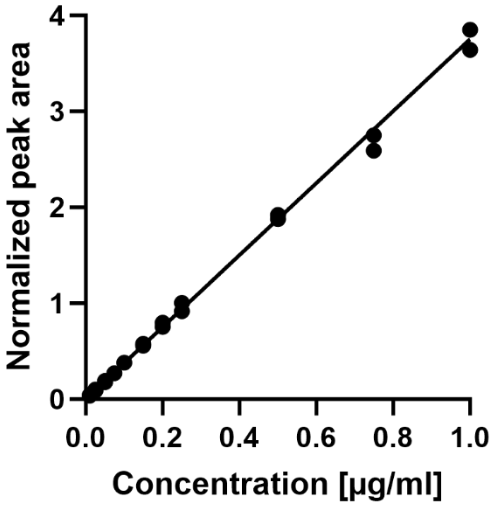

Y-intercept = 0.001311; Slope = 3.752

PAS

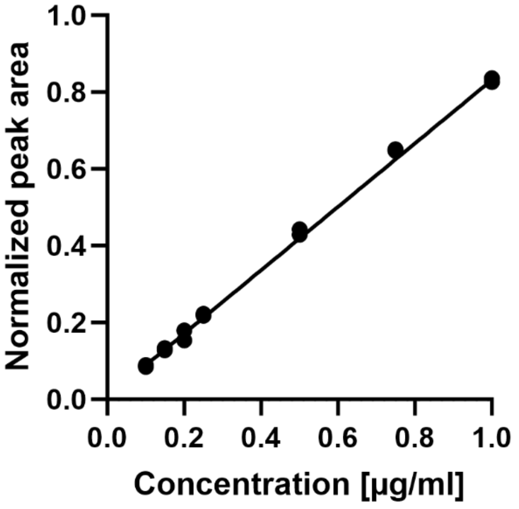

Y-intercept = 0.006192; Slope = 0.8252

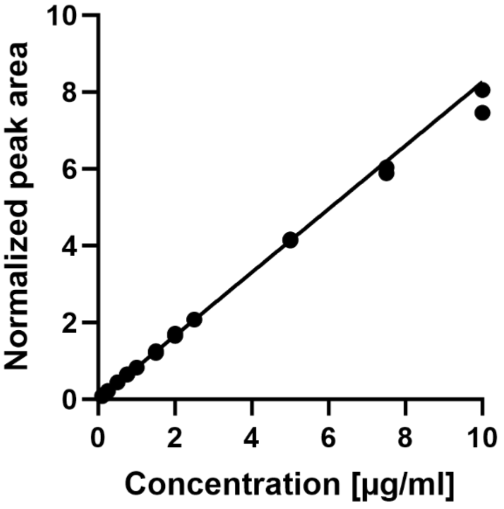

Y-intercept = 0.006192; Slope = 0.8252

Capreomycin IB$

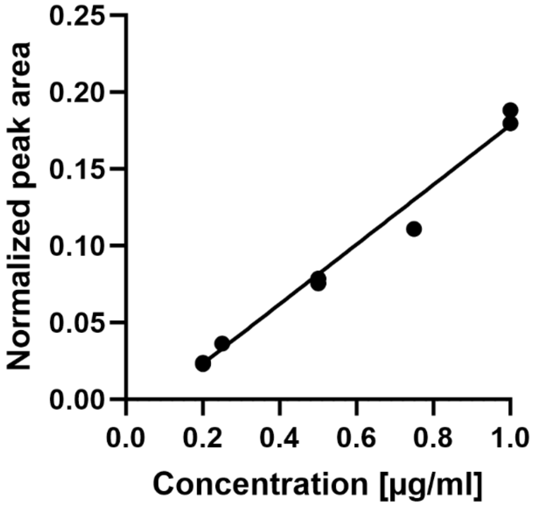

Y-intercept = -0.01564; Slope = 0.1942

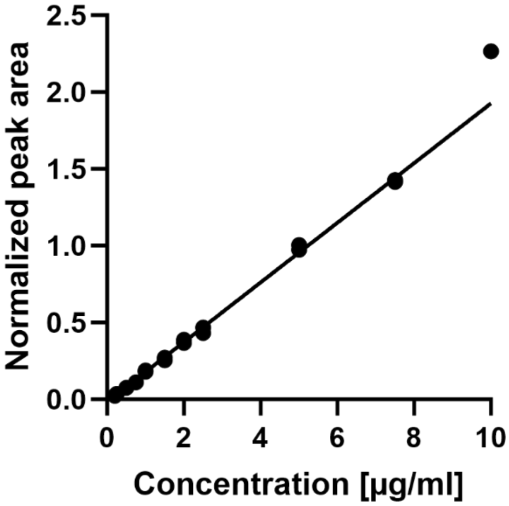

Y-intercept = -0.01564; Slope = 0.1942

Capreomycin IA$

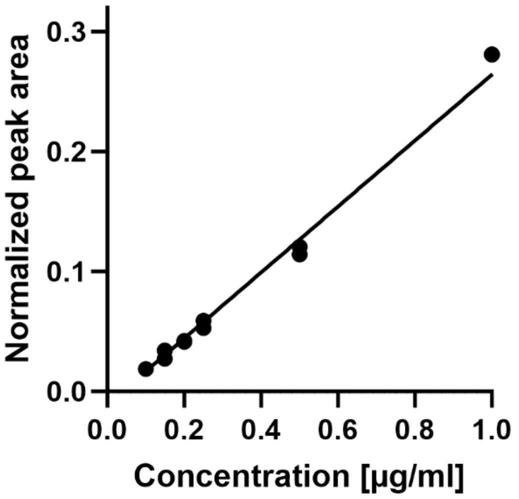

Y-intercept = -0.01073; Slope = 0.2749

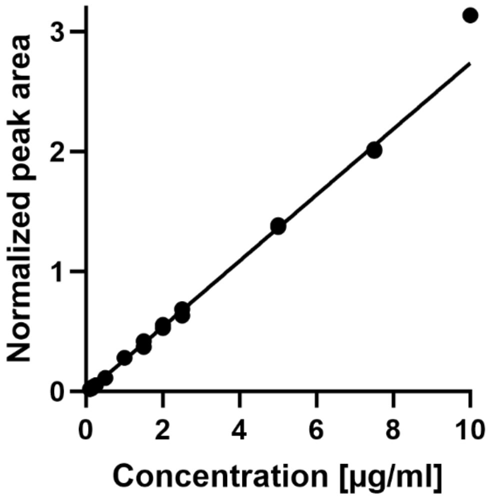

Y-intercept = -0.01073; Slope = 0.2749

Kanamycin

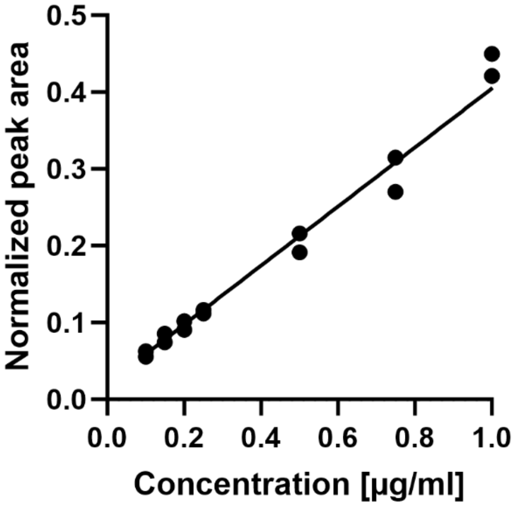

Y-intercept = 0.02053; Slope = 0.3842

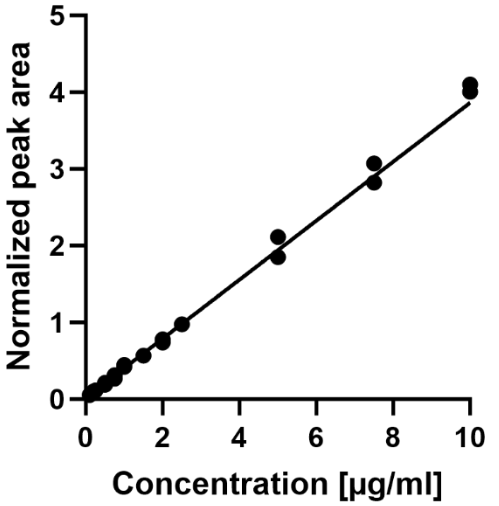

Y-intercept = 0.02053; Slope = 0.3842

X^2-weighted calibration curves of each analyte. Calibrators at 1, 0.75, 0.5, 0.25, 0.2, 0.15, 0.1, 0.075, 0.05, 0.025, 0.02, 0.015, and 0.01*$ULOQ_{target}$ were used and plotted in the range of LLOQ - 1/10*$ULOQ_{target}$ (left) and LLOQ - $ULOQ_{target}$ (right). $ULOQ_{target}$: targeted upper limit of quantification; LLOQ: lower limit of quantification; * normalised to D-delamanid; $ normalised to gentamicin.

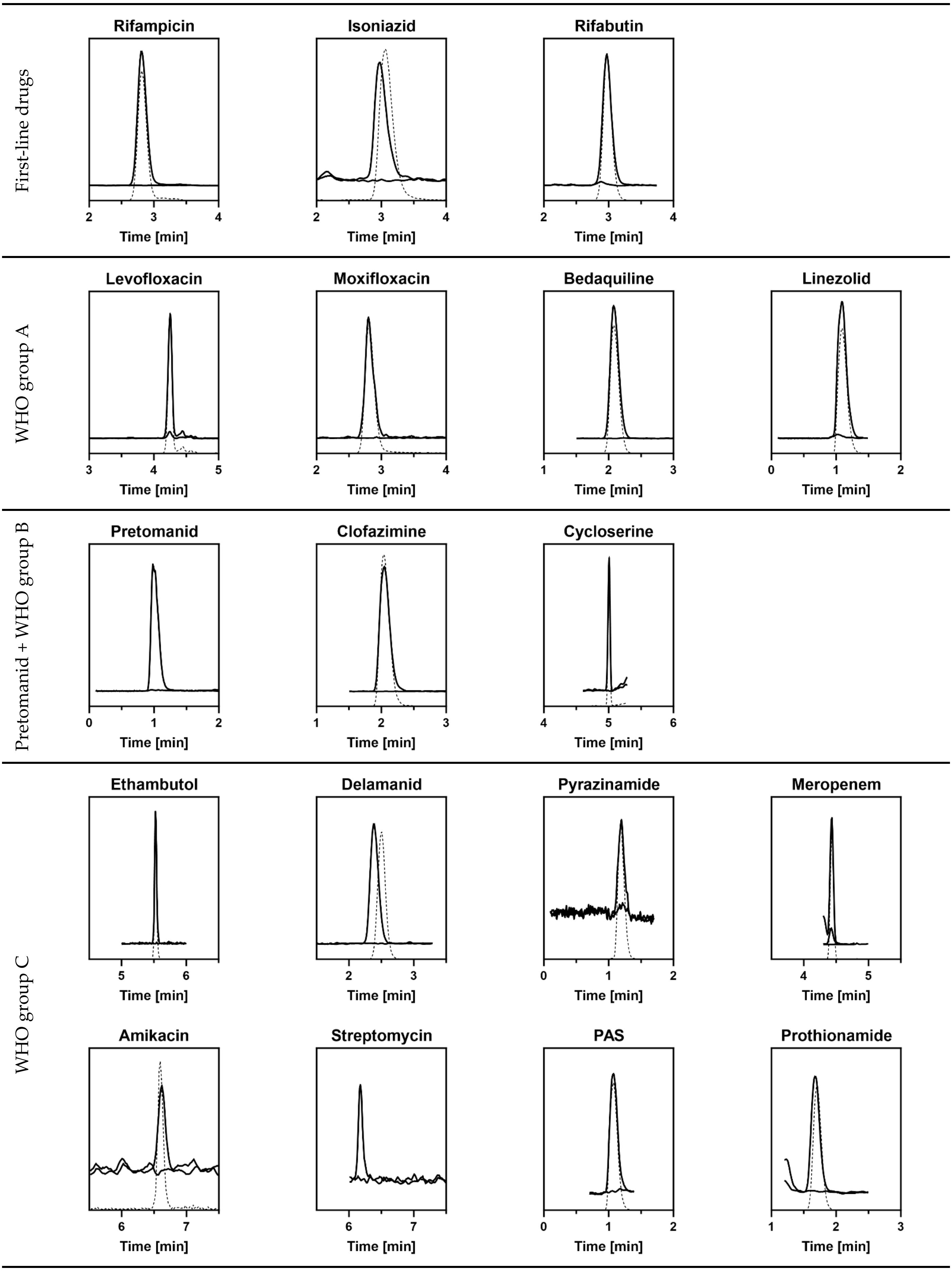

First-line drugs
Rifampicin
Isoniazid
Rifabutin
WHO group A
Levofloxacin
Moxifloxacin
Bedaquiline
Linezolid
Pretomanid + WHO group B
Pretomanid
Clofazimine
Cycloserine
WHO group C
Ethambutol
Delamanid
Pyrazinamide
Meropenem
Amikacin
Streptomycin
PAS
Prothionamide
Time [min]

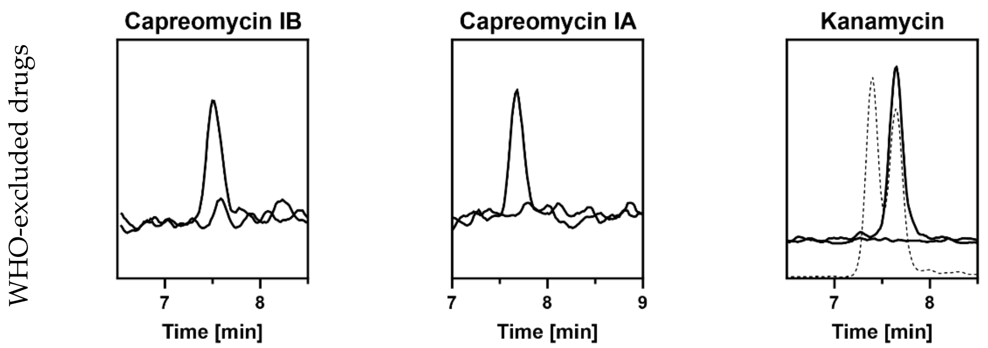

Figure S3. Selectivity: Analyte-, IS-, and matrix signal at LLOQ. The matrix signal did not exceed 20% of the analyte peak at the lower limit of quantification (LLOQ) in any of the analytes. All signals were recorded during calibration runs: The matrix signal was recorded in blank matrix samples before the first calibrator, analyte- and IS signal in the calibrator that represented the LLOQ.

Table S9. Specificity assessment by comparing analyte mass transitions with fragmentation mass spectra of common co-medications.

Drug	Formula	Accession number	platform	Ion mode	CE [eV]	Precursor	Fragment	% intensity	Source
**Cycloserine**			**LC-ESI-QQ**	**[M+H]+**	**10**	**102.88**	**75.03**		
**D-Cycloserine**			**LC-ESI-QQ**	**[M+H]+**	**10**	**106.22**	**78.85**		
Allopurinol	C5H4N4O	MSBNK-Fiocruz-FIO00457	LC-ESI-QTOF	[M+H]+	30	110.04	110.04	99.9	https://massbank.eu/MassBank
Allopurinol	C5H4N4O	MSBNK-Fiocruz-FIO00457	LC-ESI-QTOF	[M+H]+	30	110.04	110.04	99.9	https://massbank.eu/MassBank
**Pyrazinamide**			**LC-ESI-QQ**	**[M+H]+**	**20**	**123.68**	**78.88**		
**D-Pyrazinamide**			**LC-ESI-QQ**	**[M+H]+**	**20**	**127.21**	**82.85**		
Metformin	C4H11N5	MSBNK-Keio_Univ-KO003374	LC-ESI-QQ	[M+H]+	30	130.00	71.00	99.9	https://massbank.eu/MassBank
Metformin	C4H11N5	MSBNK-Keio_Univ-KO003375	LC-ESI-QQ	[M+H]+	30	130.00	71.10	99.9	https://massbank.eu/MassBank
**Isoniazid**			**LC-ESI-QQ**	**[M+H]+**	**25**	**137.68**	**78.50**		
**D-Isoniazid**			**LC-ESI-QQ**	**[M+H]+**	**25**	**141.72**	**82.60**		
Valproic acid	C22H25F2NO94	DB00403	LC-ESI-QQ	[M+H]+	25	145.00	144.41	100.0	https://go.drugbank.com/drugs
Dexamethasone	C22H29FO5	MSBNK-Fiocruz-FIO00437	LC-ESI-QTOF	[M+H]+	30	147.08	147.08	99.9	https://massbank.eu/MassBank
**PAS**			**LC-ESI-QQ**	**[M+H]+**	**25**	**153.64**	**91.17**		
Mesalazine	C7H7NO3	MSBNK-CASMI_2016-SM862104	LC-ESI-QFT	[M+H]+	35	154.05	91.05	1.0	https://massbank.eu/MassBank
**D-PAS**			**LC-ESI-QQ**	**[M+H]+**	**25**	**159.94**	**96.09**		
Pregabalin	C8H17NO2	MSBNK-Athens_Univ-AU166803	LC-ESI-QTOF	[M+H]+	30	160.13	142.12	99.9	https://massbank.eu/MassBank
Nicotine	C10H14N2	MSBNK-Eawag-EQ300802	LC-ESI-QFT	[M+H]+	30	163.12	94.06	0.3	https://massbank.eu/MassBank

N-Acetyl-L-cysteine	C5H9NO3S	MSBNK-RIKEN_ReSpect-PS030401	LC-ESI-QQ	[M+H]+	30	164.12	122.00	99.9	https://massbank.eu/MassBank
Levetiracetam	C8H14N2O2	MSBNK-Athens_Univ-AU160503	LC-ESI-QTOF	[M+H]+	30	171.11	126.09	99.9	https://massbank.eu/MassBank
Gabapentin	C9H17NO2	MSBNK-Athens_Univ-AU167803	LC-ESI-QTOF	[M+H]+	30	172.13	137.10	99.9	https://massbank.eu/MassBank
**Prothionamide**			**LC-ESI-QQ**	**[M+H]+**	**25**	**180.57**	**120.94**		
**D-Prothionamide**			**LC-ESI-QQ**	**[M+H]+**	**25**	**187.58**	**127.38**		
N-Acetyl mesalazine	C9H9NO4	MSBNK-Athens_Univ-AU272503	LC-ESI-QTOF	[M+H]+	30	196.06	136.04	99.9	https://massbank.eu/MassBank
**Clavulanic acid**			**LC-ESI-QQ**	**[M+H]+**	**10**	**198.00**	**136.00**		
**Ethambutol**			**LC-ESI-QQ**	**[M+H]+**	**25**	**204.78**	**44.29**		
Ibuprofen	C13H18O2	MSBNK-RIKEN_ReSpect-PS025302	LC-ESI-QQ	[M+H]+	20	207.17	81.00	3.0	https://massbank.eu/MassBank
**D-Ethambutol**			**LC-ESI-QQ**	**[M+H]+**	**25**	**208.78**	**48.29**		
Clonidine	C9H9Cl2N3	MSBNK-Keio_Univ-KO002629	LC-ESI-QQ	[M+H]+	30	230.00	230.20	99.9	https://massbank.eu/MassBank
Naproxen	C14H14O3	MSBNK-Athens_Univ-AU265103	LC-ESI-QTOF	[M+H]+	30	231.10	185.10	99.9	https://massbank.eu/MassBank
Salbutamol	C13H21NO3	MSBNK-Athens_Univ-AU110002	LC-ESI-QTOF	[M+H]+	ramp 19.4-29.2	240.16	148.08	99.9	https://massbank.eu/MassBank
Pyridoxal phosphate	C8H10NO6P	DB00225	LC-ESI-QQ	[M+H]+	25	248.00	94.000 100.000	100.0	https://go.drugbank.com/drugs
Emtricitabine	C8H10FN3O3S	MSBNK-Eawag-EQ310652	LC-ESI-QFT	[M+H]+	30	248.05	130.04	99.9	https://massbank.eu/MassBank
Sulfamethoxazole	C10H11N3O3S	MSBNK-HBM4EU-HB002604	LC-ESI-QTOF	[M+H]+	30	254.06	65.04	99.9	https://massbank.eu/MassBank
Triamterene	C12H11N7	MSBNK-Athens_Univ-AU110903	LC-ESI-QTOF	[M+H]+	30	254.11	254.11	99.9	https://massbank.eu/MassBank

Tramadol	C16H25NO2	MSBNK-Athens_Univ-AU111703	LC-ESI-QTOF	[M+H]+	30	264.20	159.08	99.9	https://massbank.eu/MassBank
Thiamine	C12H17N4OS+	MSBNK-Keio_Univ-KO004188	LC-ESI-QQ	[M+H]+	30	266.00	122.20	99.9	https://massbank.eu/MassBank
Mirtazapine	C17H19N3	MSBNK-Athens_Univ-AU155109	LC-ESI-QTOF	[M+H]+	30	266.17	195.09	99.9	https://massbank.eu/MassBank
Nevirapine	C15H14N4O	DB00268	predicted	[M+H]+	20	267.00	267.12	51.7	https://go.drugbank.com/drugs
Metoprolol	C15H25NO3	MSBNK-Athens_Univ-AU110703	LC-ESI-QTOF	[M+H]+	30	268.19	133.06	99.9	https://massbank.eu/MassBank
Moclobemide	C13H17ClN2O2	MSBNK-Athens_Univ-AU227903	LC-ESI-QTOF	[M+H]+	30	269.11	182.04	99.9	https://massbank.eu/MassBank
17-alpha-Estradiol	C18H24O2	MSBNK-Athens_Univ-AU281703	LC-ESI-QTOF	[M+H]+	30	273.18	255.17	99.9	https://massbank.eu/MassBank
17-beta-Estradiol	C18H24O2	MSBNK-Athens_Univ-AU279703	LC-ESI-QTOF	[M+H]+	30	273.18	255.17	99.9	https://massbank.eu/MassBank
Amitriptyline	C20H23N	MSBNK-Athens_Univ-AU150403	LC-ESI-QTOF	[M+H]+	30	278.19	191.08	99.9	https://massbank.eu/MassBank
Doxepine	C19H21NO	MSBNK-Athens_Univ-AU153206	LC-ESI-QTOF	[M+H]+	ramp 20.9-31.4	280.17	280.17	99.9	https://massbank.eu/MassBank
Phenprocoumon	C18H16O33	DB00976	predicted	[M+H]+	20	281.00	281.12	37.5	https://go.drugbank.com/drugs
Risedronic acid	C7H11NO7P7	DB00889	predicted	[M+H]+	40	284.00	80.97	30.8	https://go.drugbank.com/drugs
Diazepam	C16H13ClN2O	MSBNK-Athens_Univ-AU160803	LC-ESI-QTOF	[M+H]+	30	285.08	285.08	99.9	https://massbank.eu/MassBank
Probenecid	C13H19NO4S	DB01067	predicted	[M+H]+	40	286.00	43.05	68.3	https://go.drugbank.com/drugs
Morphine	C17H19NO3	MSBNK-Athens_Univ-AU158003	LC-ESI-QTOF	[M+H]+	30	286.14	286.15	99.9	https://massbank.eu/MassBank
Oxazepam	C15H11ClN2O2	MSBNK-Athens_Univ-AU268003	LC-ESI-QTOF	[M+H]+	30	287.06	241.05	99.9	https://massbank.eu/MassBank

Tenofovir	C9H14N5O4P	MSBNK-Eawag-EQ310502	LC-ESI-QFT	[M+H]+	30	288.09	288.09	99.9	https://massbank.eu/MassBank
Atropine	C17H23NO3	MSBNK-Keio_Univ-KO002225	LC-ESI-QQ	[M+H]+	30	290.00	124.20	99.9	https://massbank.eu/MassBank
Trimethoprim	C14H18N4O3	MSBNK-Keio_Univ-KO004154	LC-ESI-QQ	[M+H]+	30	291.00	230.20	99.9	https://massbank.eu/MassBank
Dimetindene	C20H24N24	DB08823	predicted	[M+H]+	20	293.00	248.14	17.2	https://go.drugbank.com/drugs
Diclofenac	C14H11Cl2NO2	MSBNK-Keio_Univ-KO002783	LC-ESI-QQ	[M+H]+	30	296.00	215.20	99.9	https://massbank.eu/MassBank
N4-Acetyl-Sulfamethoxazole	C12H13N3O4S	MSBNK-Athens_Univ-AU201403	LC-ESI-QTOF	[M+H]+	30	296.07	134.06	99.9	https://massbank.eu/MassBank
17a-Ethynylestradiol	C20H24O2	MSBNK-Eawag-EQ306502	LC-ESI-QFT	[M+H]+	30	297.18	107.05	99.9	https://massbank.eu/MassBank
Hydrochlorothiazide	C7H8ClN3O4S69	DB01066	LC-ESI-QQ	[M+H]+	25	298.00	156.44	100.0	https://go.drugbank.com/drugs
Norethindrone	C20H26O2	MSBNK-Athens_Univ-AU279803	LC-ESI-QTOF	[M+H]+	30	299.20	231.17	99.9	https://massbank.eu/MassBank
Codeine	C18H21NO3	MSBNK-IPB_Halle-PB001606	LC-ESI-QQ	[M+H]+	35	300.00	300.00	99.9	https://massbank.eu/MassBank
Metoclopramide	C14H22ClN3O2	MSBNK-Athens_Univ-AU228903	LC-ESI-QTOF	[M+H]+	30	300.15	227.06	99.9	https://massbank.eu/MassBank
Tazobactam	C10H12N4O5S	DB01622	predicted	[M+H]+	20	301.00	232.03	36.8	https://go.drugbank.com/drugs
Dihydrocodeine	C18H23NO3	MSBNK-Athens_Univ-AU217803	LC-ESI-QTOF	[M+H]+	30	302.18	302.18	99.9	https://massbank.eu/MassBank
Scopolamine	C17H21NO4	MSBNK-Keio_Univ-KO004014	LC-ESI-QQ	[M+H]+	30	304.00	138.20	99.9	https://massbank.eu/MassBank
Fenoterol	C17H21NO4	MSBNK-Univ_Connecticut-CO000198	LC-ESI-QTOF	[M+H]+	30	304.16	107.05	99.9	https://massbank.eu/MassBank
Methadone	C21H27NO	MSBNK-Athens_Univ-AU162803	LC-ESI-QTOF	[M+H]+	30	310.22	265.16	99.9	https://massbank.eu/MassBank
Biperiden	C21H29NO	DB00824	predicted	[M+H]+	20	312.00	98.10	38.2	https://go.drugbank.com/drugs

Olanzapine	C17H20N4S	MSBNK-Athens_Univ-AU151703	LC-ESI-QTOF	[M+H]+	30	313.15	169.08	1.9	https://massbank.eu/MassBank
Ranitidine	C13H22N4O3S	MSBNK-Keio_Univ-KO003964	LC-ESI-QQ	[M+H]+	30	315.00	176.10	99.9	https://massbank.eu/MassBank
Oxycodone	C18H21NO4	MSBNK-IPB_Halle-PB002102	LC-ESI-QQ	[M+H]+	30	316.00	298.00	99.9	https://massbank.eu/MassBank
Tamsulosin	C15H10Cl2N2O2	MSBNK-Athens_Univ-AU151203	LC-ESI-QTOF	[M+H]+	30	321.02	275.01	99.9	https://massbank.eu/MassBank
Clopidogrel	C16H16ClNO2S	MSBNK-Athens_Univ-AU228403	LC-ESI-QTOF	[M+H]+	30	322.07	184.05	99.9	https://massbank.eu/MassBank
Citalopram	C20H21FN2O	MSBNK-Athens_Univ-AU151303	LC-ESI-QTOF	[M+H]+	30	325.17	262.10	99.9	https://massbank.eu/MassBank
Midazolam	C18H13ClFN3	MSBNK-Athens_Univ-AU155903	LC-ESI-QTOF	[M+H]+	30	326.09	326.09	99.9	https://massbank.eu/MassBank
**Capreomycin IB**			**LC-ESI-QQ**	**[M+H]+**	**20**	**326.21**	**70.18**		
Bisoprolol	C18H31NO4	MSBNK-Athens_Univ-AU267703	LC-ESI-QTOF	[M+H]+	30	326.23	115.05	1.3	https://massbank.eu/MassBank
Levomepromazine	C19H24N2OS	MSBNK-Keio_Univ-KO003494	LC-ESI-QQ	[M+H]+	30	329.00	100.20	99.9	https://massbank.eu/MassBank
Furosemide	C12H11ClN2O5S	MSBNK-Athens_Univ-AU269203	LC-ESI-QTOF	[M+H]+	30	331.02	149.06	99.9	https://massbank.eu/MassBank
**Capreomycin IA**			**LC-ESI-QQ**	**[M+H]+**	**20**	**334.27**	**70.24**		
**Linezolid**			**LC-ESI-QQ**	**[M+H]+**	**25**	**337.17**	**195.20**		
Medrogestone	C23H32O32	DB09154	predicted	[M+H]+	20	341.00	341.25	13.4	https://go.drugbank.com/drugs
Formoterol	C19H24N2O51	DB01030	predicted	[M+H]+	40	345.00	149.10	18.6	https://go.drugbank.com/drugs
**D-Linezolid**			**LC-ESI-QQ**	**[M+H]+**	**25**	**345.10**	**203.20**		
Omeprazole	C17H19N3O3S	MSBNK-Athens_Univ-AU111303	LC-ESI-QTOF	[M+H]+	30	346.12	200.06	2.4	https://massbank.eu/MassBank

Indomethacin	C19H16ClNO4	MSBNK-Athens_Univ-AU271403	LC-ESI-QTOF	[M+H]+	30	358.08	174.09	5.8	https://massbank.eu/MassBank
**Pretomanid**			**LC-ESI-QQ**	**[M+H]+**	**25**	**359.00**	**174.82**		
Prednisone	C21H26O5	MSBNK-Athens_Univ-AU240103	LC-ESI-QTOF	[M+H]+	30	359.19	147.08	99.9	https://massbank.eu/MassBank
Prednisolone	C21H28O5	MSBNK-Athens_Univ-AU239803	LC-ESI-QTOF	[M+H]+	30	361.20	261.13	3.6	https://massbank.eu/MassBank
Scopolamine-N-butyl	C21H30NO4+	MSBNK-Athens_Univ-AU251503	LC-ESI-QTOF	[M+H]+	30	361.22	360.22	99.9	https://massbank.eu/MassBank
**Levofloxacin**			**LC-ESI-QQ**	**[M+H]+**	**30**	**361.82**	**261.17**		
Bisacodyl	C22H19NO11	DB09027	predicted	[M+H]+	20	362.00	260.11	5.3	https://go.drugbank.com/drugs
Bisacodyl	C22H19NO46	DB09062	predicted	[M+H]+	40	362.00	260.11	10.5	https://go.drugbank.com/drugs
Hydrocortisone	C21H30O5	MSBNK-Univ_Connecticut-CO000223	LC-ESI-QTOF	[M+H]+	30	363.22	261.16	2.0	https://massbank.eu/MassBank
**D-Levofloxacin**			**LC-ESI-QQ**	**[M+H]+**	**30**	**366.04**	**261.04**		
Haloperidol	C21H23ClFNO2	MSBNK-Athens_Univ-AU112203	LC-ESI-QTOF	[M+H]+	30	376.15	165.07	99.9	https://massbank.eu/MassBank
Celecoxib	C17H14F3N3O2S	MSBNK-Athens_Univ-AU235603	LC-ESI-QTOF	[M+H]+	30	382.08	184.04	0.7	https://massbank.eu/MassBank
**Meropenem**			**LC-ESI-QQ**	**[M+H]+**	**30**	**383.95**	**68.04**		
Pantoprazole	C16H15F2N3O4S	MSBNK-Athens_Univ-AU227403	LC-ESI-QTOF	[M+H]+	30	384.08	122.06	0.8	https://massbank.eu/MassBank
Zopiclone	C17H17ClN6O3	MSBNK-Eawag-EQ364302	LC-ESI-QFT	[M+H]+	30	389.11	84.07	0.1	https://massbank.eu/MassBank
**D-Meropenem**			**LC-ESI-QQ**	**[M+H]+**	**30**	**389.96**	**68.06**		
**Moxifloxacin**			**LC-ESI-QQ**	**[M+H]+**	**35**	**401.67**	**364.21**		
Nebivolol	C22H25F2NO34	DB04891	predicted	[M+H]+	20	406.00	406.18	4.5	https://go.drugbank.com/drugs
**D-Moxifloxacin**			**LC-ESI-QQ**	**[M+H]+**	**35**	**407.30**	**369.08**		

Sitagliptin	C16H15F6N5O	MSBNK-Athens_Univ-AU225703	LC-ESI-QTOF	[M+H]+	30	408.13	391.10	9.9	https://massbank.eu/MassBank
Amlodipine	C20H25ClN2O5	MSBNK-Athens_Univ-AU154406	LC-ESI-QTOF	[M+H]+	ramp 23.8-35.7	409.15	238.06	99.9	https://massbank.eu/MassBank
Ezetemibe	C24H21F2NO32	DB01002	predicted	[M+H]+	20	410.00	392.15	14.5	https://go.drugbank.com/drugs
Risperidone	C23H27FN4O2	MSBNK-Athens_Univ-AU150503	LC-ESI-QTOF	[M+H]+	30	411.22	191.12	99.9	https://massbank.eu/MassBank
Ramipril	C23H32N2O26	DB00199	predicted	[M+H]+	20	417.00	234.15	22.8	https://go.drugbank.com/drugs
Spironolactone	C24H32O4S	DB00477	predicted	[M+H]+	40	417.00	329.19	2.1	https://go.drugbank.com/drugs
Simvastatin	C25H38O5	MSBNK-Athens_Univ-AU112803	LC-ESI-QTOF	[M+H]+	30	419.28	199.15	99.9	https://massbank.eu/MassBank
Dolutegravir	C20H19F2N3O11	DB08936	predicted	[M+H]+	20	420.00	142.05	31.5	https://go.drugbank.com/drugs
Candesartan	C24H20N6O3	MSBNK-Athens_Univ-AU213703	LC-ESI-QTOF	[M+H]+	30	441.17	207.09	99.9	https://massbank.eu/MassBank
Folic acid	C19H19N7O6	MSBNK-Washington_State_Univ-BML00977	LC-ESI-QTOF	[M+H]+	CE40	442.15	176.06	99.9	https://massbank.eu/MassBank
Folic acid	C19H19N7O6	MSBNK-Univ_Connecticut-CO000203	LC-ESI-QTOF	[M+H]+	30	442.15	295.10	99.9	https://massbank.eu/MassBank
Glycoursodeoxycholic acid	0	MSBNK-BGC_Munich-RP005503	LC-ESI-QTOF	[M+H]+	40	450.32	76.04	99.9	https://massbank.eu/MassBank
Buprenorphine	C29H41NO4	MSBNK-Univ_Connecticut-CO000093	LC-ESI-QTOF	[M+H]+	30	468.31	468.31	99.9	https://massbank.eu/MassBank
**Clofazimine**			**LC-ESI-QQ**	**[M+H]+**	**45**	**471.61**	**395.25**		
Loperamide	C29H33ClN2O2	MSBNK-Keio_Univ-KO003324	LC-ESI-QQ	[M+H]+	30	477.00	149.40	0.1	https://massbank.eu/MassBank
**Gentamicin**			**LC-ESI-QQ**	**[M+H]+**	**20**	**477.43**	**157.15**		
**D-Clofazimine**			**LC-ESI-QQ**	**[M+H]+**	**45**	**480.00**	**396.15**		

**Kanamycin**			**LC-ESI-QQ**	**[M+H]+**	**25**	**484.46**	**162.48**		
**D-Kanamycin**			**LC-ESI-QQ**	**[M+H]+**	**25**	**490.63**	**162.52**		
Fluticasone propionate	C25H31F3O5S	MSBNK-Athens_Univ-AU283103	LC-ESI-QTOF	[M+H]+	30	501.19	293.15	99.9	https://massbank.eu/MassBank
Piperacillin	C23H27N5O7S	MSBNK-Keio_Univ-KO003720	LC-ESI-QQ	[M+H]+	30	518.00	204.60	99.9	https://massbank.eu/MassBank
**Delamanid**			**LC-ESI-QQ**	**[M+H]+**	**25**	**534.37**	**352.07**		
Aprepitant	C23H21F7N4O24	DB00694	predicted	[M+H]+	20	535.00	377.08	0.7	https://go.drugbank.com/drugs
**D-Delamanid**			**LC-ESI-QQ**	**[M+H]+**	**25**	**538.30**	**356.07**		
**Bedaquiline**			**LC-ESI-QQ**	**[M+H]+**	**35**	**555.14**	**58.16**		
Atorvastatin	C33H35FN2O5	MSBNK-Athens_Univ-AU112903	LC-ESI-QTOF	[M+H]+	30	559.26	440.23	99.9	https://massbank.eu/MassBank
**D-Bedaquiline**			**LC-ESI-QQ**	**[M+H]+**	**35**	**561.13**	**64.13**		
**Streptomycin**			**LC-ESI-QQ**	**[M+H]+**	**35**	**582.03**	**263.10**		
**Amikacin**			**LC-ESI-QQ**	**[M+H]+**	**30**	**586.06**	**163.07**		
**D-Amikacin**			**LC-ESI-QQ**	**[M+H]+**	**30**	**590.11**	**162.52**		
Clarithromycin	C38H69NO13	MSBNK-Athens_Univ-AU105303	LC-ESI-QTOF	[M+H]+	30	748.48	158.12	99.9	https://massbank.eu/MassBank
L-Thyroxine	C15H11I4NO4	MSBNK-Athens_Univ-AU274306	LC-ESI-QTOF	[M+H]+	ramp 31.4-47.1	777.69	731.69	99.9	https://massbank.eu/MassBank
**Rifampicin**			**LC-ESI-QQ**	**[M+H]+**	**20**	**823.46**	**791.01**		
**D-Rifampicin**			**LC-ESI-QQ**	**[M+H]+**	**20**	**830.25**	**798.69**		
**Rifabutin**			**LC-ESI-QQ**	**[M+H]+**	**35**	**847.02**	**815.52**		
**D-Rifabutin**			**LC-ESI-QQ**	**[M+H]+**	**35**	**852.59**	**821.16**		
Nystatin	C47H75NO17	MSBNK-Eawag-EQ314002	LC-ESI-QFT	[M+H]+	30	926.51	107.09	99.9	https://massbank.eu/MassBank
Vancomycin	C66H75Cl2N9O64	DB00552	predicted	[M+H]+	40	1450.00	100.11	24.4	https://go.drugbank.com/drugs

Spectra from massbank.eu and drugbank.com. Spectra were selected if recorded in positive ion mode ($[M+H]^+$). Triple-quadrupole mass spectrometer data (LC-ESI-QQ) were preferred over quadrupole/time of flight (LC-ESI-QTOF), and quadrupole/Fourier-transformation (LC-ESI-QFT). Spectra were selected to be recorded with a collision energy of 30 eV or close to 30 eV. Fragmentation prediction in drugbank.com was performed using

competitive fragmentation modelling for metabolite identification (https://cfmid.wishartlab.com). Light blue: Comedications with a similar precursor ion in a ±1 Da range. Dark blue: Comedications with similar transitions in a ±1 Da range.

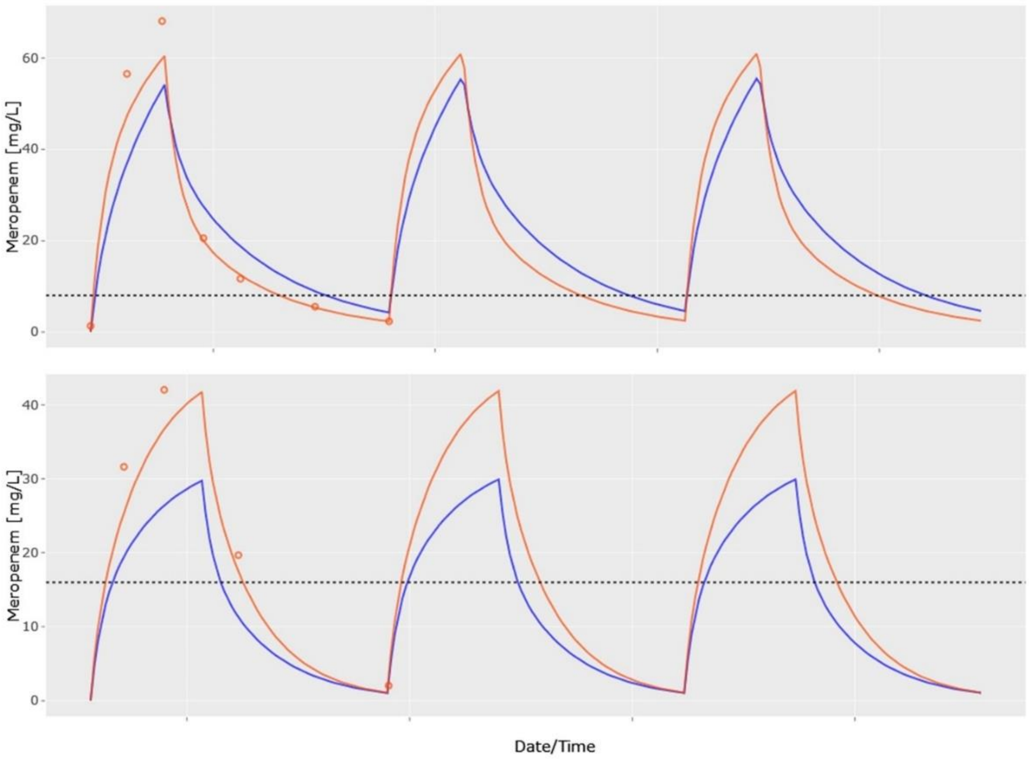

Figure S4. Model based calculation of meropenem %T>MIC using the web-application TDMx.eu. Blue line: population pharmacokinetic; orange circles: measured concentrations; orange line: individually predicted pharmacokinetic; dashed black line: minimal inhibitory concentration (MIC) (2-4).

References

1. Snyder LR, Kirkland JJ, Dolan JW. Basic Concepts and the Control of Separation. Introduction to modern liquid chromatography: John Wiley & Sons; 2009. p. 19-86.
2. Wicha SG. TDMx - Model-Informed Precision Dosing made simple. [Available from: https://tdmx.eu/.
3. Wicha SG, Kees MG, Solms A, Minichmayr IK, Kratzer A, Kloft C. TDMx: a novel web-based open-access support tool for optimising antimicrobial dosing regimens in clinical routine. Int J Antimicrob Agents. 2015;45(4):442-4.
4. Li C, Kuti JL, Nightingale CH, Nicolau DP. Population pharmacokinetic analysis and dosing regimen optimization of meropenem in adult patients. J Clin Pharmacol. 2006;46(10):1171-8.
